# Causal relationship between immune cells and telomere length: mendelian randomization analysis

**DOI:** 10.1186/s12865-024-00610-6

**Published:** 2024-03-08

**Authors:** Yujian Li, Shenglin Lai, Xuan Kan

**Affiliations:** Department of Pediatrics, General Hospital of Tianjin Medical University, No.154, Anshan Road, Heping District, Tianjin, 300052 China

**Keywords:** Mendelian randomization, Immune cell, Telomere length, Genetic causality

## Abstract

**Background:**

The causal relationship between immune cells and telomere length remains controversial.

**Methods:**

Data on the immune cells were obtained from a previous study with 3,757 participants. Data on telomere length were obtained from the OpenGWAS database. Genome-Wide Association Study (GWAS) data were obtained and screened for eligible instrumental variables (IVs) using the TwoSampleMR package and the Phenoscanner database. To investigate the genetic causality between immune cells and telomere length, Mendelian randomization (MR) analysis and Bayesian weighted Mendelian randomization (BWMR) analysis were used.

**Results:**

MR analysis showed that there is indeed a genetic causal relationship between immune cells and telomere length. A total of 16 immune cells were successfully validated. A positive correlation was found between telomere length and immune cells such as CD28 + CD45RA + CD8br %CD8br (*OR* = 1.002, *95%CI*: 1.000-1.003). A negative correlation was found between telomere length and immune cells such as Transitional AC (*OR* = 0.991, *95%CI*: 0.984–0.997) (*P* < 0.05). Reverse MR analysis similarly confirmed that telomere length can affect four types of immune cells, including CD25 on IgD + CD24- (*OR* = 1.291, *95%CI*: 1.060–1.571), at the genetic level.

**Conclusion:**

There is indeed a mutual genetic causality between immune cells and telomere length, which will provide theoretical basis and support for more subsequent clinical studies.

**Supplementary information:**

The online version contains supplementary material available at 10.1186/s12865-024-00610-6.

## Background

Aging is a normal physiological process in the life course of human beings, which is manifested by a decline in the function of cells and multiple systems of the body. When aging causes the function of the immune system to deteriorate, the abnormality of the innate and adaptive immune response, which is known as immunosenescence, can lead to a decrease in the body’s ability to remove toxins and pathogens [1, 2]. It is generally considered that the main cause of immunosenescence is the aging of adaptive immune cells, that is, the aging of T and B lymphocytes. Studies have shown that age-related functional changes in innate immune cells including monocytes-macrophages, neutrophils, dendritic cells and natural killer cells have also been demonstrated [3, 4]. Cellular senescence is recognized as a major cause of aging-related dysfunction [5]. Cellular senescence can be induced by a number of different types of stimuli, and among these factors, telomere shortening is the most significant mechanism of cellular senescence [6].

Telomeres are special ‘cap structure’ at the ends of chromosomes, composed of TTAGGG repeats and specialized proteins. Telomerase can synthesize telomeres, so that the length and structure of telomeres can be stabilized [7, 8]. Telomere length is related to cellular lifespan, and loss of telomere integrity can affect the cell’s ability to replicate and ultimately lead to cellular senescence [9]. Several studies have shown that telomere length is shortened in aged immune cells, which might be one of the reasons why senescence leads to abnormalities in the immune function of body [10]. Unfortunately, there is still some controversy as to whether there is a relationship between immune cells and telomere length, and which specific immune cells are linked to telomere length. Even though there have been some relevant studies, most of them are at a lower level of evidence and susceptible to confounding factors or reverse causation [11]. Taken together, there is a strong need to find new research methods to clarify the genetic causality between immune cells and telomere length.

When applying a routine observational epidemiological study design for etiological inference, the results are often confounded, and the chronological order of exposure and outcome is often confused which is also known as reverse causation, making the etiological explanation unreliable. With the completion of the Human Genome Project and the implementation of the Encyclopedia of DNA Elements program, the study of a variety of diseases and scientific problems has become increasingly linked to epigenetics [12, 13]. MR is a method of epidemiological study design and data analysis based on Mendel’s genetic law, and to demonstrate the etiological hypothesis [14]. MR uses genetic variation to enhance causal inference about a modifiable exposure so as to validate the etiologic hypothesis that environmental exposures are risk factors for disease [15], making it an increasingly powerful tools for epidemiology analysis [16]. BWMR analysis, as a complementary method to two-sample MR analysis, can better take into account the polygenic structure and pleiotropy of the disease or trait, thus improving the stability and reliability of the results of MR analysis to some extent [17]. Finally, based on the validation of the results using the BWMR analysis, we used the *p*.adjust function to correct the results of the BWMR analysis.

Recent study has pointed out that patients with short telomere syndromes are susceptible to cancer due to T cell immunodeficiency [18]. Both CD4 and CD8 T cell senescence have a terminal state of loss of CD28 expression and shortened telomeres [19]. In order to determine the causal relationship between immune cells and telomeres, this study will use MR analysis to analyze the genetic causality between immune cells and telomere length in the organism, so as to provide a theoretical basis for more subsequent clinical studies.

## Materials and methods

### Data acquisition

Samples of the immune cells used as exposure were obtained from previously published articles [20]. A total of 731 immune cells were eventually included in this study. GWAS is a method for detecting polymorphisms in genetic variants across the genome of different individuals through genetics and other methods. The OpenGWAS database (https://gwas.mrcieu.ac.uk/) is a dataset that integrates GWAS data for different diseases, genes, and more. Data for the outcome samples were obtained from the OpenGWAS database.

**Fig. 1:**
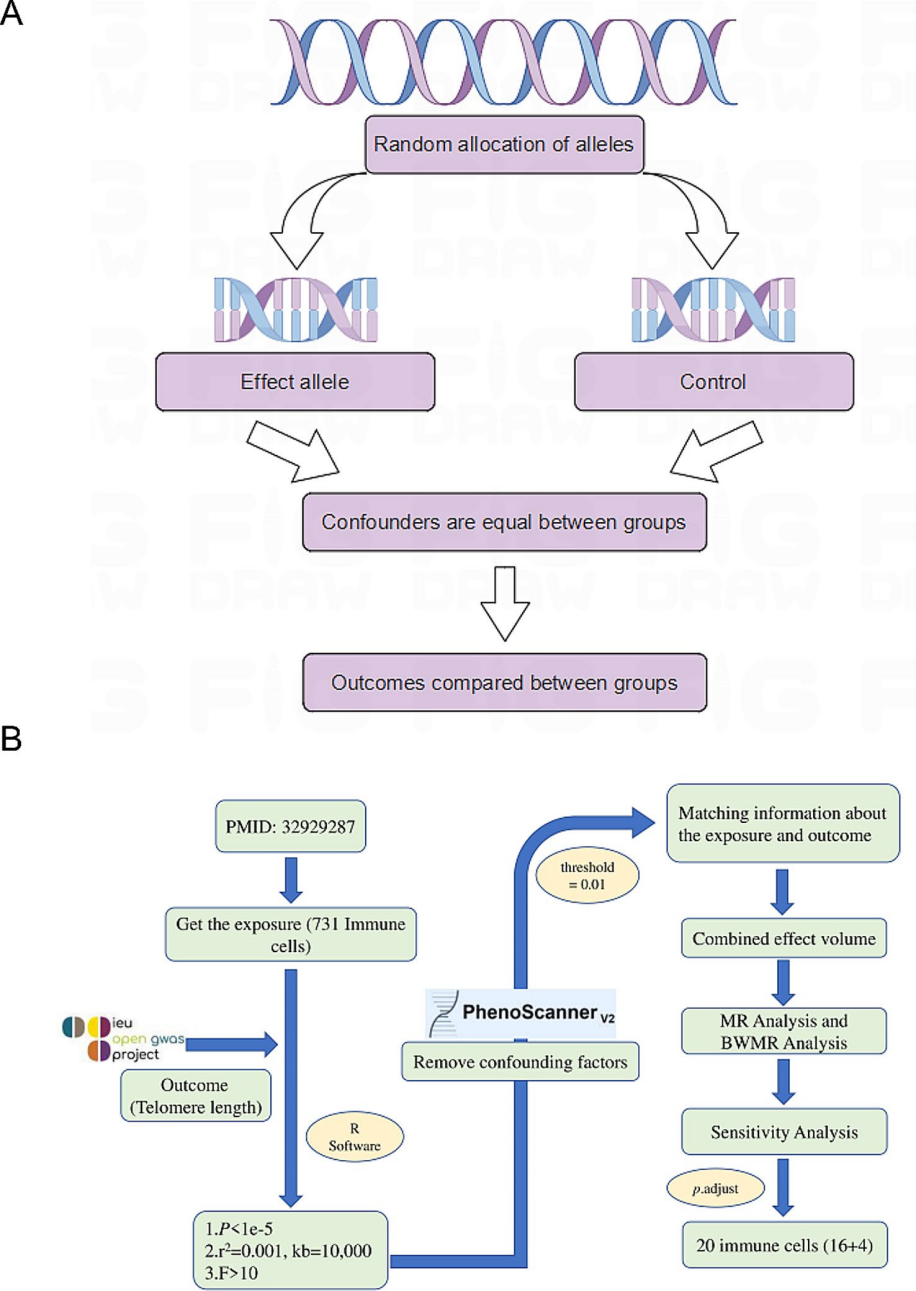
**A**: The schematic diagram. **B**: The flow chart. The whole process is divided into three stages. Phase 1: Immune cells with a genetic causal relationship with telomere length are identified from 731 immune cells by two-sample MR analysis. Phase 2: Use BWMR analysis to validate the results of MR analysis. Phase 3: Use the *p*.adjust function to correct the results of the BWMR analysis. MR: mendelian randomization. BWMR: Bayesian weighted mendelian randomization

Data from exposure (immune cells) and outcome (telomere length) samples were extracted with the help of the TwoSampleMR package. Figure 1 shows the schematic diagram and flow chart.

### Data screening

In order to obtain more reliable IVs to increase the reliability of the results, the IVs need to fulfill four screening conditions simultaneously. Firstly, the IVs need to be relevant to the exposure (*P* < 1e-5). Based on this, we firstly excluded SNPs that were weakly correlated or even uncorrelated with exposure (*P* > 1e-5). Then, MR analysis need to exclude linkage disequilibrium (LD) in the IVs (Screening criteria: r^2^ > 0.001, kb = 10,000). Thirdly, the IVs used for MR analysis need to have sufficient strength of association with the exposure (Screening criteria: F > 10) [21, 22]. Finally, MR analysis also need to remove confounders of IVs with the help of the Phenoscanner database (http://www.phenoscanner.medschl.cam.ac.uk/) [23]. We screened and eliminated SNPs that could affect outcome with the help of the Phenoscanner database. When using telomere length as the exposure object and immune cells as the outcome object for reverse MR analysis, the screening of IVs also needs to fulfill the above four conditions. Because too many IVs were obtained when telomere length was used as the exposure object, we set the screening conditions for the correlation hypothesis more stringently (*P* < 5e-8).

R software and TwoSampleMR package were used to match the effect alleles of the exposure and outcome [24].

### MR analysis and BWMR analysis

The genetic causality between 731 immune cells and telomere length was analyzed by MR analysis using each of the five methods, inverse variance weighted (IVW), simple mode, weighted median, weighted mode and MR-Egger. Among the above five methods, IVW and MR-Egger were the most important for determining the results, and genetic causality was considered to exist between the corresponding immune cells and telomere length when *P* < 0.05 [25]. Afterwards, we used immune cells with positive results as the outcome object and telomere length as the exposure object to perform reverse MR validation in order to further improve the reliability and practical significance of this study. BWMR analysis was a complement to two-sample MR analysis. On the one hand, it took into account the polygenic structure and pleiotropy of diseases or traits not considered by MR analysis, on the other hand, it improved the stability and reliability of the final results [17]. Finally, the *p*.adjust function was used for the correction of the results of the BWMR analysis. Correlations with *P* < 0.05 for the raw results while *P* > 0.05 after adjustion were considered suggestive, whereas correlations with *P* < 0.05 after adjustion were considered significant.

### Sensitivity analysis

Heterogeneity test, horizontal multiple validity test and leave-one-out sensitivity analysis were used to validate the positive results of the MR analysis with a view to further improving the confidence of the results. For positive results, the heterogeneity test was first used to confirm the existence of heterogeneity between each IV. When *P* > 0.05, it was considered that there was no heterogeneity between IVs and the results of MR analysis were presented using a fixed-effects model. When *P* < 0.05, it was considered that there was heterogeneity between IVs and it was necessary to present the final results using a random-effects model [26]. Second, leave-one-out sensitivity analysis was used to test the effect of individual IVs on the overall results of MR analysis [27]. Finally, since the MR-Egger method took into account the intercept term when performing the regression analysis, we used the horizontal multiple validity test to compare the MR-Egger method and the IVW method in order to verify the existence of horizontal pleiotropy between IVs. When *P* > 0.05, it is considered that there is no horizontal pleiotropy between IVs and the IVW method is used as the result of MR analysis. When *P* < 0.05, it is considered that there is horizontal pleiotropy between IVs and the result of the MR-Egger method is used as the demonstrated result [28].

## Results

### Samples and data

The immune cells were sampled from 3,757 participants primarily from the Sardinians. SNPs are DNA sequence polymorphisms that are widespread in the human genome, and are among the most common types of heritable variation in humans. The most recent published GWAS data with the largest sample size in the OpenGWAS database were selected as the exposure to improve the reliability and real-time performance of the results. The GWAS ID for telomere length was ieu-b-4879, and the study population was from Europe, containing a total of 20,134,421 SNPs (Table 1).

**Table 1:** Baseline information table for exposure and outcome

Subject	Year	Author	Population	Sample size	ID	Links for data download
Exposure						
Immune cells	2020	Orrù V	Sardinians	3,757	PMID: 32,929,287	http://ftp.ebi.ac.uk/pub/databases/gwas/summary_statistics/
Outcome						
Telomere length	2021	Codd	European	472,174	GWAS ID: ieu-b-4879	https://gwas.mrcieu.ac.uk/datasets/ieu-b-4879/

#### MR analysis and BWMR analysis

**Fig. 2:**
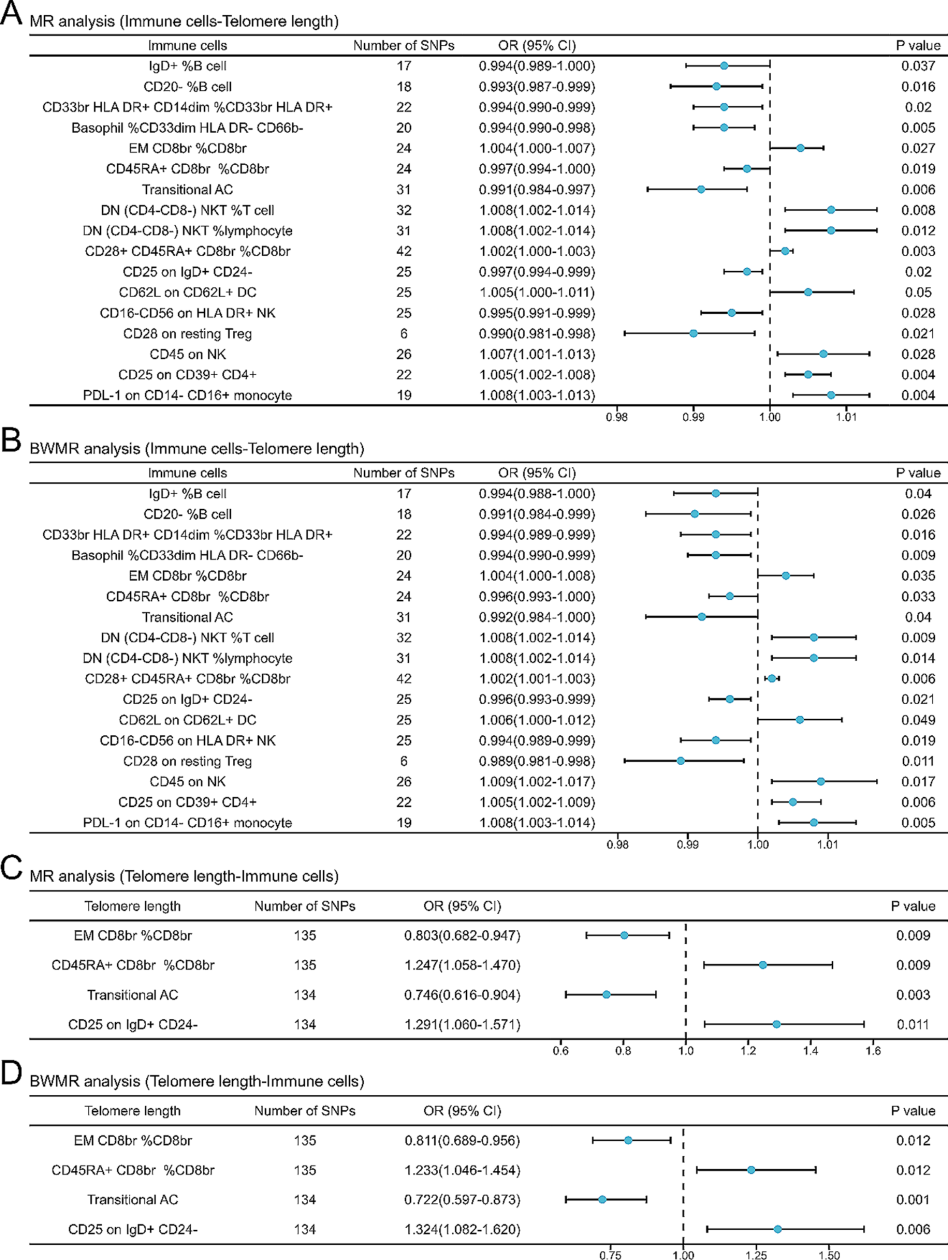
Forest plot of MR analysis and BWMR analysis. **A**: MR analysis (immune cells-telomere length). **B**: BWMR analysis (immune cells-telomere length). After two-sample MR analysis combined with BWMR analysis, a total of 17 immune cells affecting telomere length were obtained. Seven immune cells delayed telomere shortening, while the other ten immune cells promoted telomere length shortening. **C**: MR analysis (telomere length -immune cells). **D**: BWMR analysis (telomere length-immune cells). Telomeres have the potential to promote the expression of two types of immune cells, meanwhile, the expression of two types of immune cells may be suppressed. MR: mendelian randomization. BWMR: Bayesian weighted mendelian randomization

MR analysis of 731 immune cells and telomere length revealed a total of 20 immune cells with a causal relationship with telomere length. Reverse MR analysis showed that telomere length similarly affects at least five types of immune cells at the genetic level. We validated the MR analysis results using BWMR analysis. Result turned out that there were 17 immune cells which can affect telomere length, in which seven of them delayed telomere shortening, while the other ten immune cells promoted telomere length shortening. Finally, we corrected the results of the BWMR analysis using the *p*.adjust function, which showed that a total of 16 immune cells affecting telomere length, and that telomere length could in turn affect four of these immune cells. A total of 496 SNPs from 20 immune cells and 140 SNPs from telomere length were included in the final analysis (Table S1). The results of MR analysis combined with BWMR analysis showed positive genetic causal relationship between telomere length and seven immune cells, including EM CD8br %CD8br (*OR* = 1.004, *95%CI*: 1.000-1.007), DN (CD4-CD8-) NKT %T cell (*OR* = 1.008, *95%CI*: 1.002–1.014), DN (CD4-CD8-) NKT %lymphocyte (*OR* = 1.008, *95%CI*: 1.002–1.014), CD28 + CD45RA + CD8br %CD8br (*OR* = 1.002, *95%CI*: 1.000-1.003), CD45 on NK (*OR* = 1.007, *95%CI*: 1.001–1.013), CD25 on CD39 + CD4+ (*OR* = 1.005, *95%CI*: 1.002–1.008) and PDL-1 on CD14- CD16 + monocyte (*OR* = 1.008, *95%CI*: 1.003–1.013). In addition, there was a negative genetic causal relationship between telomere length and nine immune cells, including IgD+ %B cell (*OR* = 0.994, *95%CI*: 0.989-1.000), CD20- %B cell (*OR* = 0.993, *95%CI*: 0.987–0.999), CD33br HLA DR + CD14dim %CD33br HLA DR+ (*OR* = 0.994, *95%CI*: 0.990–0.999), Basophil %CD33dim HLA DR- CD66b- (*OR* = 0.994, *95%CI*: 0.990–0.998), CD45RA + CD8br %CD8br (*OR* = 0.997, *95%CI*: 0.994-1.000), Transitional AC (*OR* = 0.991, *95%CI*: 0.984–0.997), CD25 on IgD + CD24- (*OR* = 0.997, *95%CI*: 0.994–0.999), CD16-CD56 on HLA DR + NK (*OR* = 0.995, *95%CI*: 0.991–0.999) and CD28 on resting Treg (*OR* = 0.990, *95%CI*: 0.981–0.998) (*P* < 0.05). Reverse MR analysis showed that longer telomere could enhance the effects of CD45RA + CD8br %CD8br (*OR* = 1.247, *95%CI*: 1.058–1.470) and CD25 on IgD + CD24- (*OR* = 1.291, *95%CI*: 1.060–1.571). In addition, longer telomere length may also diminish the role of EM CD8br %CD8br (*OR* = 0.803, *95%CI*: 0.682–0.947) and Transitional AC (*OR* = 0.746, *95%CI*: 0.616–0.904) (Fig. 2; Table 2, Figure S1, S3).

**Table 2:** Mendelian randomization analysis and sensitivity analysis

Exposure-Outcome	No. SNP	MR analysis		Heterogeneity		Pleiotropy		BWMR analysis		
		OR(95%CI)	P	Q	P	Intercept	P	OR(95%CI)	P	FDR
Immune cells-Telomere length										
IgD+ %B cell	17	0.994(0.989-1.000)	0.037	17	0.402	-0.002	0.152	0.994(0.988-1.000)	0.040	0.050
CD20- %B cell	18	0.993(0.987-0.999)	0.016	17	0.452	0.000	0.826	0.991(0.984-0.999)	0.026	0.043
IgD+ CD38- %lymphocyte	20	0.995(0.991-1.000)	0.039	18	0.544	0.001	0.198	0.996(0.988-1.005)	0.398	0.398
CD25hi %CD4+	26	0.994(0.988-1.000)	0.040	34	0.098	-0.002	0.058	0.993(0.987-1.000)	0.053	0.056
CD25hi CD45RA- CD4 not Treg %T cell	32	0.996(0.993-1.000)	0.044	31	0.447	0.000	0.816	0.996(0.992-1.000)	0.053	0.056
CD33br HLA DR+ CD14dim %CD33br HLA DR+	22	0.994(0.990-0.999)	0.020	20	0.530	-0.001	0.182	0.994(0.989-0.999)	0.016	0.038
Basophil %CD33dim HLA DR- CD66b-	20	0.994(0.990-0.998)	0.005	18	0.531	0.000	0.917	0.994(0.990-0.999)	0.009	0.036
EM CD8br %CD8br	24	1.004(1.000-1.007)	0.027	18	0.775	0.000	0.861	1.004(1.000-1.008)	0.035	0.050
CD45RA+ CD8br %CD8br	24	0.997(0.994-1.000)	0.019	17	0.825	0.000	0.949	0.996(0.993-1.000)	0.033	0.050
Transitional AC	31	0.991(0.984-0.997)	0.006	43	0.055	-0.002	0.126	0.992(0.984-1.000)	0.040	0.050
DN (CD4-CD8-) NKT %T cell	32	1.008(1.002-1.014)	0.008	38	0.194	0.001	0.670	1.008(1.002-1.014)	0.009	0.036
DN (CD4-CD8-) NKT %lymphocyte	31	1.008(1.002-1.014)	0.012	37	0.170	-0.001	0.535	1.008(1.002-1.014)	0.014	0.038
CD28+ CD45RA+ CD8br %CD8br	42	1.002(1.000-1.003)	0.003	54	0.079	0.000	0.584	1.002(1.001-1.003)	0.006	0.036
CD25 on IgD+ CD24-	25	0.997(0.994-0.999)	0.020	30	0.177	0.000	0.967	0.996(0.993-0.999)	0.021	0.038
CD62L on CD62L+ DC	25	1.005(1.000-1.011)	0.050	19	0.733	-0.001	0.488	1.006(1.000-1.012)	0.049	0.056
CD16-CD56 on HLA DR+ NK	25	0.995(0.991-0.999)	0.028	21	0.656	-0.001	0.153	0.994(0.989-0.999)	0.019	0.038
CD28 on resting Treg	6	0.990(0.981-0.998)	0.021	7	0.258	0.000	0.977	0.989(0.981-0.998)	0.011	0.037
CD45 on NK	26	1.007(1.001-1.013)	0.028	31	0.180	-0.001	0.521	1.009(1.002-1.017)	0.017	0.038
CD25 on CD39+ CD4+	22	1.005(1.002-1.008)	0.004	28	0.143	0.002	0.140	1.005(1.002-1.009)	0.006	0.036
PDL-1 on CD14- CD16+ monocyte	19	1.008(1.003-1.013)	0.004	17	0.546	0.000	0.886	1.008(1.003-1.014)	0.005	0.036
Telomere length-Immune cells										
EM CD8br %CD8br	135	0.803(0.682-0.947)	0.009	126	0.683	0.005	0.225	0.811(0.689-0.956)	0.012	0.015
CD45RA+ CD8br %CD8br	135	1.247(1.058-1.470)	0.009	117	0.852	-0.006	0.200	1.233(1.046-1.454)	0.012	0.015
Transitional AC	134	0.746(0.616-0.904)	0.003	143	0.260	0.001	0.780	0.722(0.597-0.873)	0.001	0.004
CD28+ CD45RA+ CD8br %CD8br	135	1.169(1.006-1.359)	0.042	144	0.266	-0.006	0.105	1.155(0.992-1.345)	0.064	0.064
CD25 on IgD+ CD24-	134	1.291(1.060-1.571)	0.011	142	0.283	0.003	0.524	1.324(1.082-1.620)	0.006	0.015

MR: Mendelian randomization

### Sensitivity analysis

The results of the test of heterogeneity and the test of horizontal pleiotropy showed that *P* > 0.05, so it can be concluded that there is no heterogeneity and horizontal pleiotropy among the IVs in this study. Finally, the results of IVW analysis were used as the results of MR analysis and fixed effect model was used to show the final results (Table 2, Figure S2, S4).

## Discussion

Aging, as a natural process that cannot be avoided by the organism, has been studied by the scientific community for a long time. Immune cells and telomere wear and tear are thought to play a very important role in the aging process of the organism [29, 30]. In addition, some studies have shown a correlation between immune cells and telomeres in some cancers or hematologic disorders [31, 32]. Despite of some description of telomerase loss and telomere shortening in immune senescence, the molecular mechanisms of influencing telomerase expression have not been elucidated [33]. Although some studies have elucidated that CD28 costimulatory signals can up-regulate telomerase activity and maintenances telomere length in T cell replicative senescence in human aging [19], the more detailed molecular mechanisms, as well as the mechanisms by which other immune cells affect telomeres, are still unclear. In this study, the relationship between immune cells and telomere length was investigated from a genetic perspective using MR analysis, which reduced the influence of confounding factors and ethical considerations that cannot be avoided by conventional epidemiological statistical methods to a certain extent. At the same time, we also used reverse MR analysis and BWMR analysis, which increased the depth of the study and further improved the reliability of the results.

In this study, among seven groups of immune cells which contained B cells, cDCs, maturation of T cells, monocytes, myeloid cells, TBNK (T cells, B cells, natural killer cells) and Treg, telomere length was found to have causal effects on 16 immunophenotypes. PDL-1 on CD14- CD16 + monocyte in the monocyte panel, as well as most of the immunophenotypes of TBNK and Treg were positively correlated with telomere length. It was noteworthy that among these 16 immune cells, the immunophenotypes of all four B cells and two of myeloid cells were negatively correlated with telomere length. In the maturation stages of T cell panel, EM CD8br %CD8br was positively correlated with telomere length while CD45RA + CD8br %CD8br was negatively correlated with telomere length. Interestingly, we found that there was one immunophenotype, positively correlated with telomere length, and one immunophenotype, negatively correlated with telomere length, in B cells panel and maturation of T cells panel, respectively. MR analysis identified some of the B cells, Treg and Myeloid cells, etc., and most of them had negative genetic causality with telomere length. The results of MR analysis showed a more pronounced negative correlation between Transitional AC in B cells and CD28 on resting Treg in Treg with telomere length. In contrast, CD28 + CD45RA + CD8br %CD8br in Treg and most of TBNK showed a positive correlation with telomere length. Previous studies have shown that CD8 + CD28- T cell senescence is triggered by a variety of biological processes including telomere damage, Treg cells and tumor-associated stresses [34]. Having a higher frequency of more differentiated CD28 − CD8 + T cells was associated with shorter telomere length [35]. These studies confirm that there is indeed a relationship between T cells, especially Treg, and telomere length. It is possible that immune cells such as T cells could affect telomere length or its expression by influencing telomerase activity. Kawano Y’s study showed shorter Treg telomere length and increased Treg telomerase activity compared to Tcon [36]. The Vukmanovic-Stejic M’s study similarly found that the proliferating CD4 Treg have relatively short telomeres and also exhibit increased susceptibility to apoptosis [37]. In addition, Lansdorp PM’s research shows that a third driver of the aging process is the progressive loss of telomeric DNA, but that this process plays an important role in preventing cancer early in life [38]. Studies of tumor patients have similarly found that the number of circulating Treg is often increased in tumor patients. In this case, the telomeres of proliferating Treg are shortened despite increased telomerase levels [39]. These studies have again validated the relationship between immune cells such as T cells and telomere length, as well as the role of telomerase in the relationship between immune cells and telomere length.

Telomere maintenance and telomerase regulation are closely linked to the activation and differentiation of T and B cells: telomerase activity in resting T and B cells decreases as they progress from naïve to memory cells, and is upregulated upon antigen stimulation [40]. In addition, the difference in telomere length between CML blast cells relative to T lymphocytes in the same patient correlates with disease progression [41]. In addition to aging, tumors are likewise a long-lasting research hotspot. Studies on the correlation between telomere length and tumors have found that telomeres play an important role in cellular value-addition and cellular carcinogenesis. The telomerase gene hTERT plays an indispensable role in the pathogenesis of leukemia and is mainly associated with B-lymphocytes [42]. It follows that there is a similar correlation between immune cells such as B cells and Myeloid cells and telomeres. Reverse MR analysis combined with BWMR analysis revealed that a total of 4 immune cells were successfully validated to be affected genetically by telomere length. Among them, Transitional AC in B cells was confirmed to be consistent with the results of forward MR analysis. Reverse MR analysis also revealed a positive correlation between telomere length and CD25 on IgD + CD24- in B cells. Previous studies have found that B cells have the highest telomerase activity and longest telomere length among CD4 + T cells, CD8 + CD28 + T cells, CD8 + CD28 − T cells as well as B cells, which corroborates with the results of the present study [43].

In this study, the causal relationship between immune cells and telomere length in the organism was studied and investigated by MR analysis combined with BWMR analysis for the first time. Because MR analysis can better eliminate confounding factors and reduce the interference of ethical factors than traditional epidemiological statistical methods, the conclusions of this study are more reliable than those of previous studies. In addition, the most recent published GWAS data with the largest sample size in the OpenGWAS database were selected, which further improved the reliability of the findings. Finally, the immune cell species used for analysis in this study reached as many as 731, and both forward and reverse validation were also carried out, making the conclusions more reliable and providing some ideas and directions for this subsequent related research. This study also has some limitations. Firstly, there may be some selection bias as the samples of immune cells were mainly from Sardinians and the samples of telomere length were only from European populations. Although MR analysis can randomize the characteristics of the study subjects, due to the limited size of the immune cell samples, we still cannot ignore the bias on the results caused by the background of the study subjects, such as age, gender, and ethnicity. Secondly, although the screening of the samples and IVs in this study was strictly regulated in order to obtain more reliable results, there is a lack of basic research to further confirm this, and more in-depth studies could be carried out from a functional and mechanistic point of view. Finally, due to the limitation of the study sample, it is a great pity that this study failed to carry out further subgroup analysis. Subsequently, subgroup analysis can be carried out from the perspectives of age, ethnicity, gender, and etc. by collecting more data when the condition permits, so as to further improve the generalizability of the results.

In conclusion, MR analysis and BWMR analysis revealed a genetic causality between some immune cells and telomere length. Immune cells such as CD28 + CD45RA + CD8br %CD8br were positively correlated with telomere length, whereas other immune cells such as transitional AC were negatively correlated with telomere length. Reverse MR analysis similarly confirmed that telomere length can affect four types of immune cells, including EM CD8br %CD8br at the genetic level. Based on this, we believe that further research on the correlation between immune cells and telomere length is needed to provide some new directions and ideas for the exploration of scientific issues such as aging.

### Electronic supplementary material

Below is the link to the electronic supplementary material.

Supplementary Material 1

Supplementary Material 2

Supplementary Material 3

Supplementary Material 4

Supplementary Material 5

Supplementary Material 6

## Data Availability

The data sets analyzed during the current study are available in a previous article (http://ftp.ebi.ac.uk/pub/databases/gwas/summary_statistics/) (accession nos. PMID: 32929287) and OpenGWAS database (https://gwas.mrcieu.ac.uk/) (accession nos. fieu-b-4879).

## Abbreviations

GWAS: Genome-Wide Association Study
IVs: instrumental variables
MR: Mendelian randomization
BWM: Bayesian weighted Mendelian randomization
CD: cluster of differentiation
AC: absolute cell count
LD: linkage disequilibrium
IVW: inverse variance weighted
SNPs: single nucleotide polymorphisms
EM: effector memory
DN: double negative
NK: natural killer
TBNK: T cells, B cells and natural killer cells
HLA: human leukocyte antigen
IgD: immunoglobulin D
Treg: regulatory T
Tcon: conventional T cell
CML: chronic myeloid leukemia
