# Supplementary figures and images for: Causal relationship between immune cells and telomere length: mendelian randomization analysis
Source: BMC Immunol. 2024 Mar 8;25:19. doi: 10.1186/s12865-024-00610-6 (PMC10924351; doi:10.1186/s12865-024-00610-6)

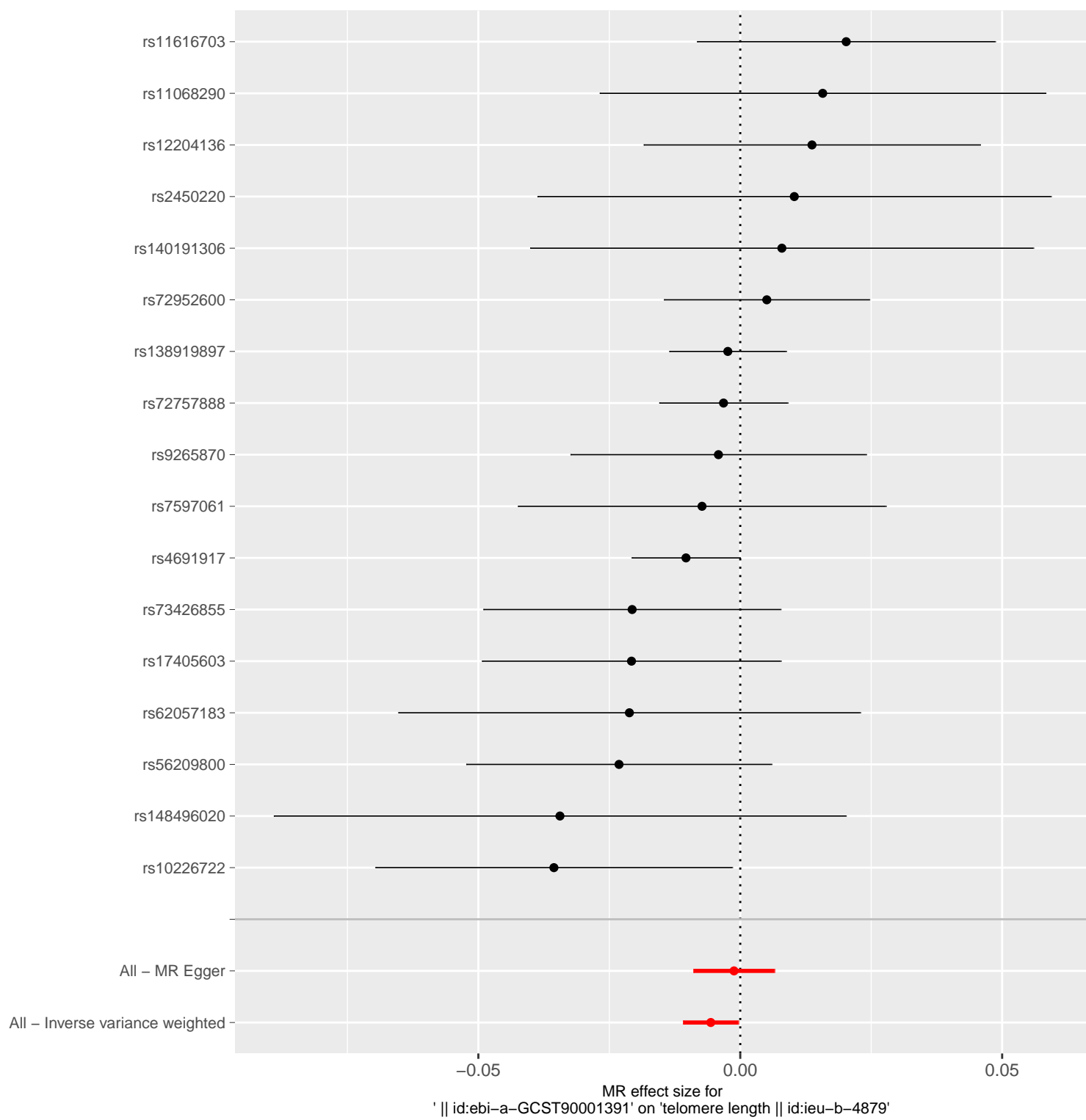

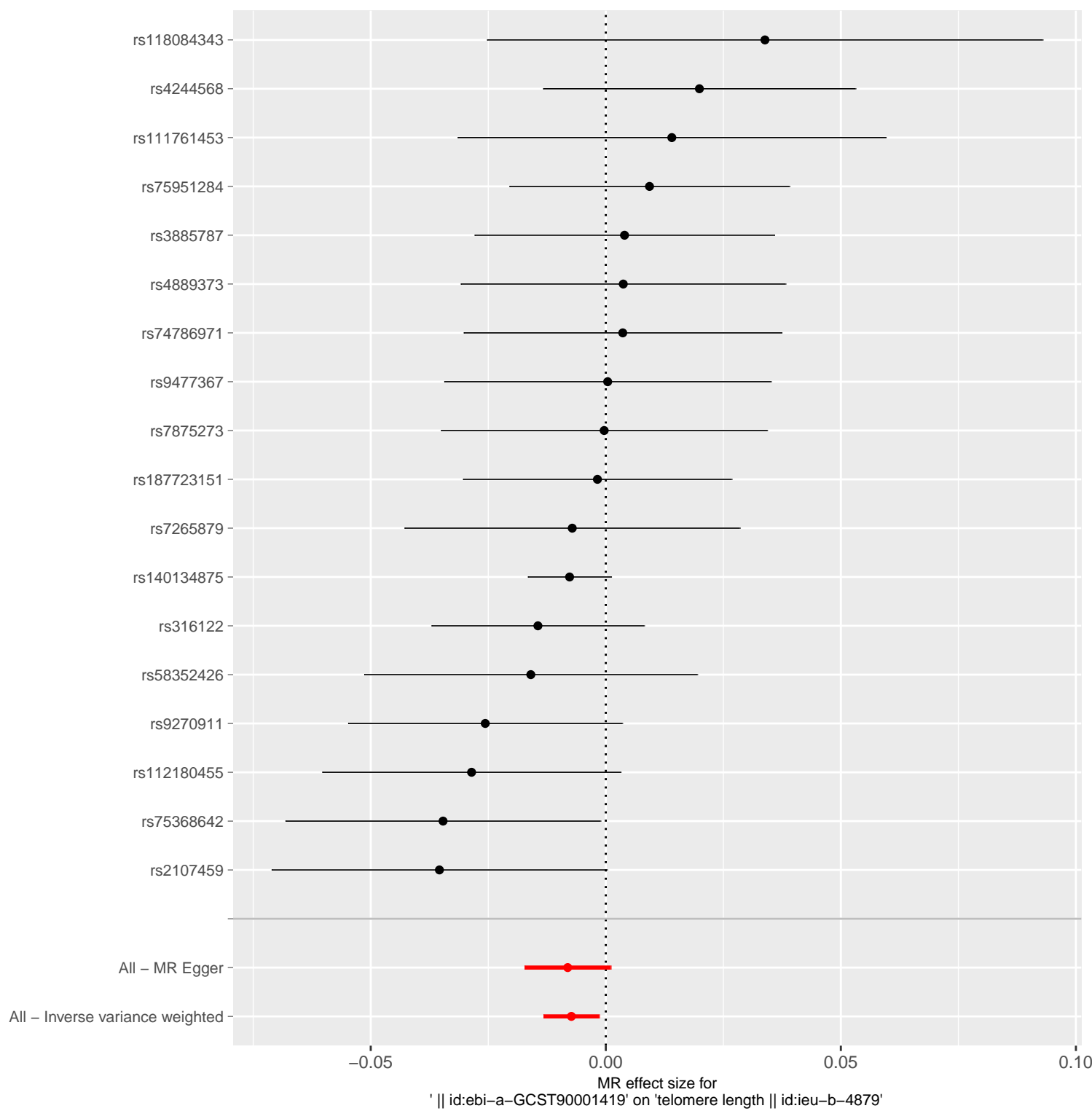

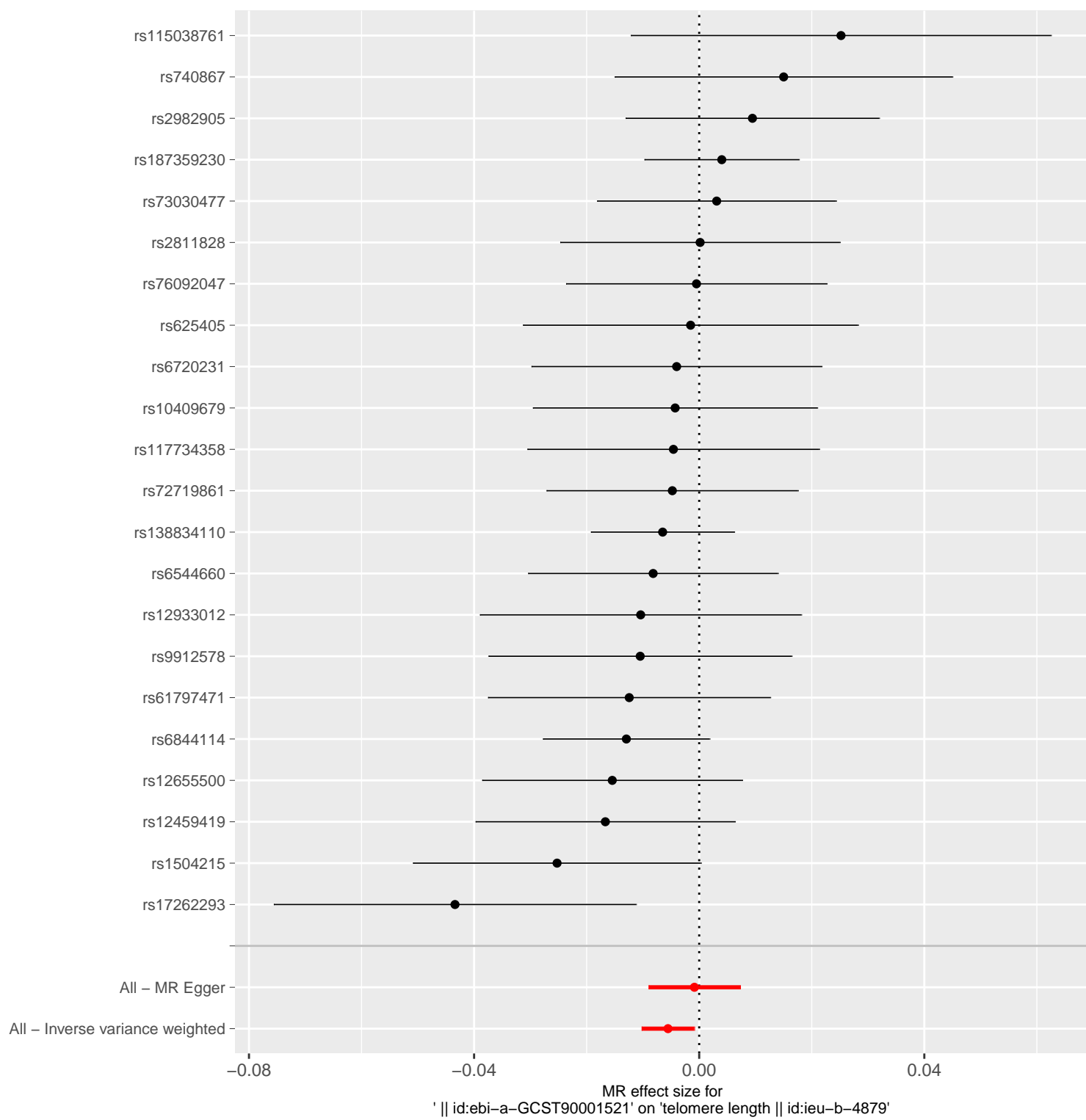

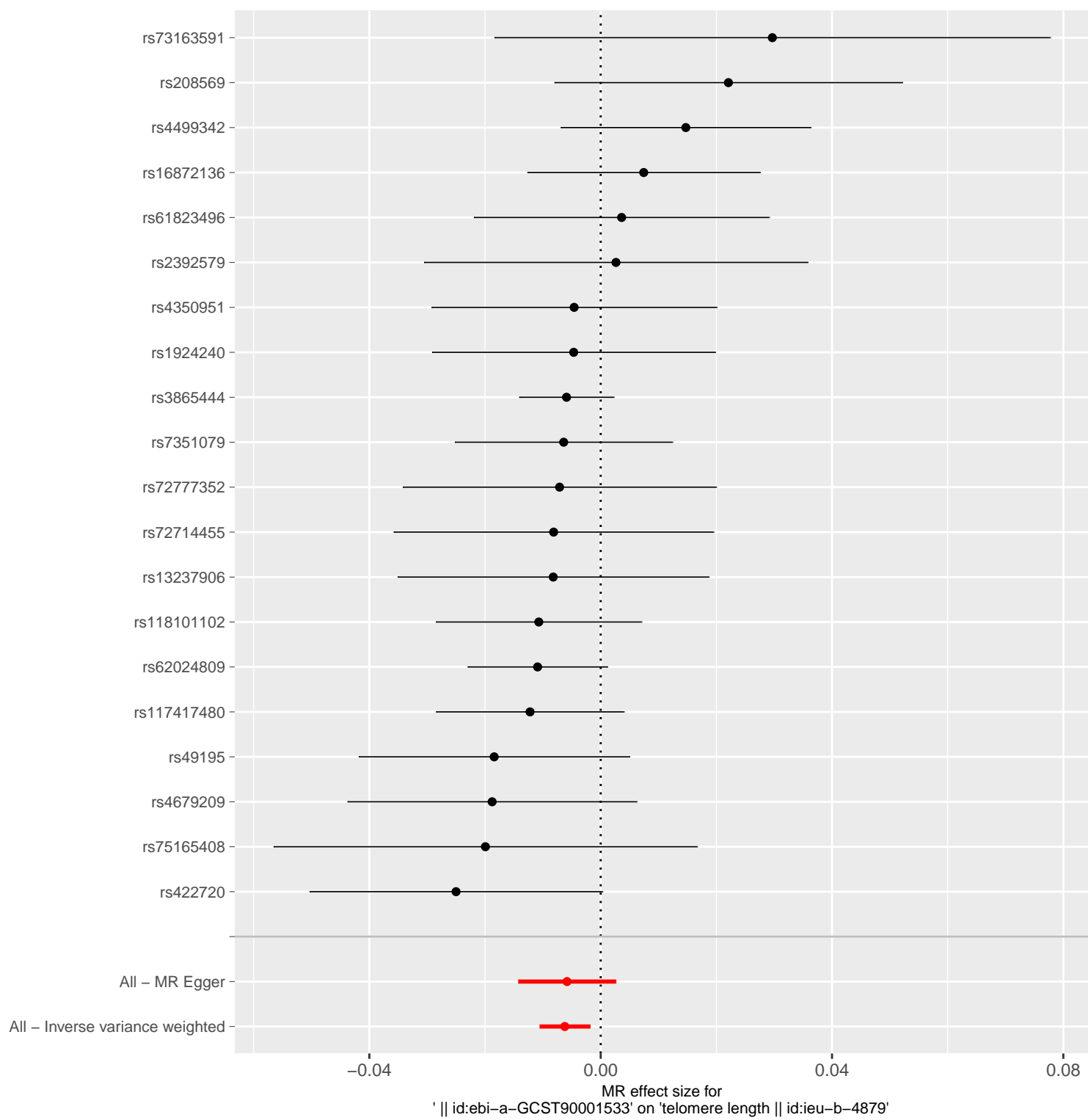

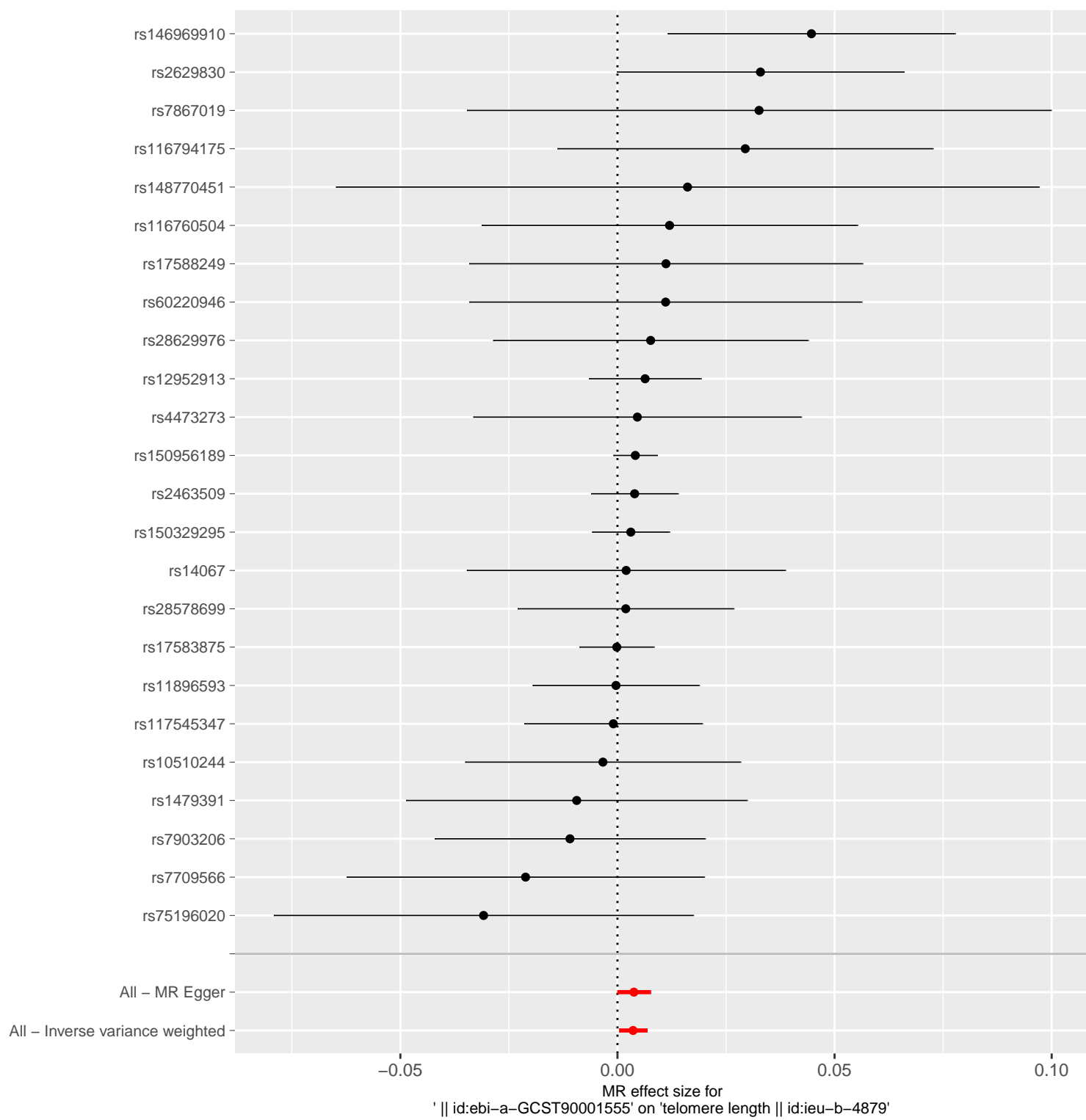

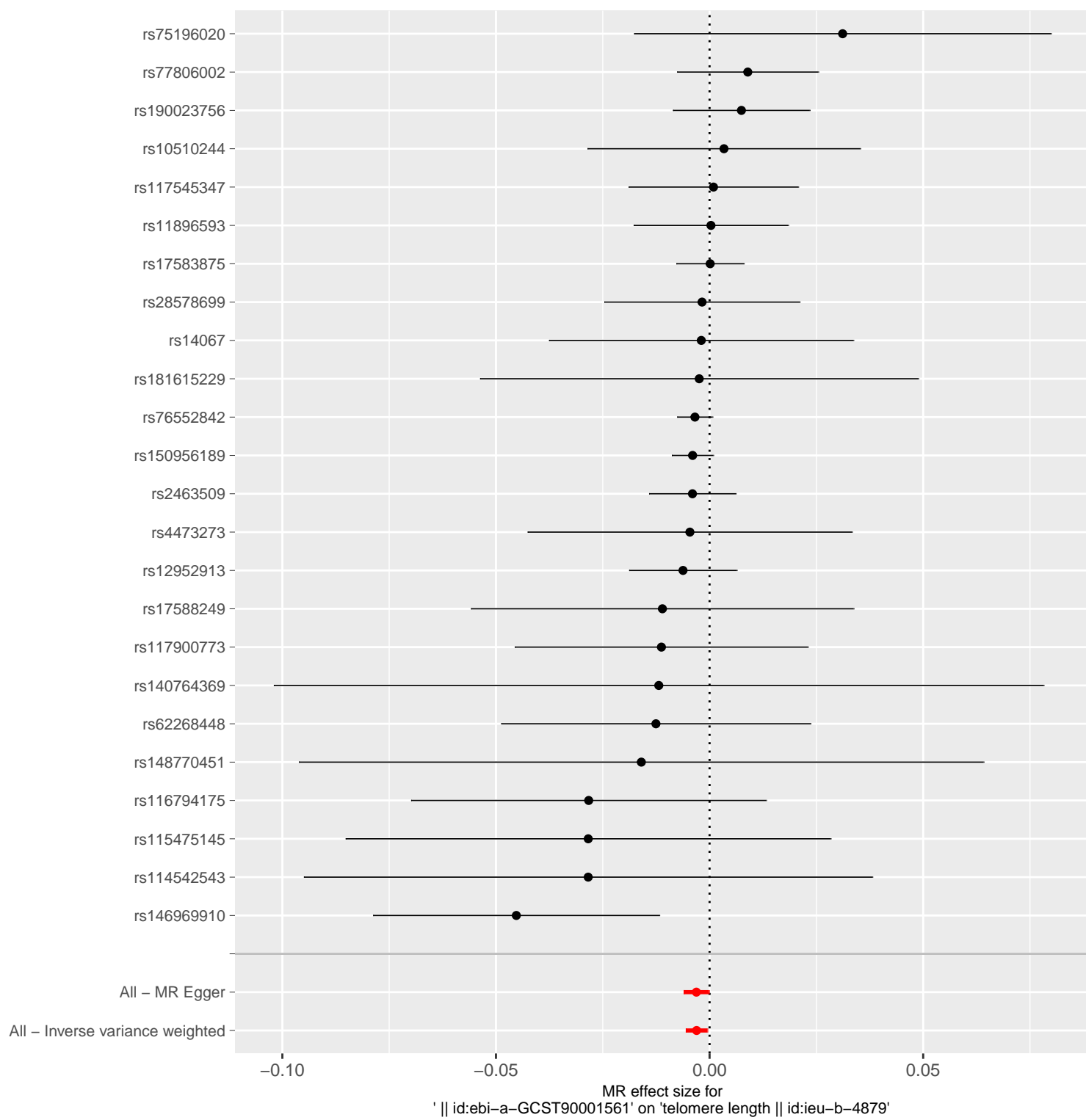

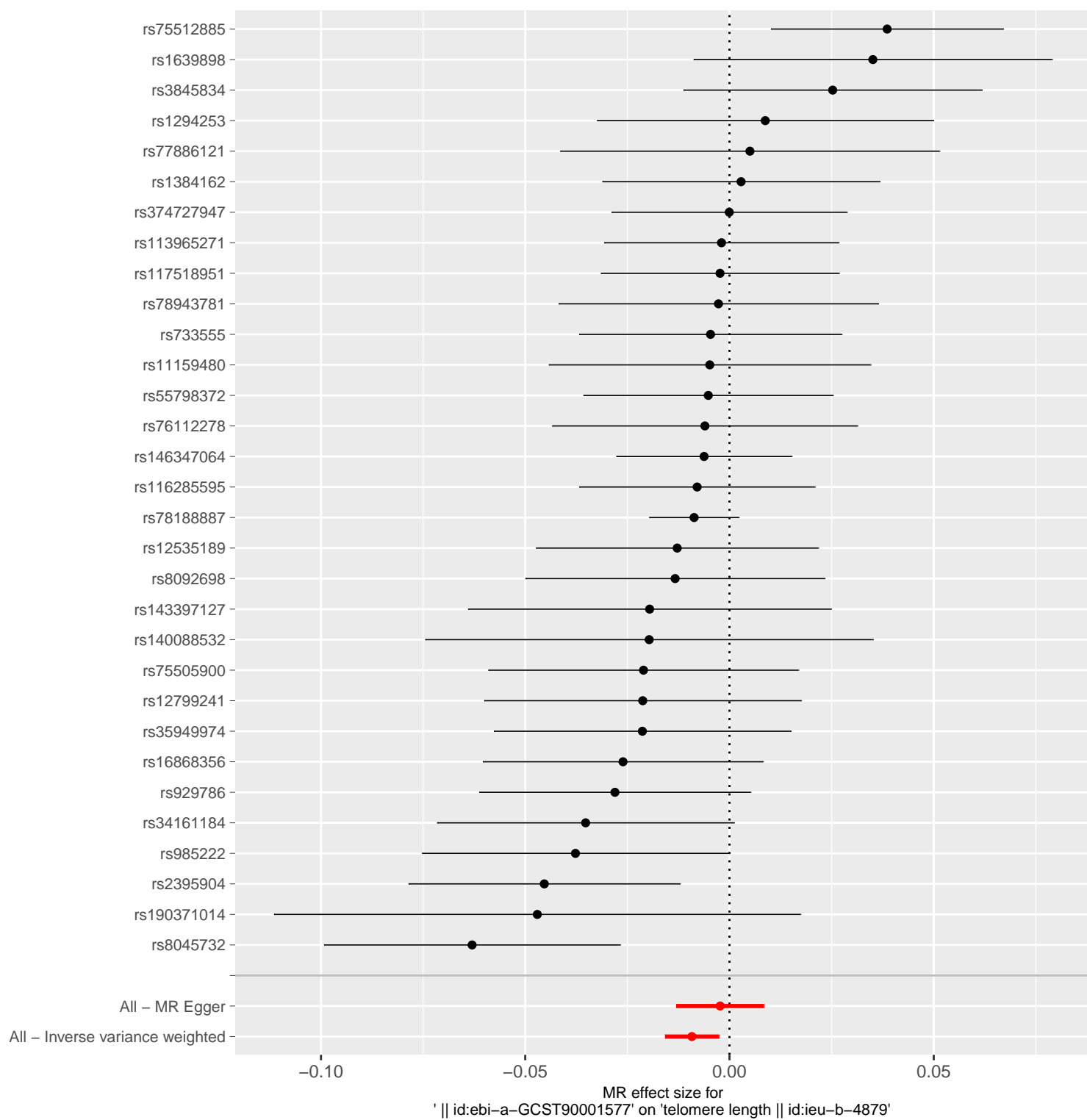

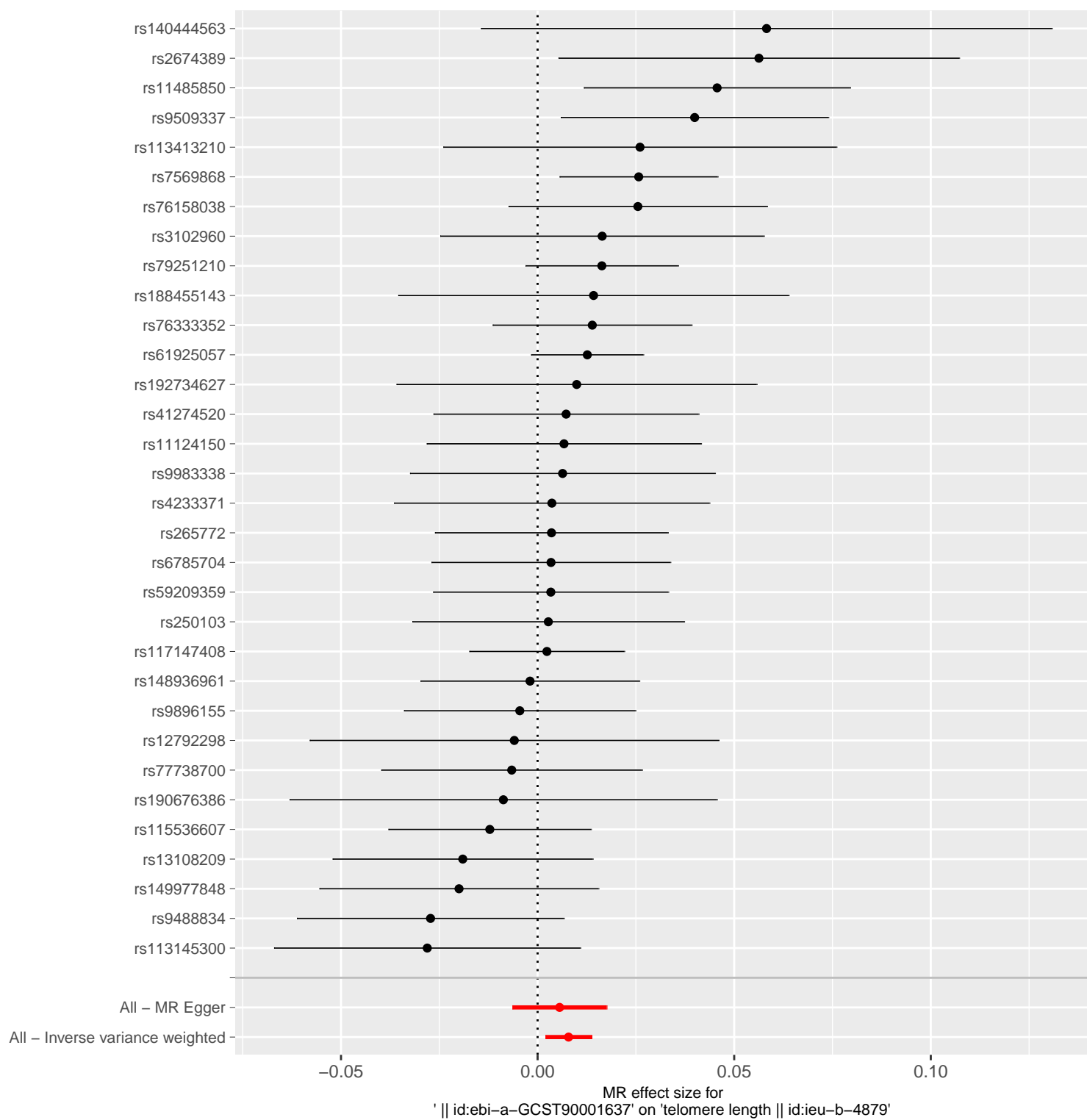

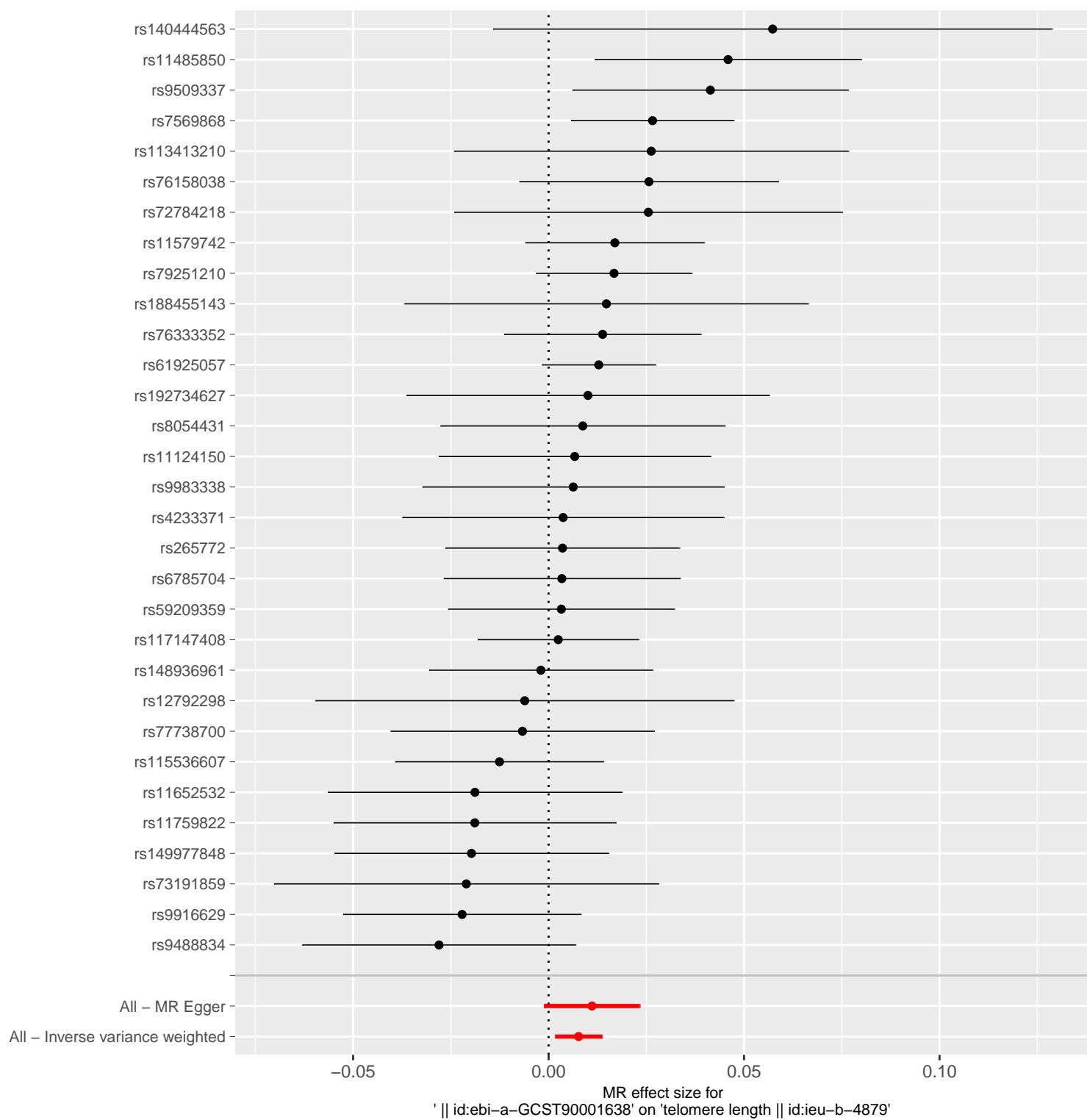

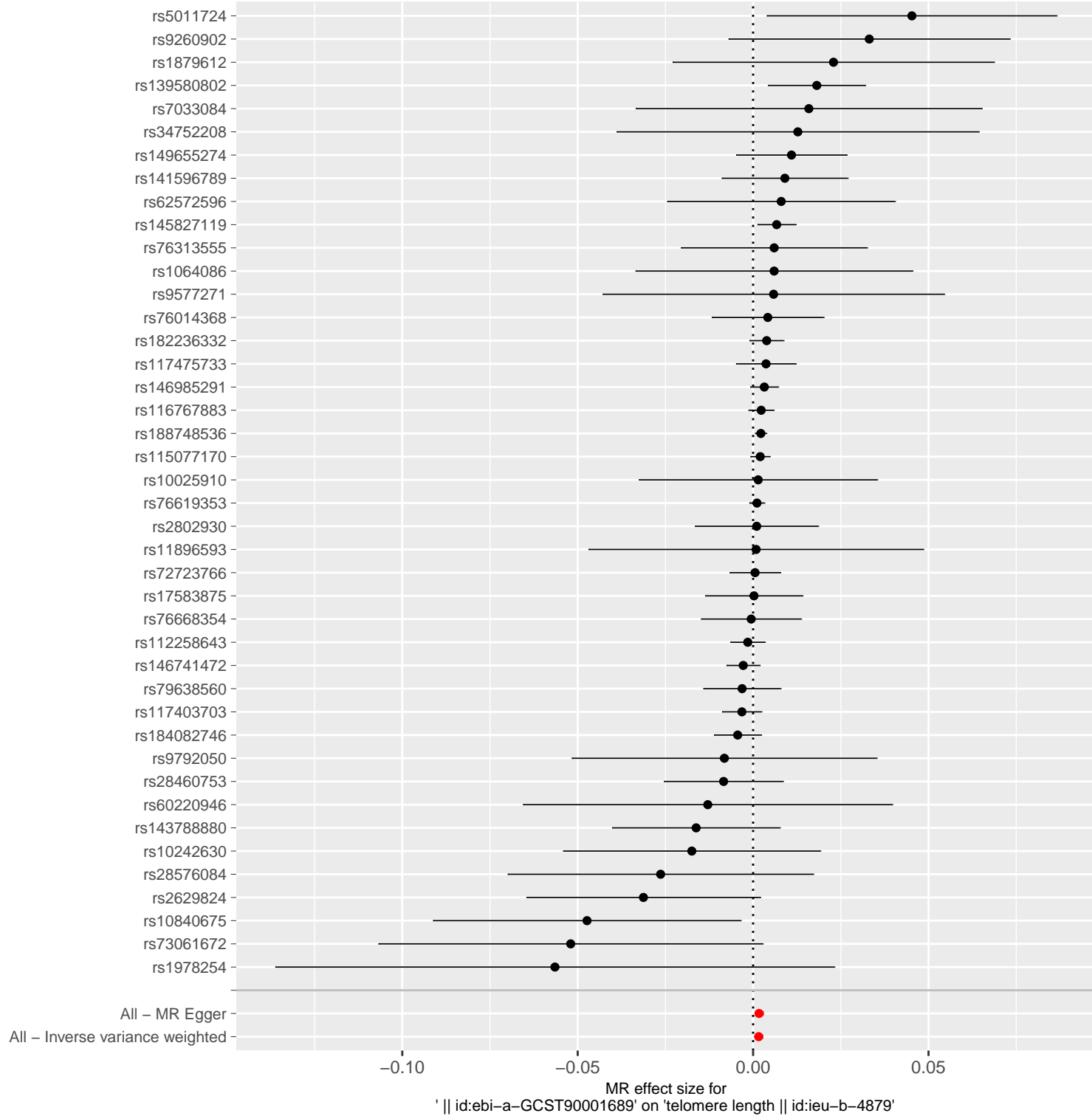

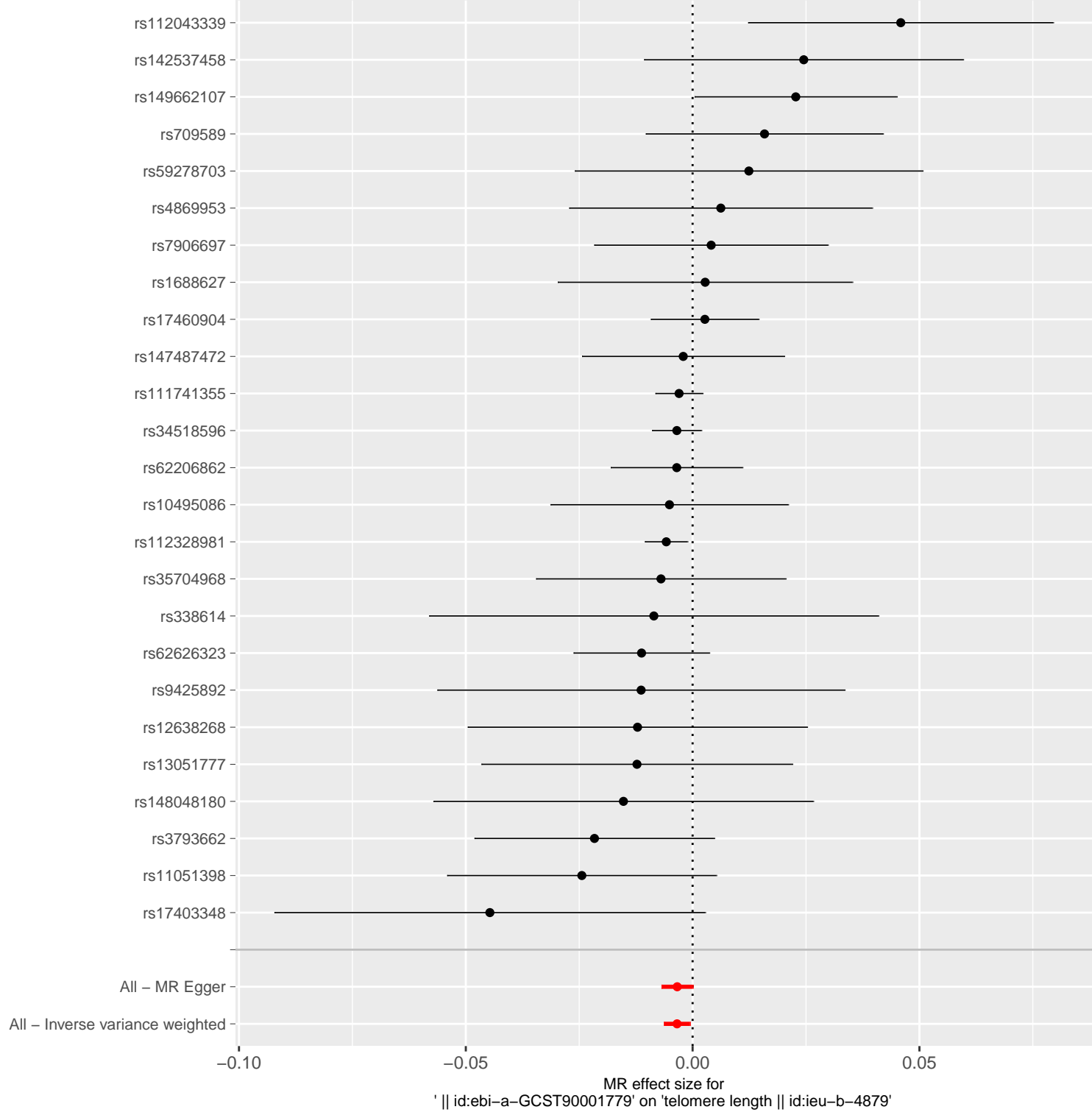

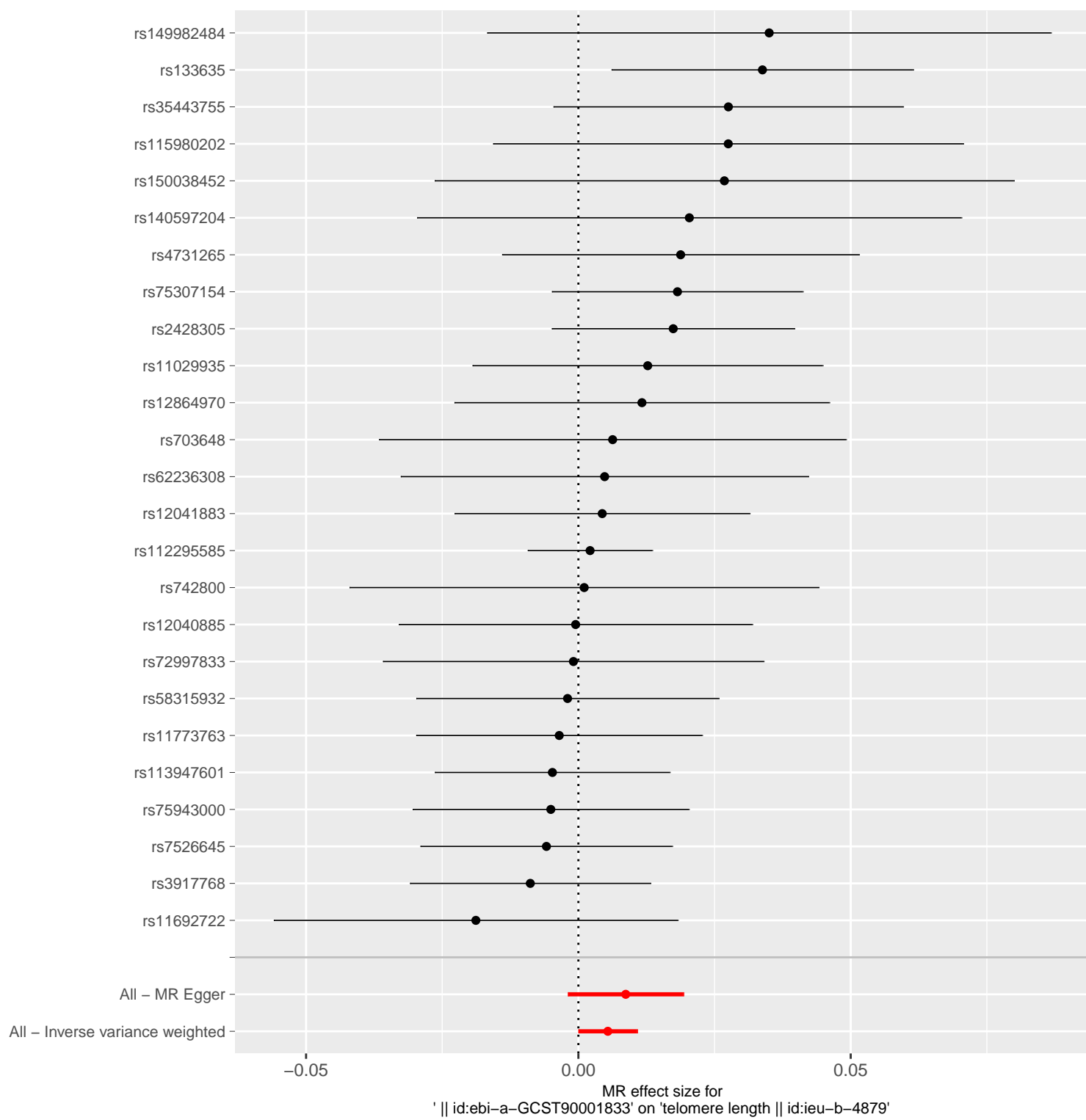

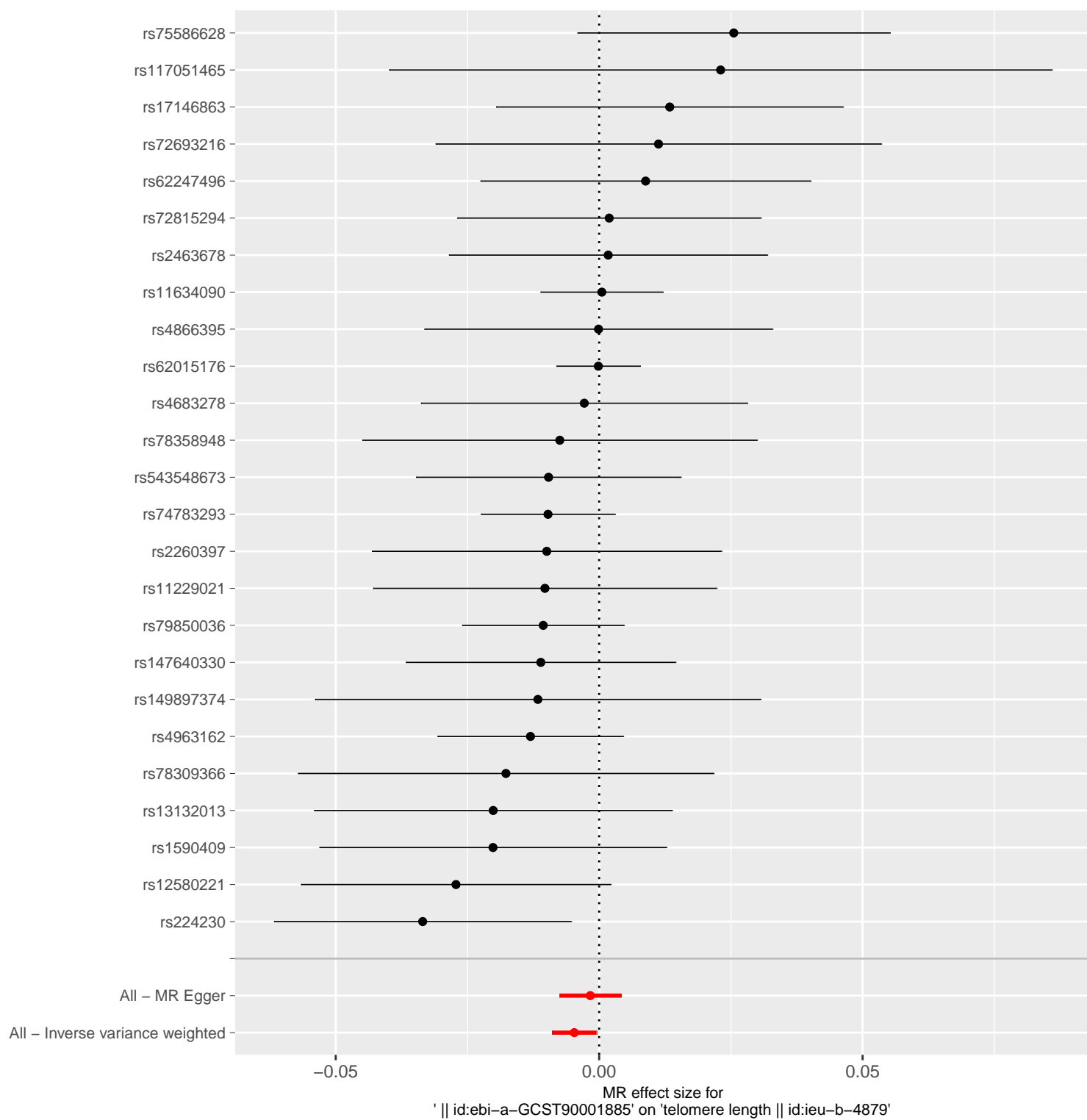

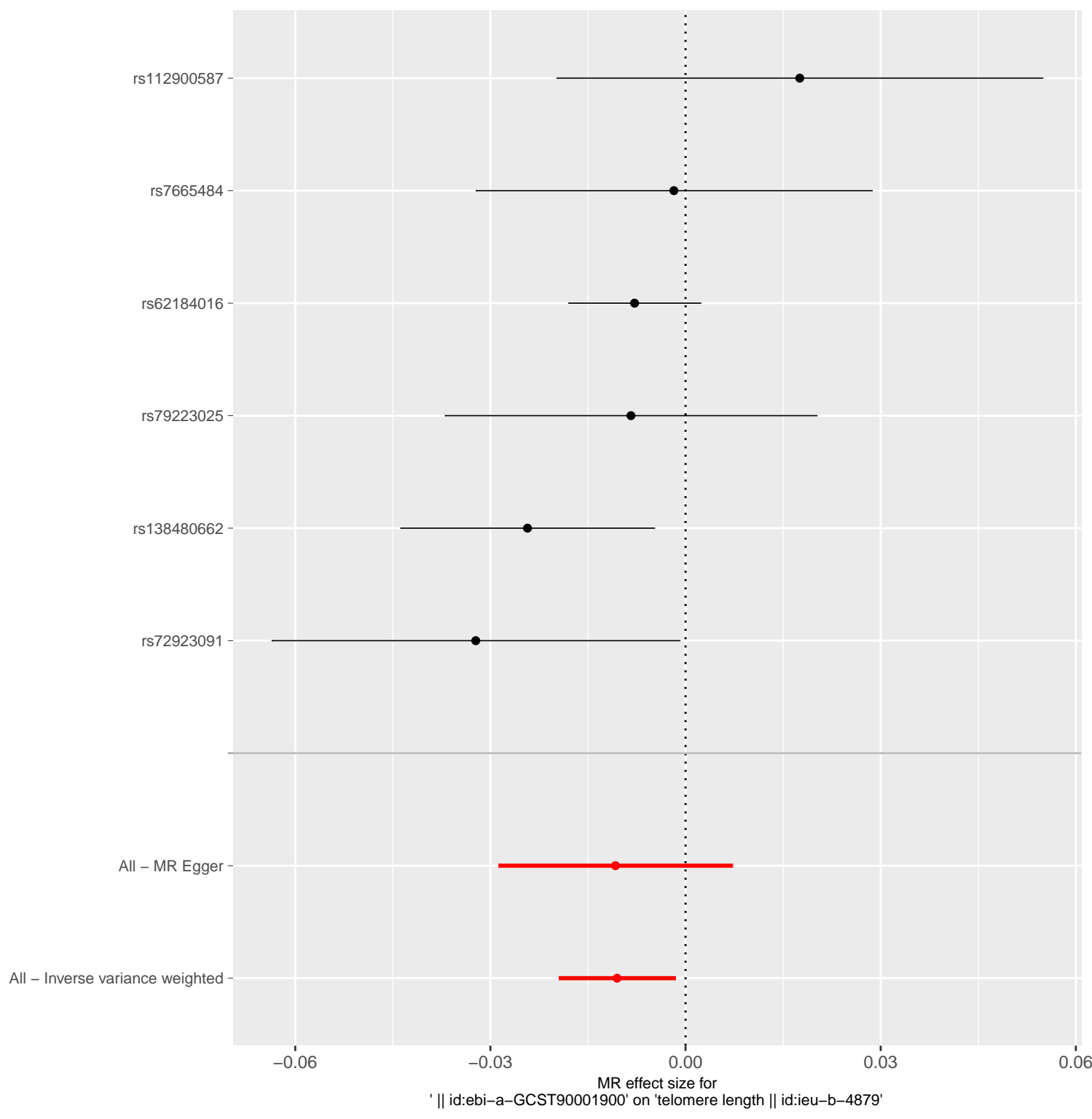

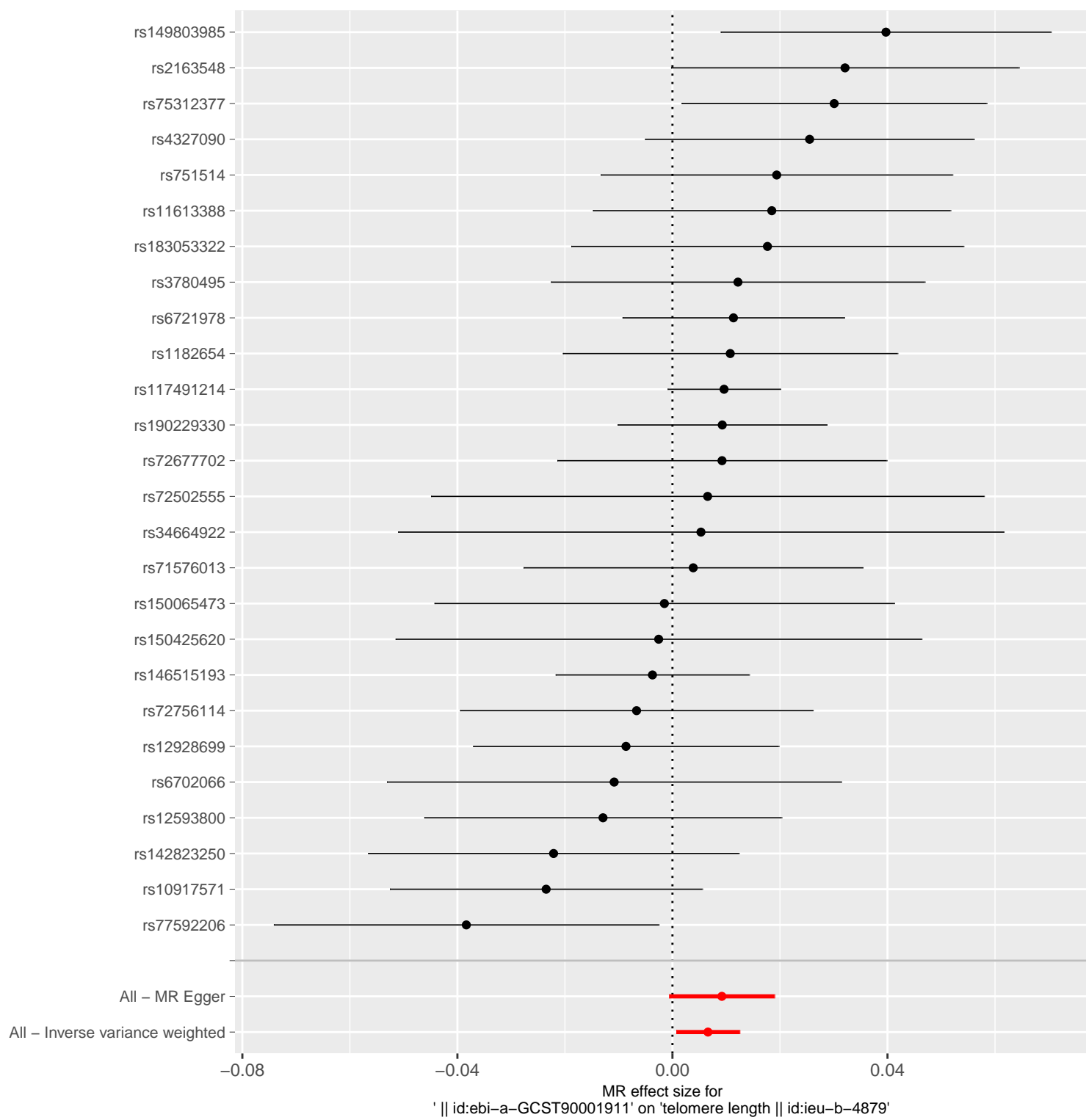

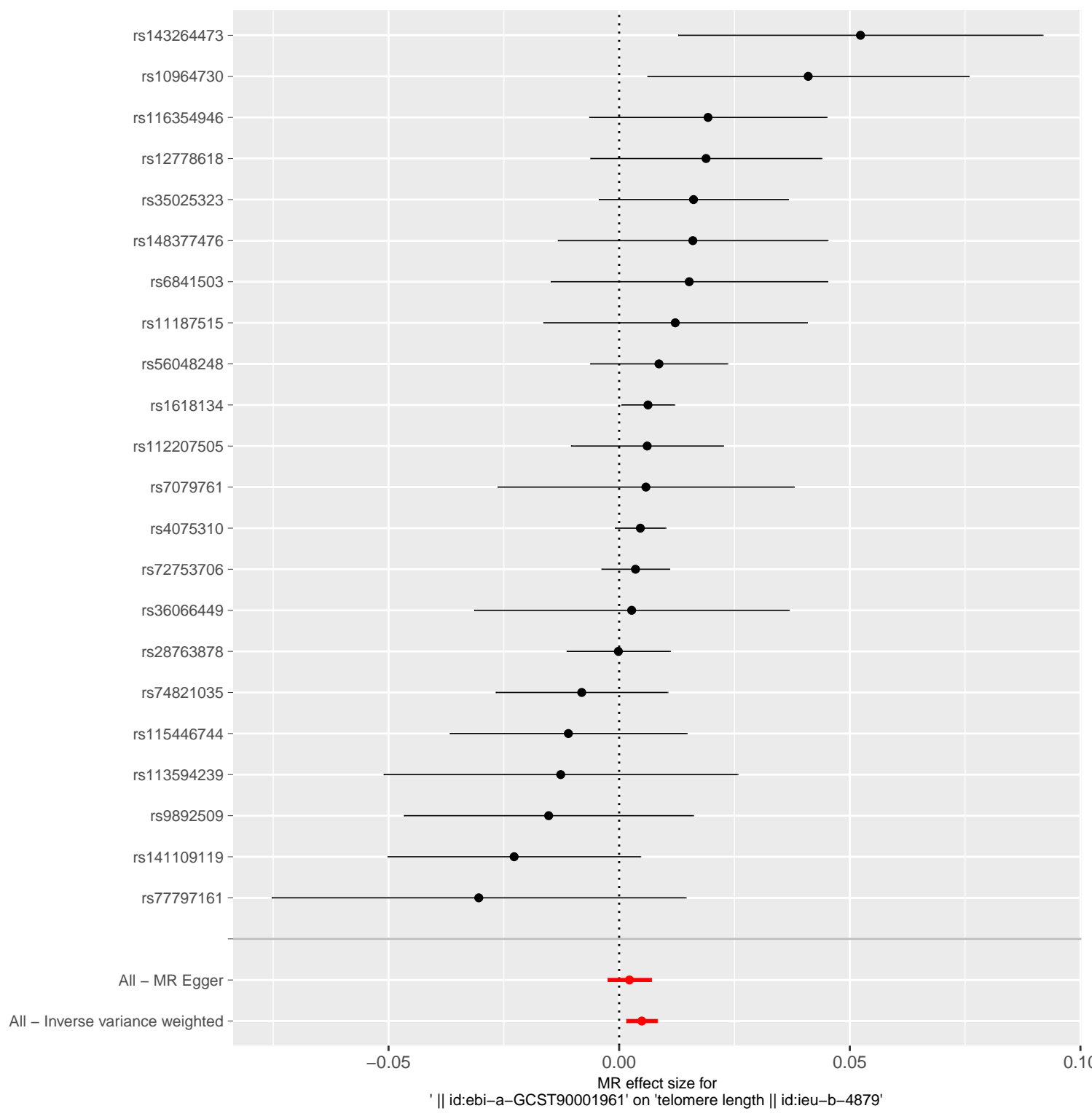

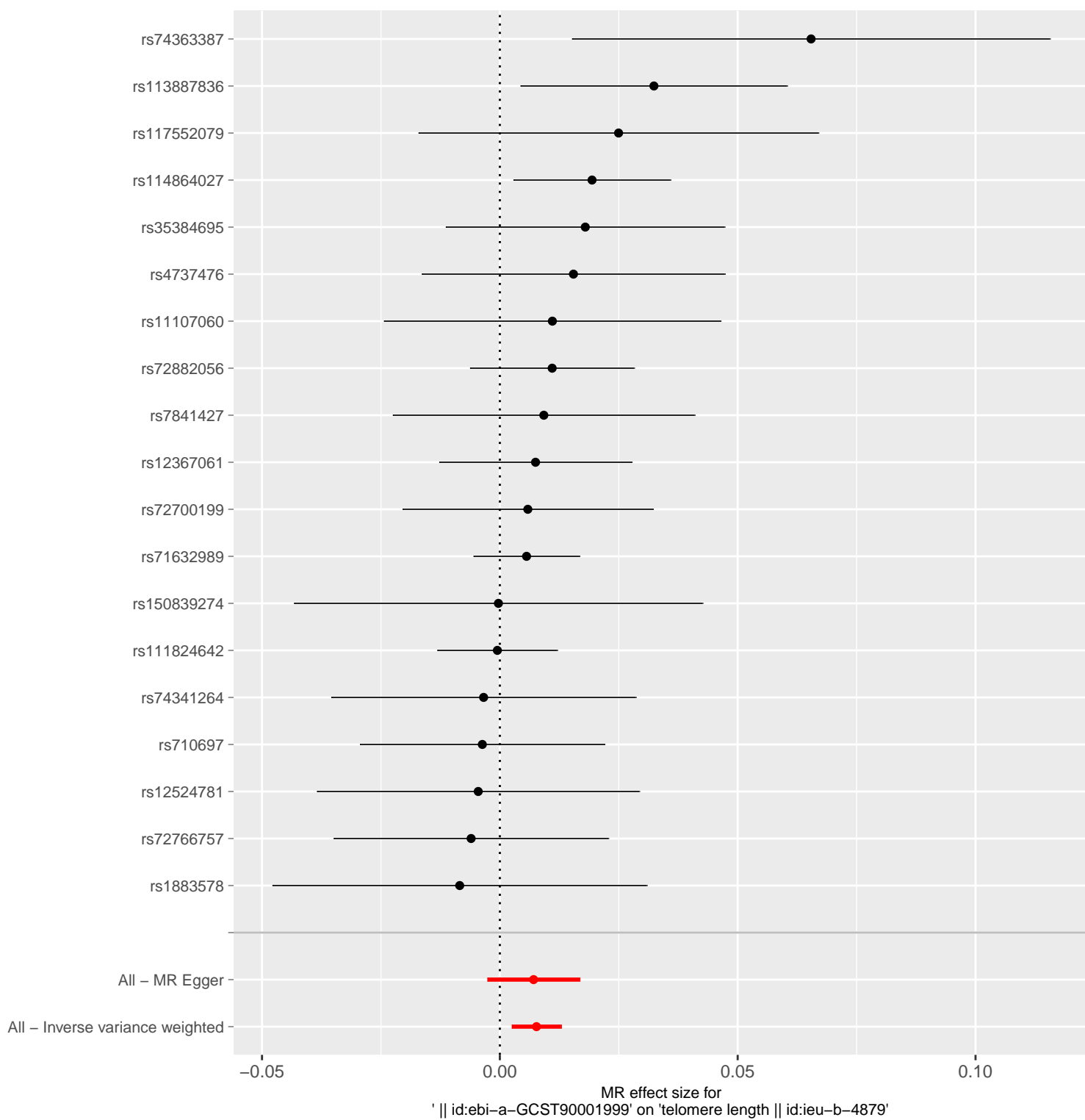

Supplement: Supplementary file 1 — Supplementary Material 1 [file 12865_2024_610_MOESM1_ESM.pdf]

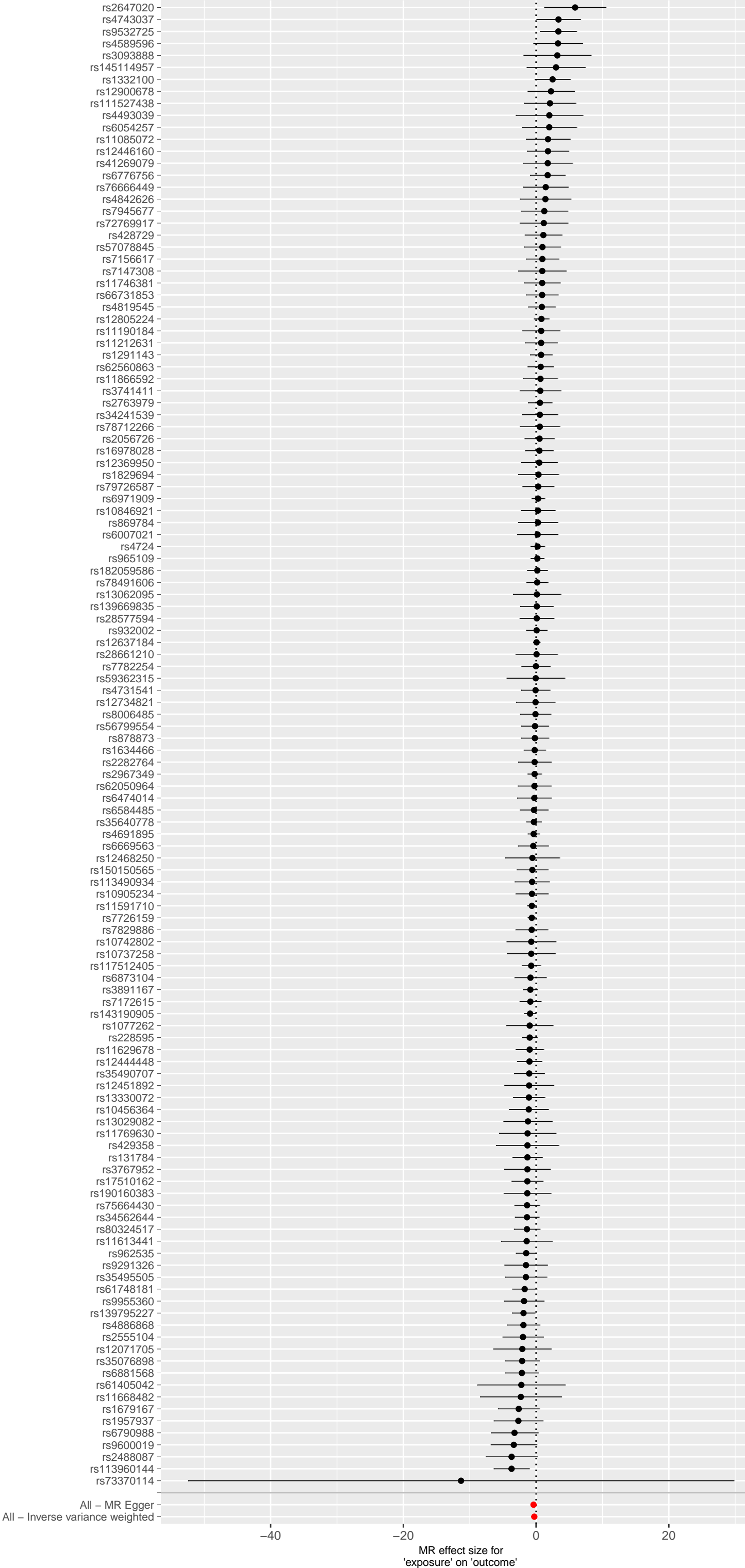

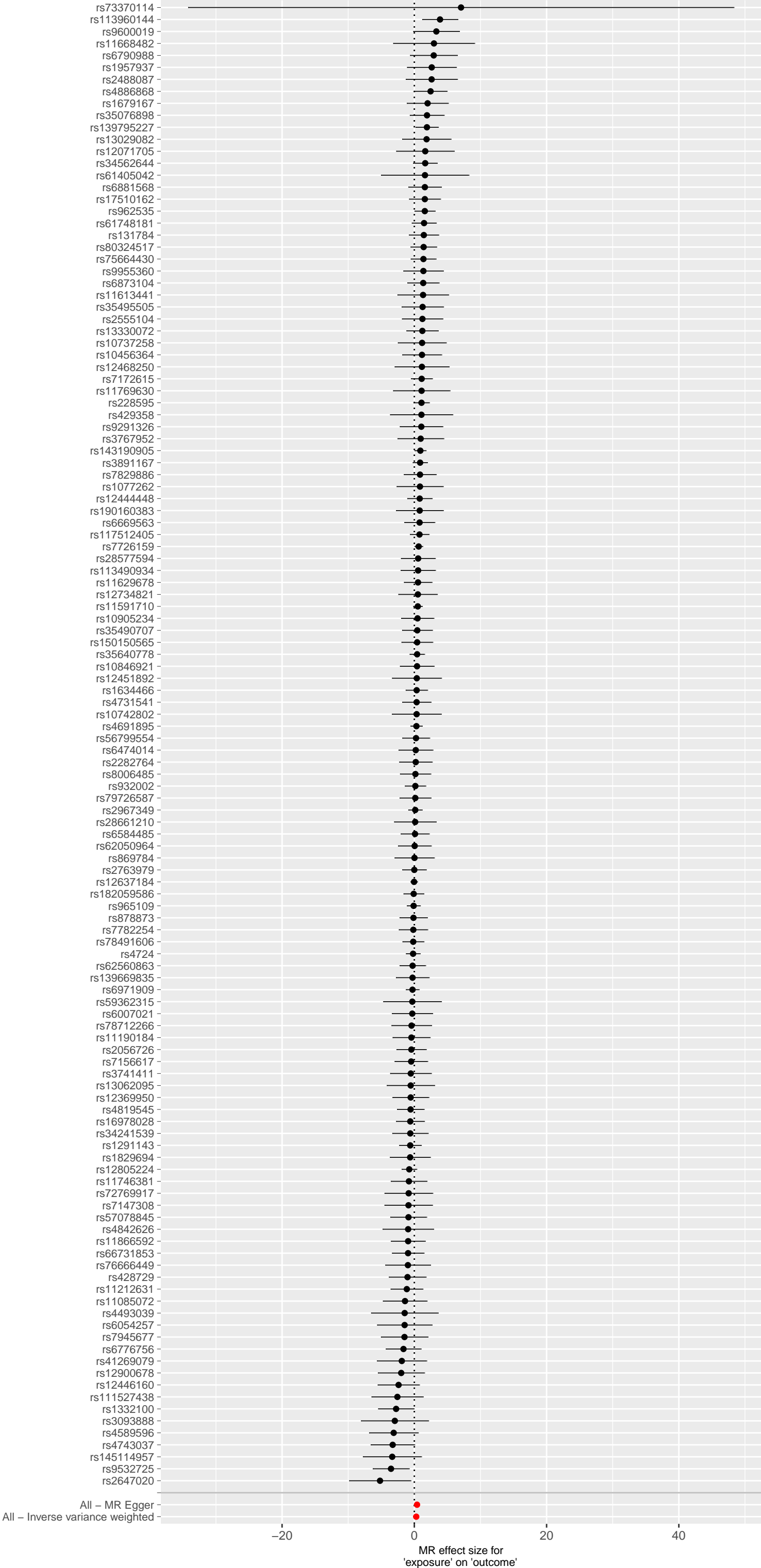

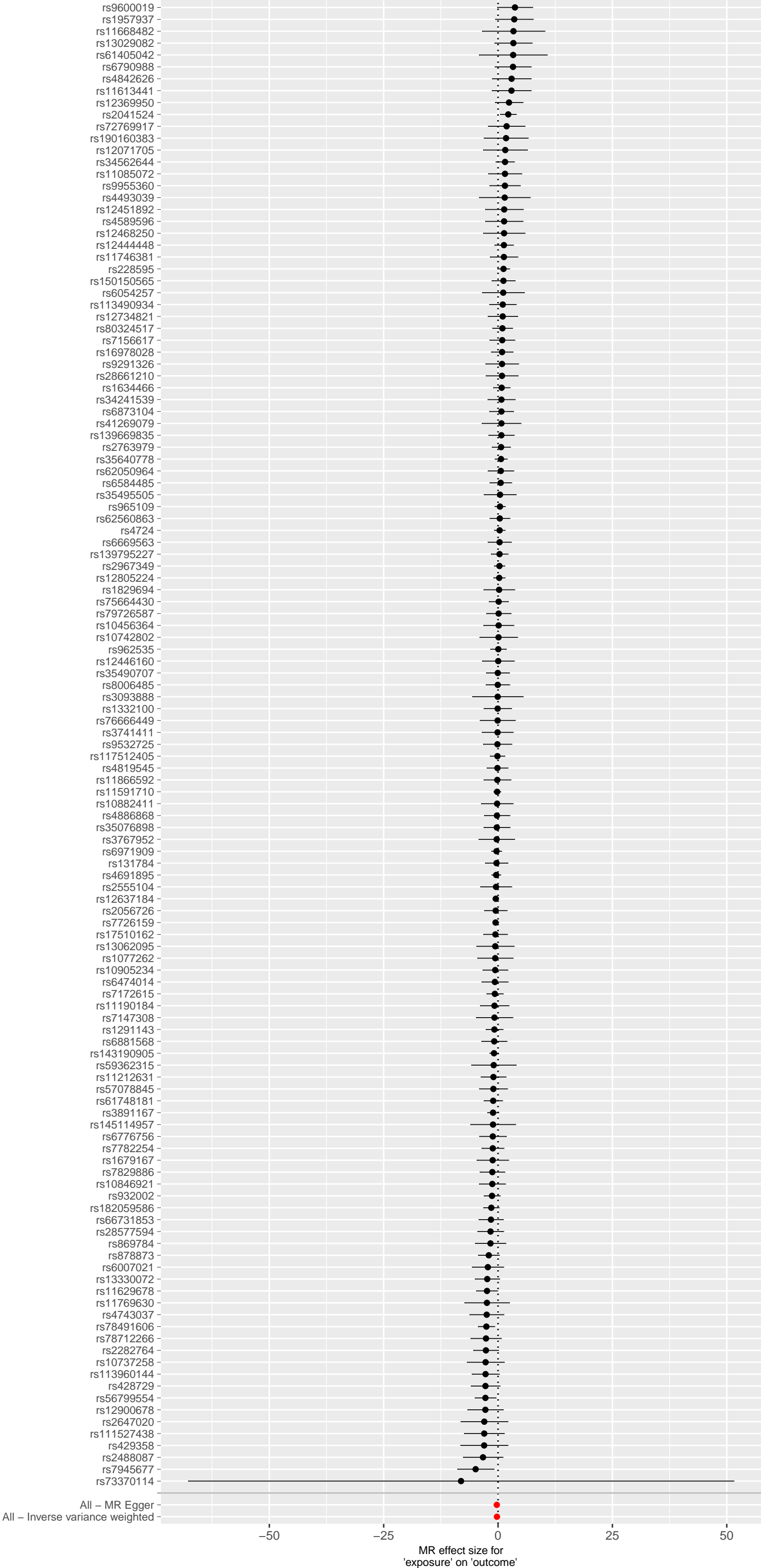

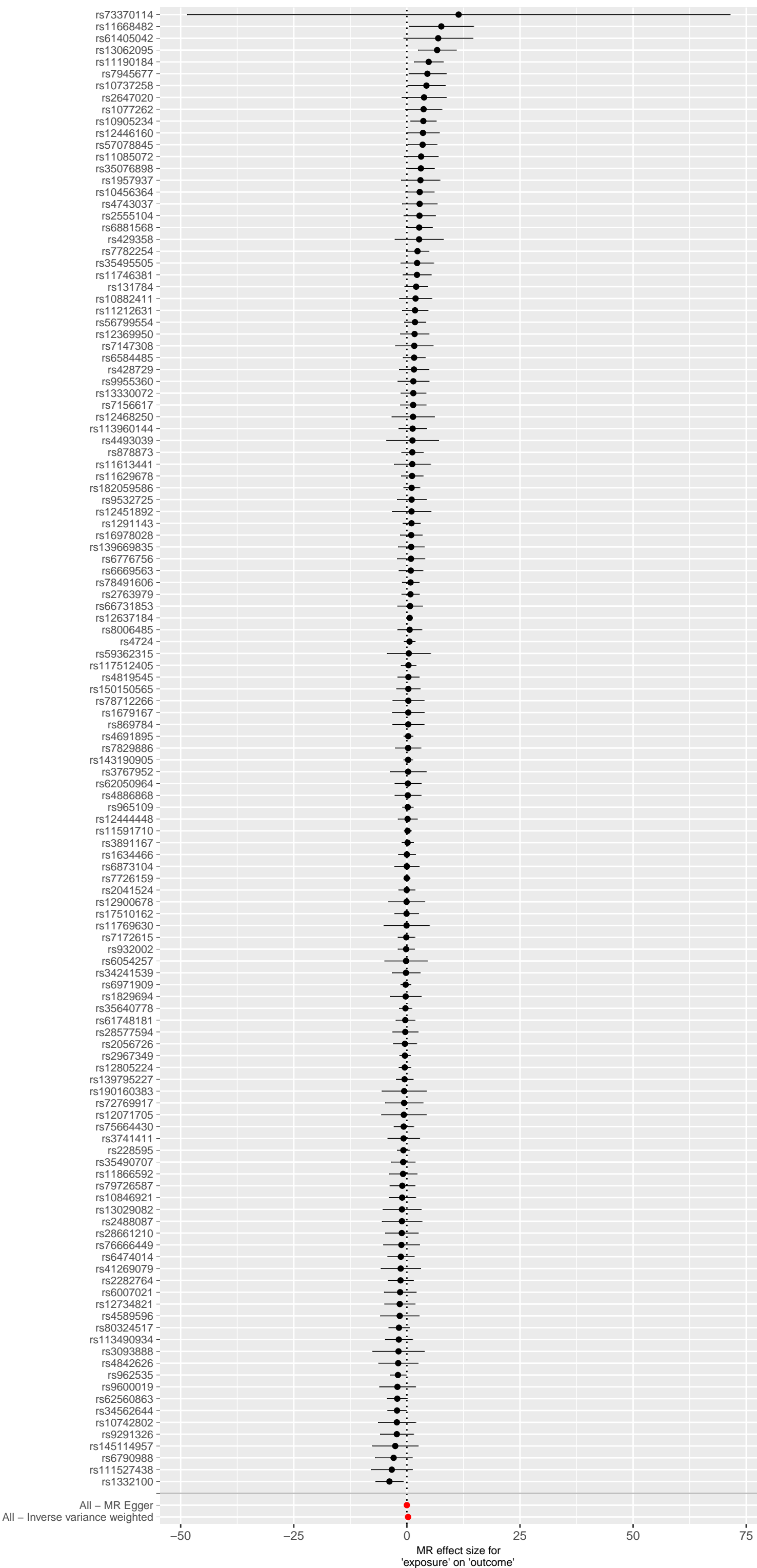

Supplement: Supplementary file 3 — Supplementary Material 3 [file 12865_2024_610_MOESM3_ESM.pdf]
