# Supplementary material for: Causal relationship between immune cells and telomere length: mendelian randomization analysis
Source: BMC Immunol. 2024 Mar 8;25:19. doi: 10.1186/s12865-024-00610-6 (PMC10924351; doi:10.1186/s12865-024-00610-6)
Supplement: Supplementary file 2 — Supplementary Material 2 [file 12865_2024_610_MOESM2_ESM.pdf]

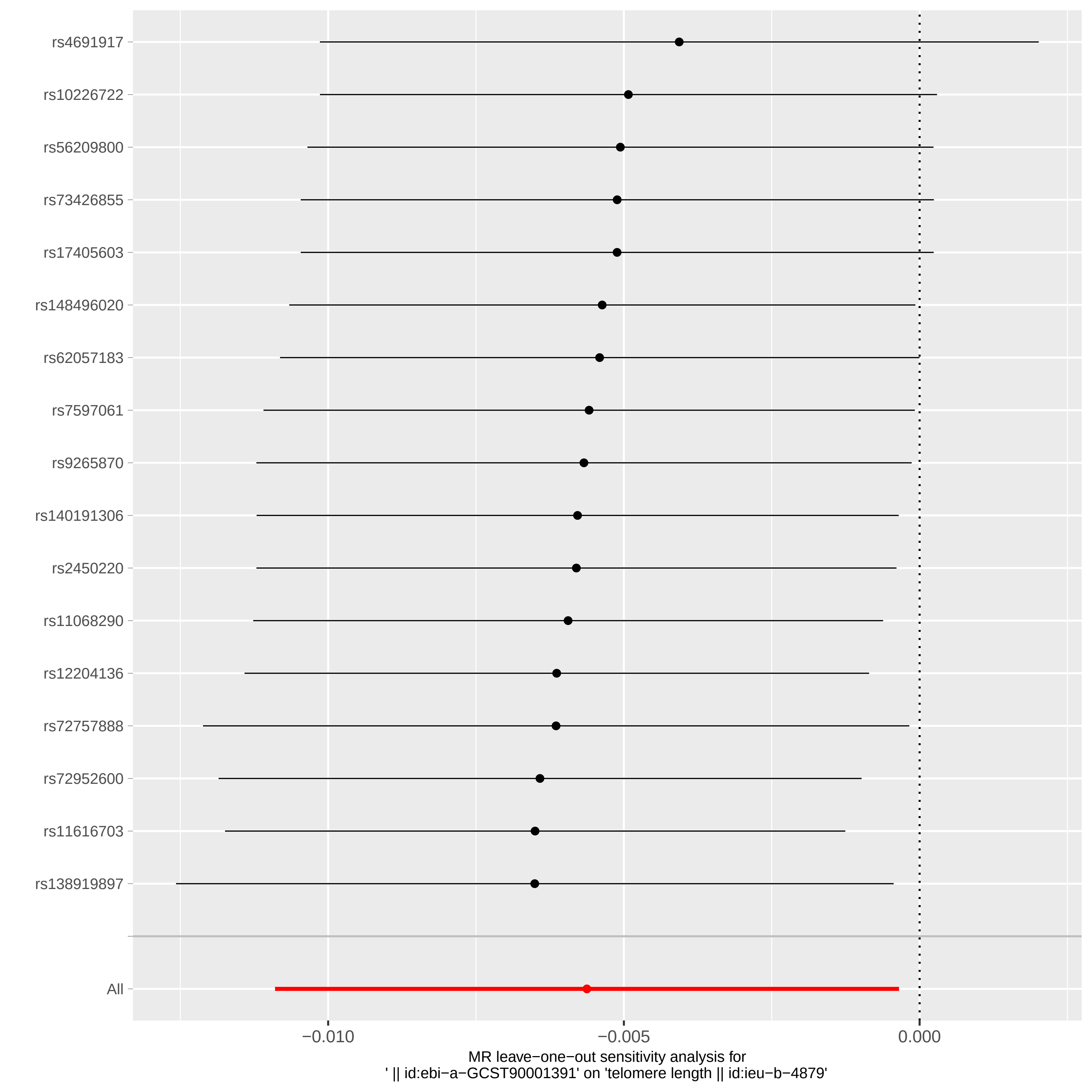
rs4691917
rs10226722
rs56209800
rs73426855
rs17405603
rs148496020
rs62057183
rs7597061
rs9265870
rs140191306
rs2450220
rs11068290
rs12204136
rs72757888
rs72952600
rs11616703
rs138919897
All
−0.010
−0.005
0.000
MR leave−one−out sensitivity analysis for
' || id:ebi−a−GCST90001391' on 'telomere length || id:ieu−b−4879'

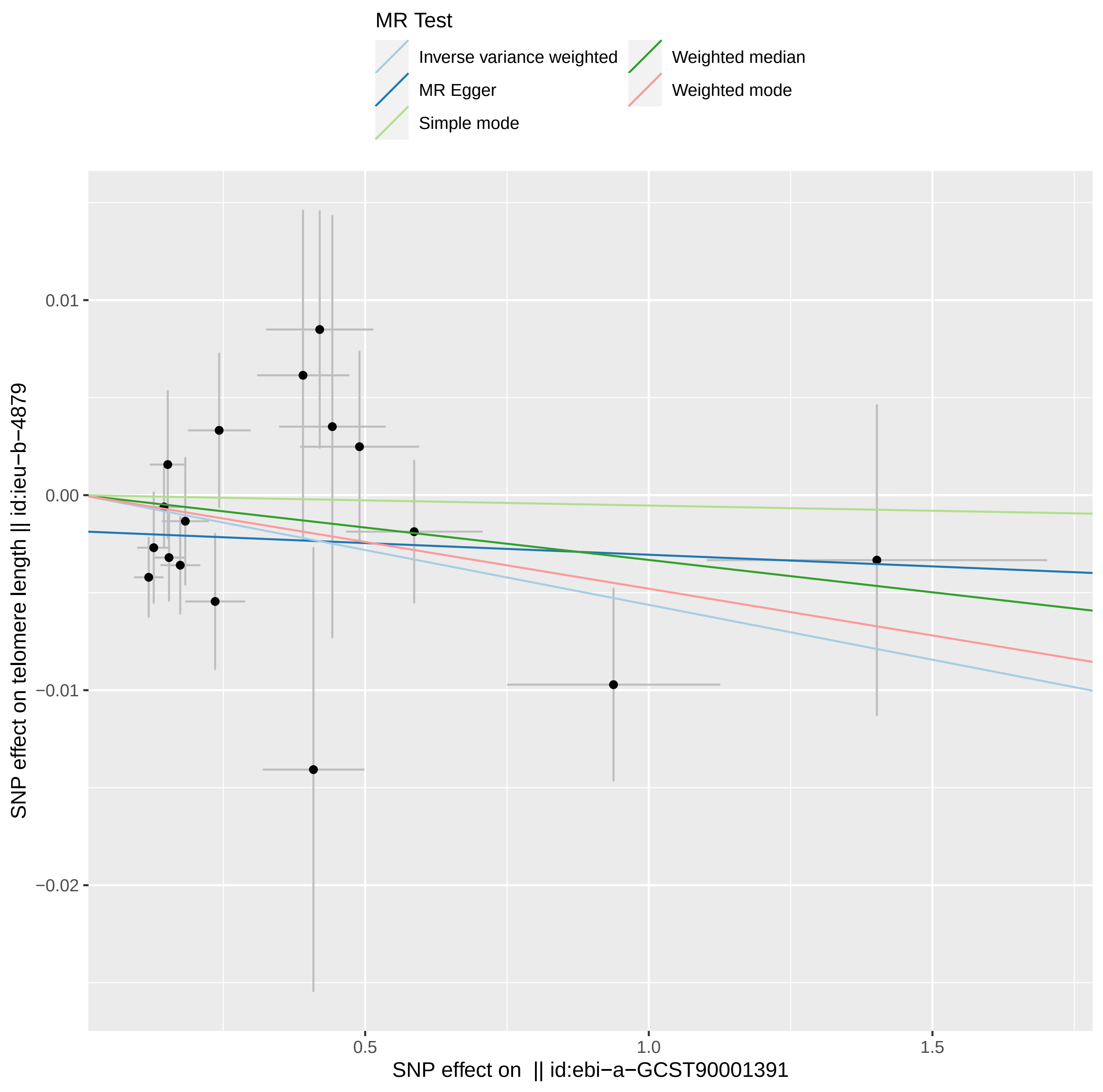
MR Test
Inverse variance weighted
MR Egger
Simple mode
Weighted median
Weighted mode
0.01
0.00
−0.01
−0.02
0.5
1.0
1.5
SNP effect on telomere length || id:ieu−b−4879
SNP effect on || id:ebi−a−GCST90001391

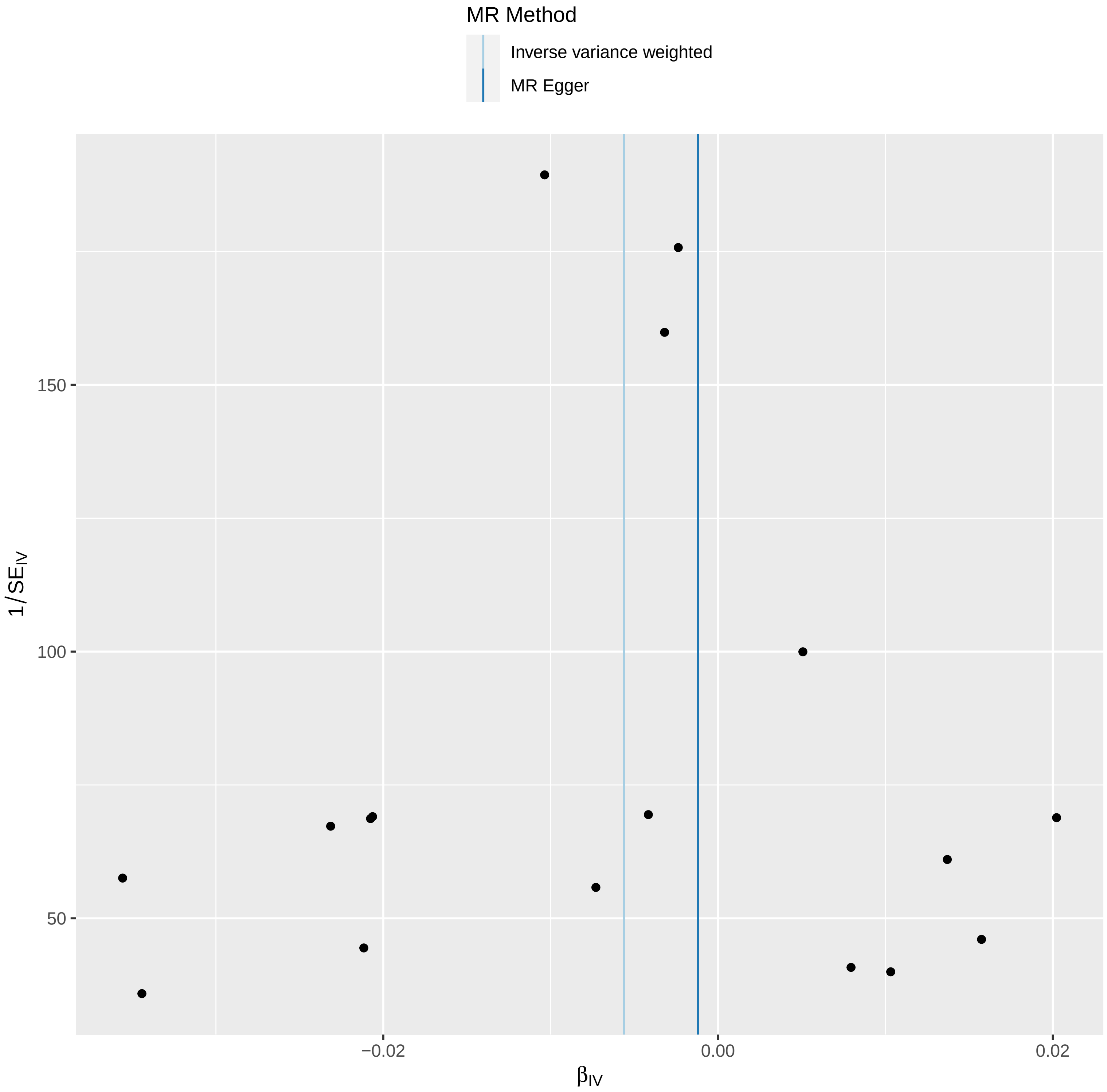
MR Method
Inverse variance weighted
MR Egger
150
100
50
1/SE_IV
−0.02
0.00
0.02
β_IV

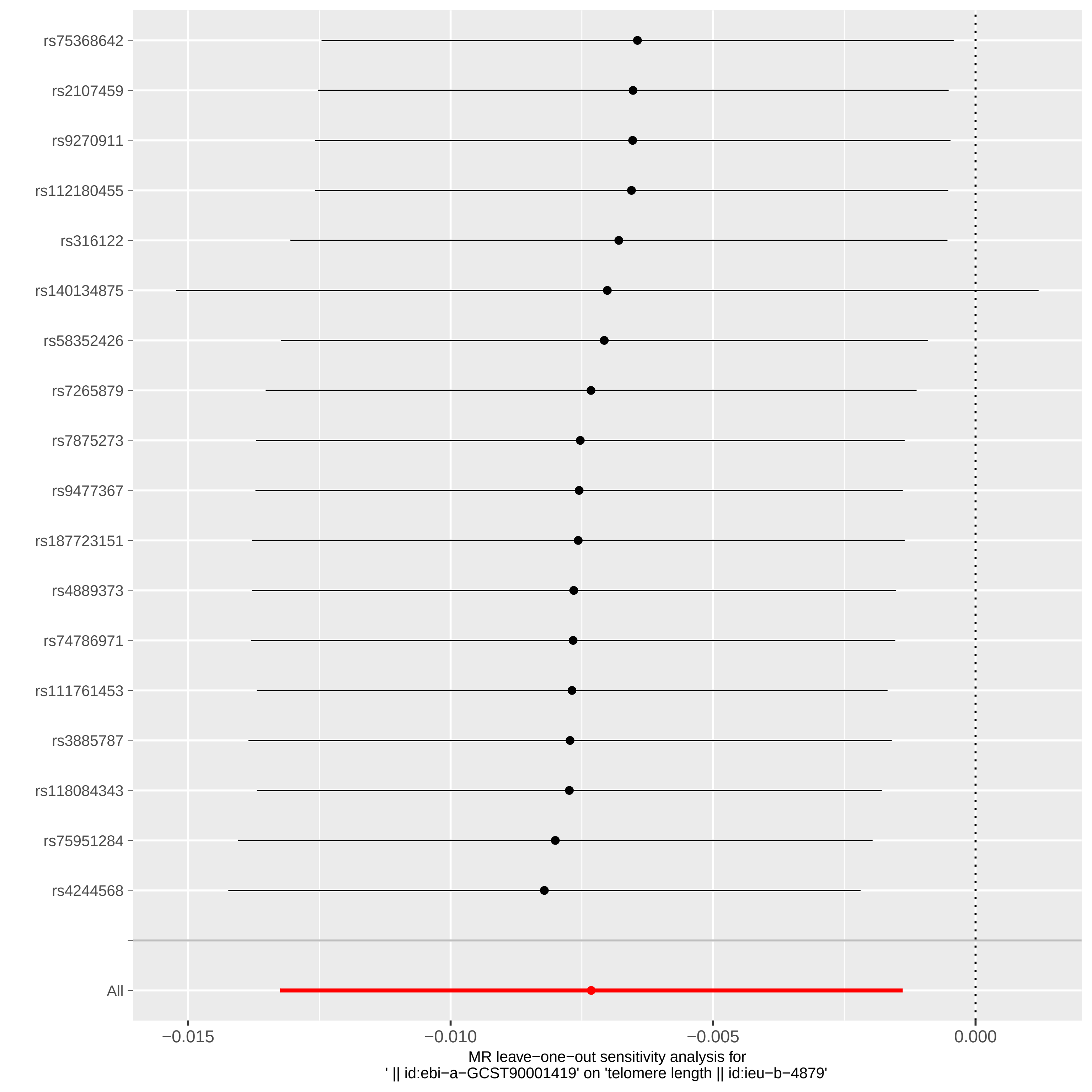
rs75368642
rs2107459
rs9270911
rs112180455
rs316122
rs140134875
rs58352426
rs7265879
rs7875273
rs9477367
rs187723151
rs4889373
rs74786971
rs111761453
rs3885787
rs118084343
rs75951284
rs4244568
All
−0.015
−0.010
−0.005
0.000
MR leave−one−out sensitivity analysis for
' || id:ebi−a−GCST90001419' on 'telomere length || id:ieu−b−4879'

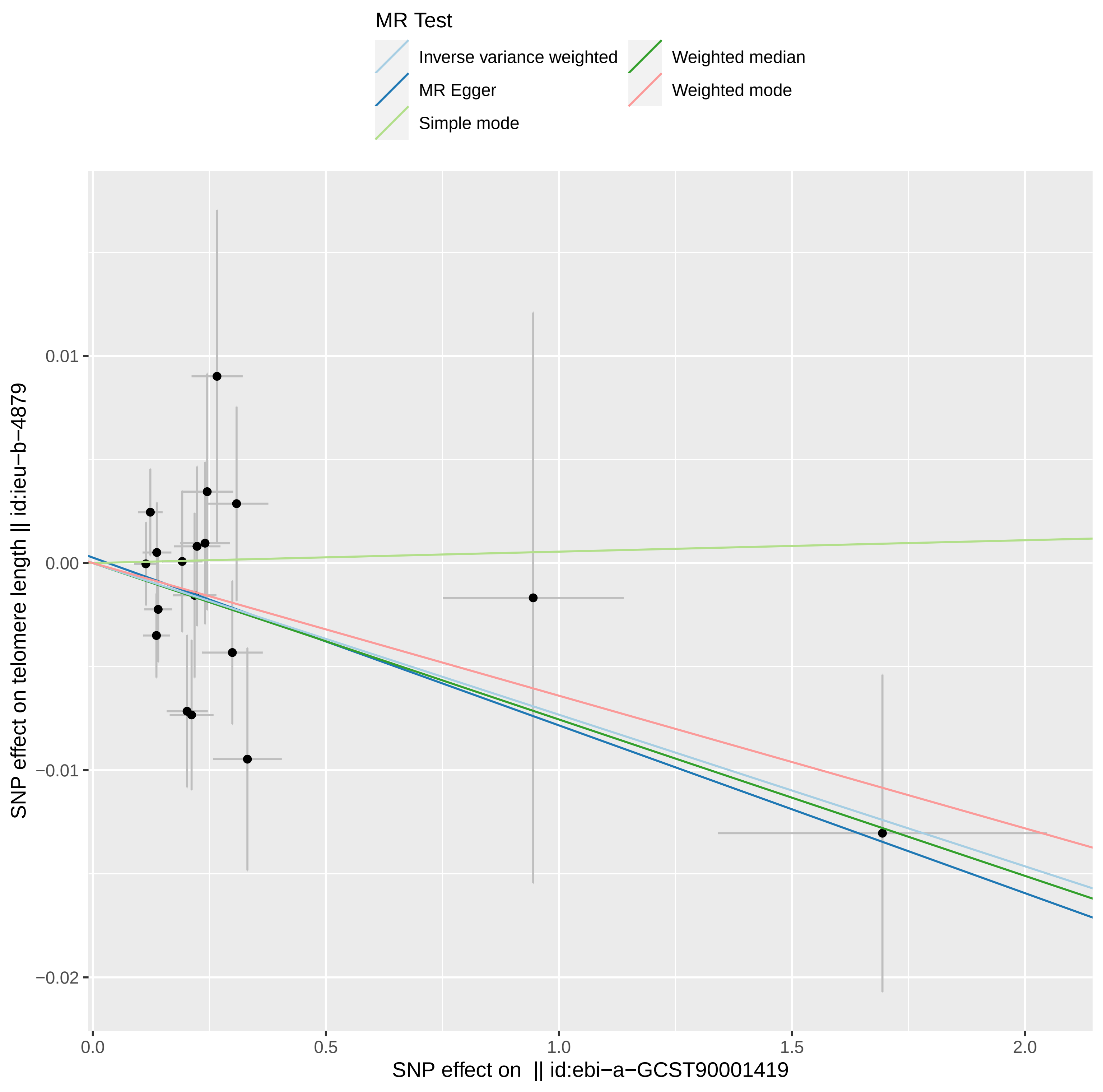
MR Test
Inverse variance weighted
MR Egger
Simple mode
Weighted median
Weighted mode
0.01
0.00
−0.01
−0.02
SNP effect on telomere length || id:ieu−b−4879
0.0
0.5
1.0
1.5
2.0
SNP effect on || id:ebi−a−GCST90001419

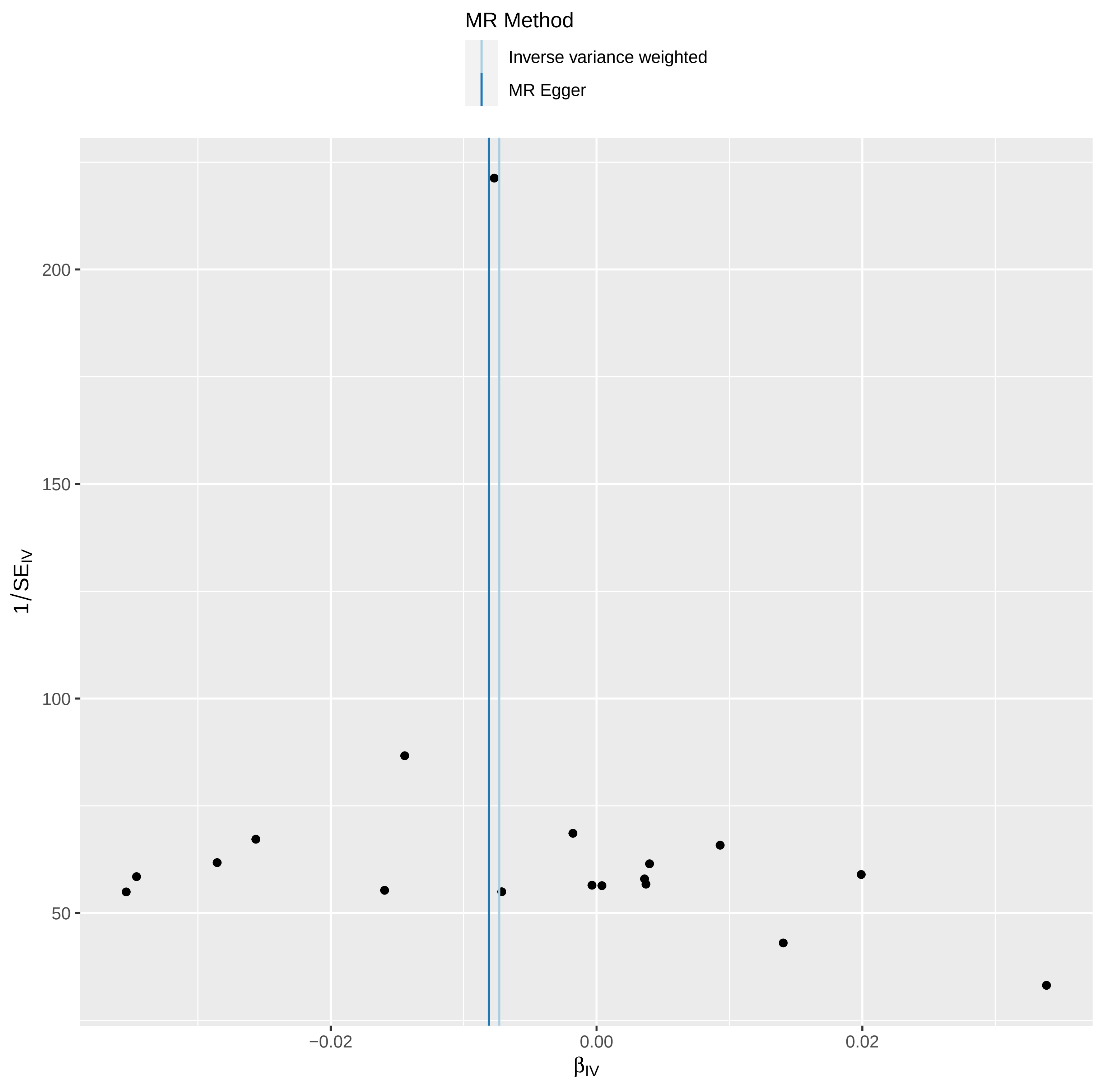

MR Method
Inverse variance weighted
MR Egger
200
150
100
50
1/SE_IV
−0.02
0.00
0.02
β_IV

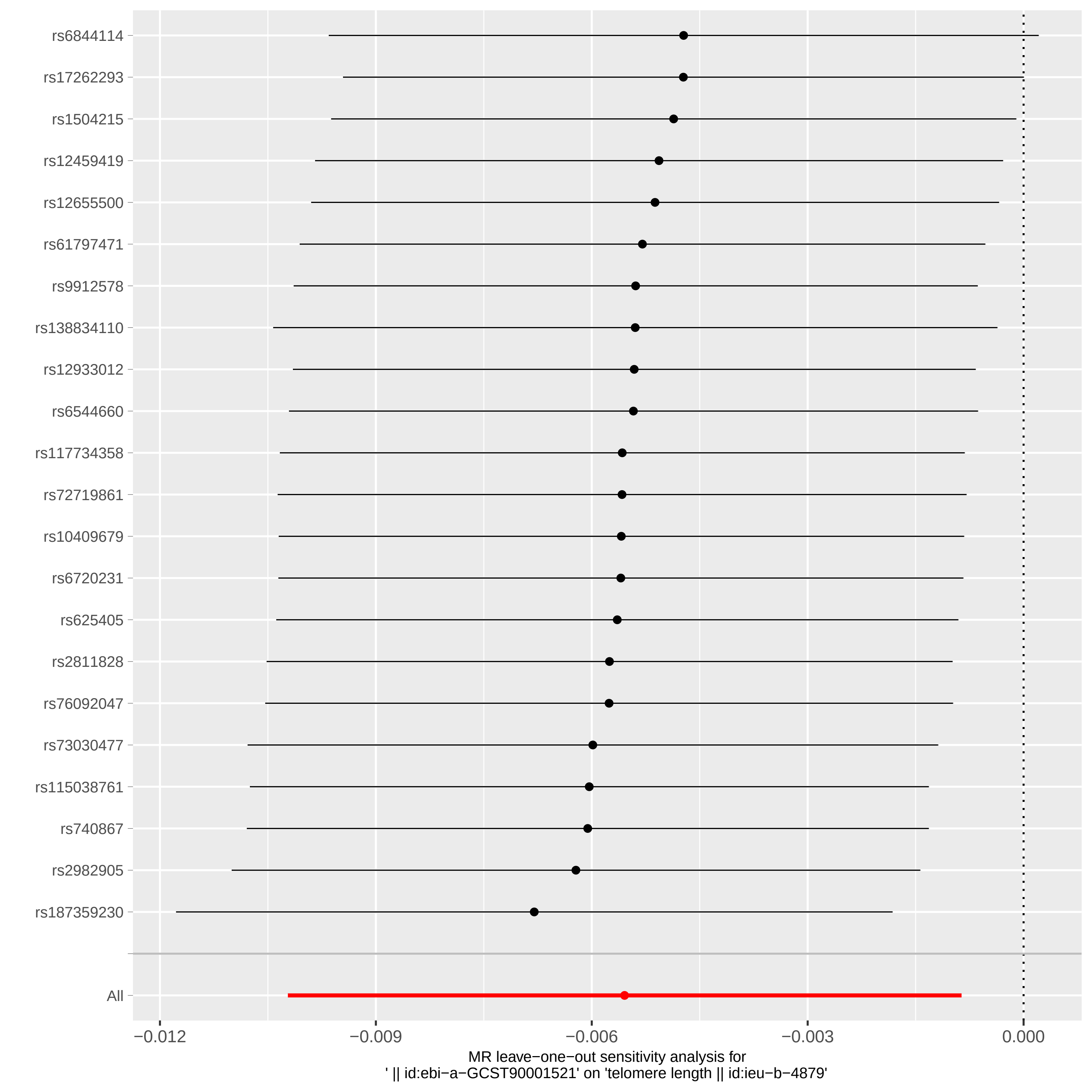
rs6844114
rs17262293
rs1504215
rs12459419
rs12655500
rs61797471
rs9912578
rs138834110
rs12933012
rs6544660
rs117734358
rs72719861
rs10409679
rs6720231
rs625405
rs2811828
rs76092047
rs73030477
rs115038761
rs740867
rs2982905
rs187359230
All
−0.012
−0.009
−0.006
−0.003
0.000
MR leave−one−out sensitivity analysis for
' || id:ebi−a−GCST90001521' on 'telomere length || id:ieu−b−4879'

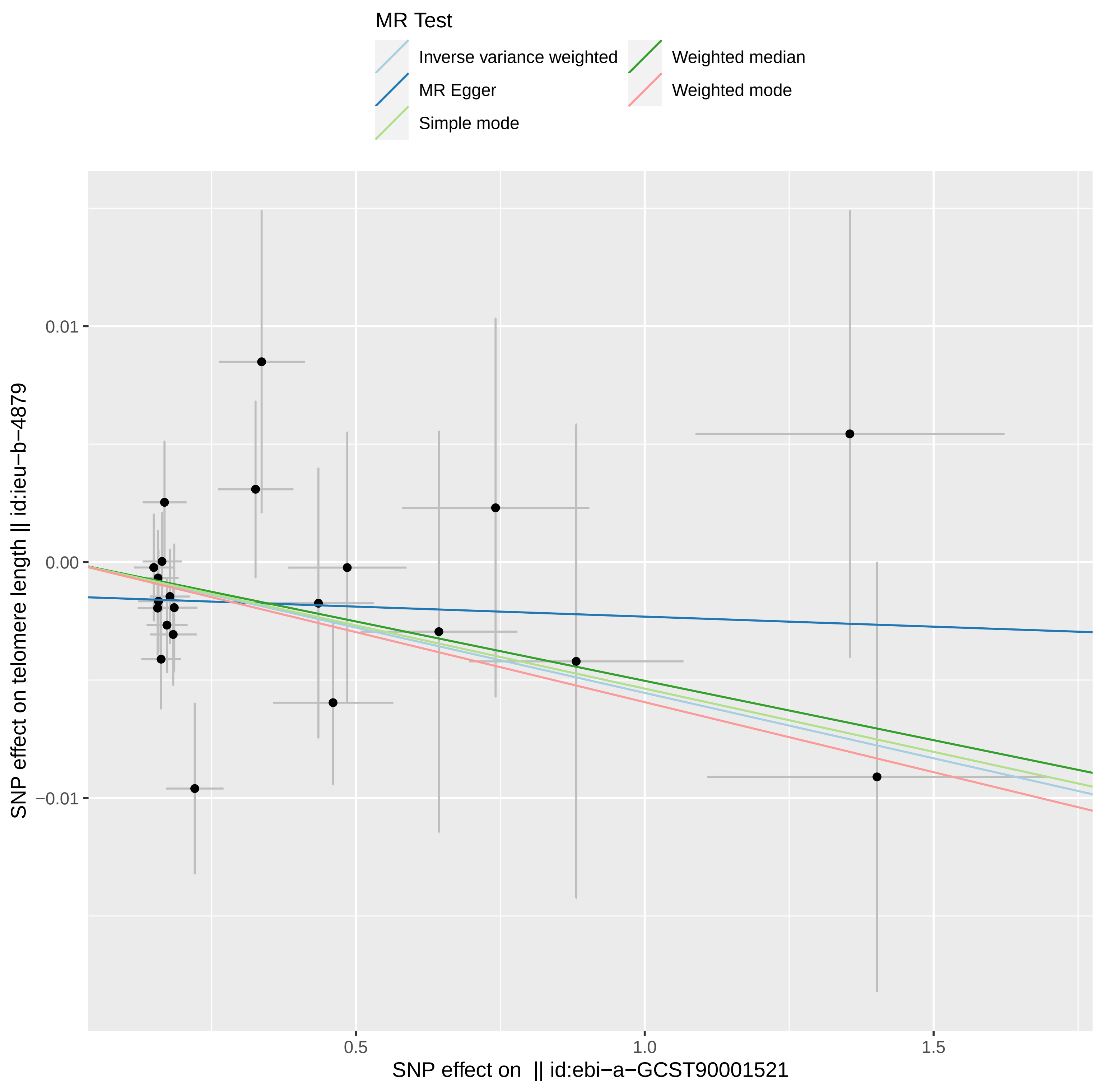
MR Test
Inverse variance weighted
MR Egger
Simple mode
Weighted median
Weighted mode
SNP effect on telomere length || id:ieu-b-4879
0.01
0.00
−0.01
0.5
1.0
1.5
SNP effect on || id:ebi−a−GCST90001521

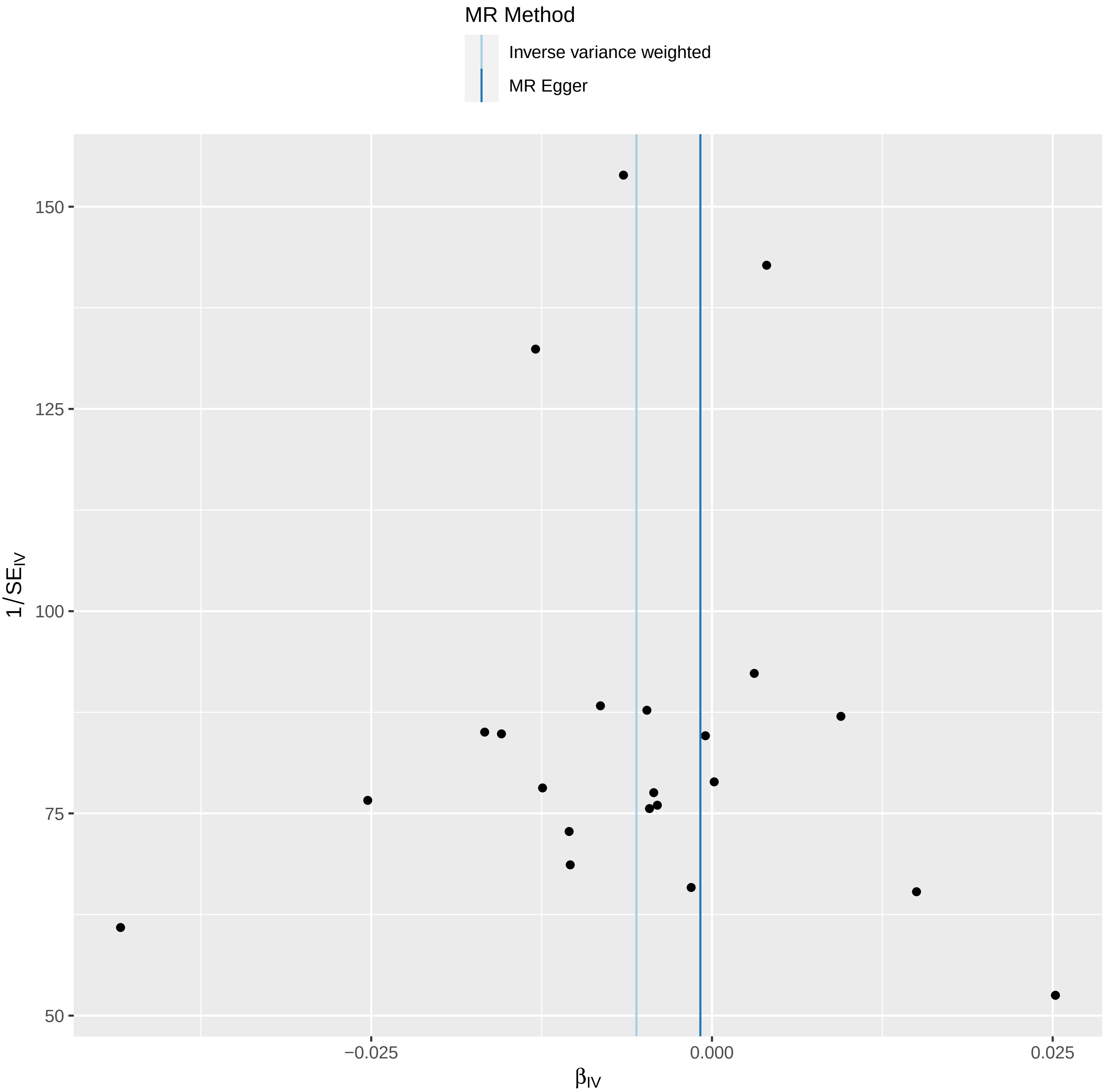
MR Method
Inverse variance weighted
MR Egger
150
125
100
75
50
1/SE$_{IV}$
−0.025
0.000
0.025
β$_{IV}$

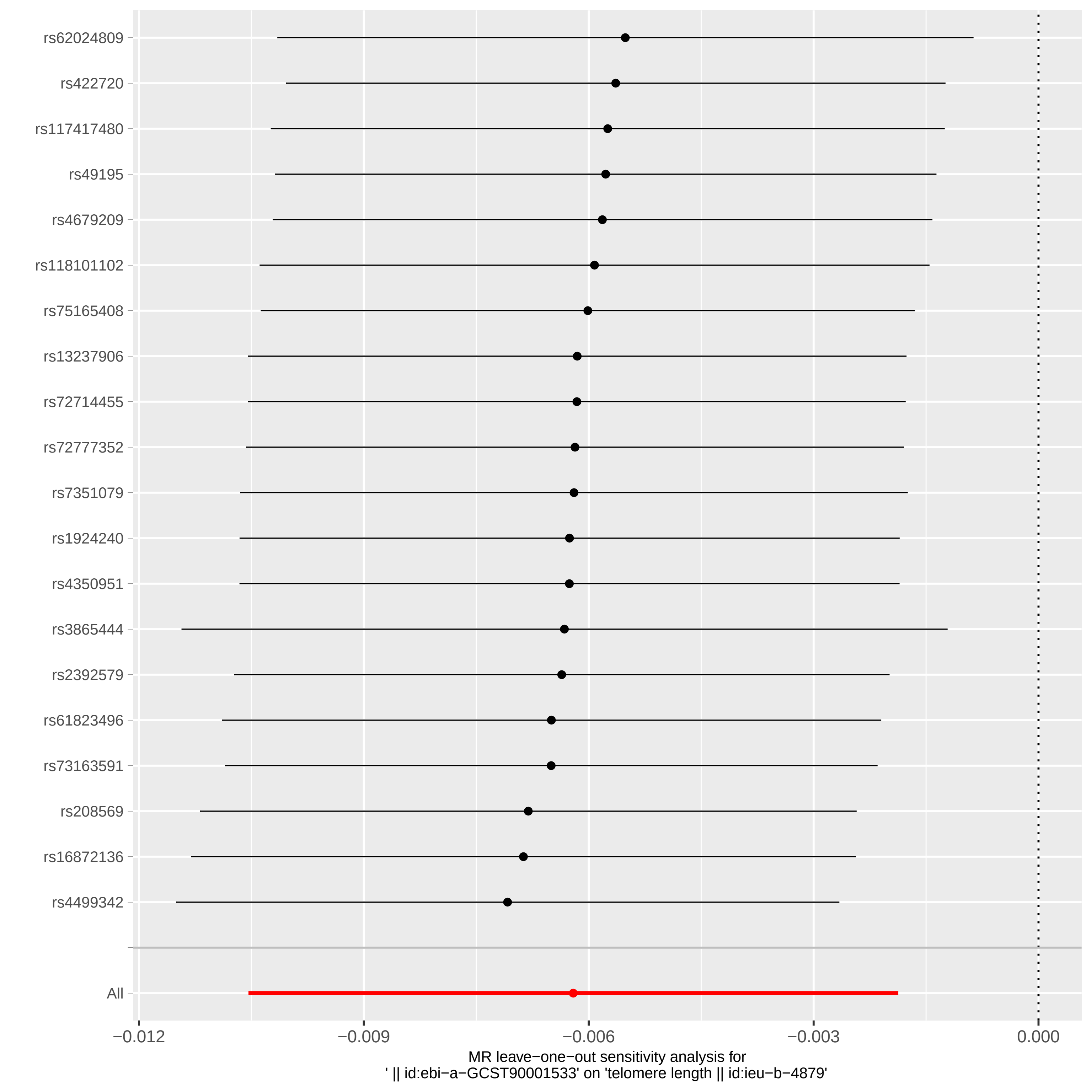

rs62024809
rs422720
rs117417480
rs49195
rs4679209
rs118101102
rs75165408
rs13237906
rs72714455
rs72777352
rs7351079
rs1924240
rs4350951
rs3865444
rs2392579
rs61823496
rs73163591
rs208569
rs16872136
rs4499342
All
−0.012
−0.009
−0.006
−0.003
0.000
MR leave−one−out sensitivity analysis for
' || id:ebi−a−GCST90001533' on 'telomere length || id:ieu−b−4879'

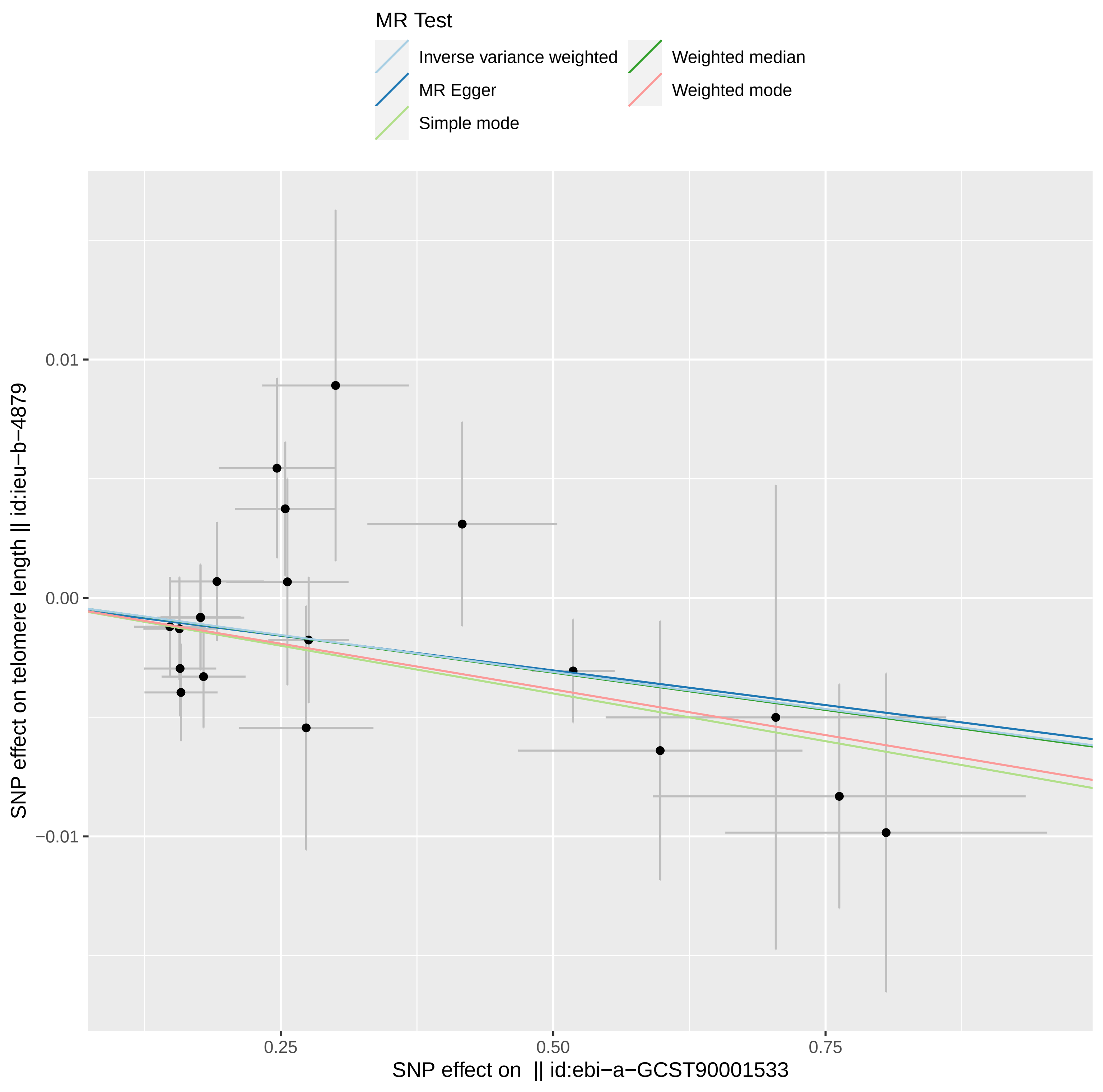
MR Test
Inverse variance weighted
MR Egger
Simple mode
Weighted median
Weighted mode
0.01
0.00
−0.01
SNP effect on telomere length || id:ieu−b−4879
0.25
0.50
0.75
SNP effect on || id:ebi−a−GCST90001533

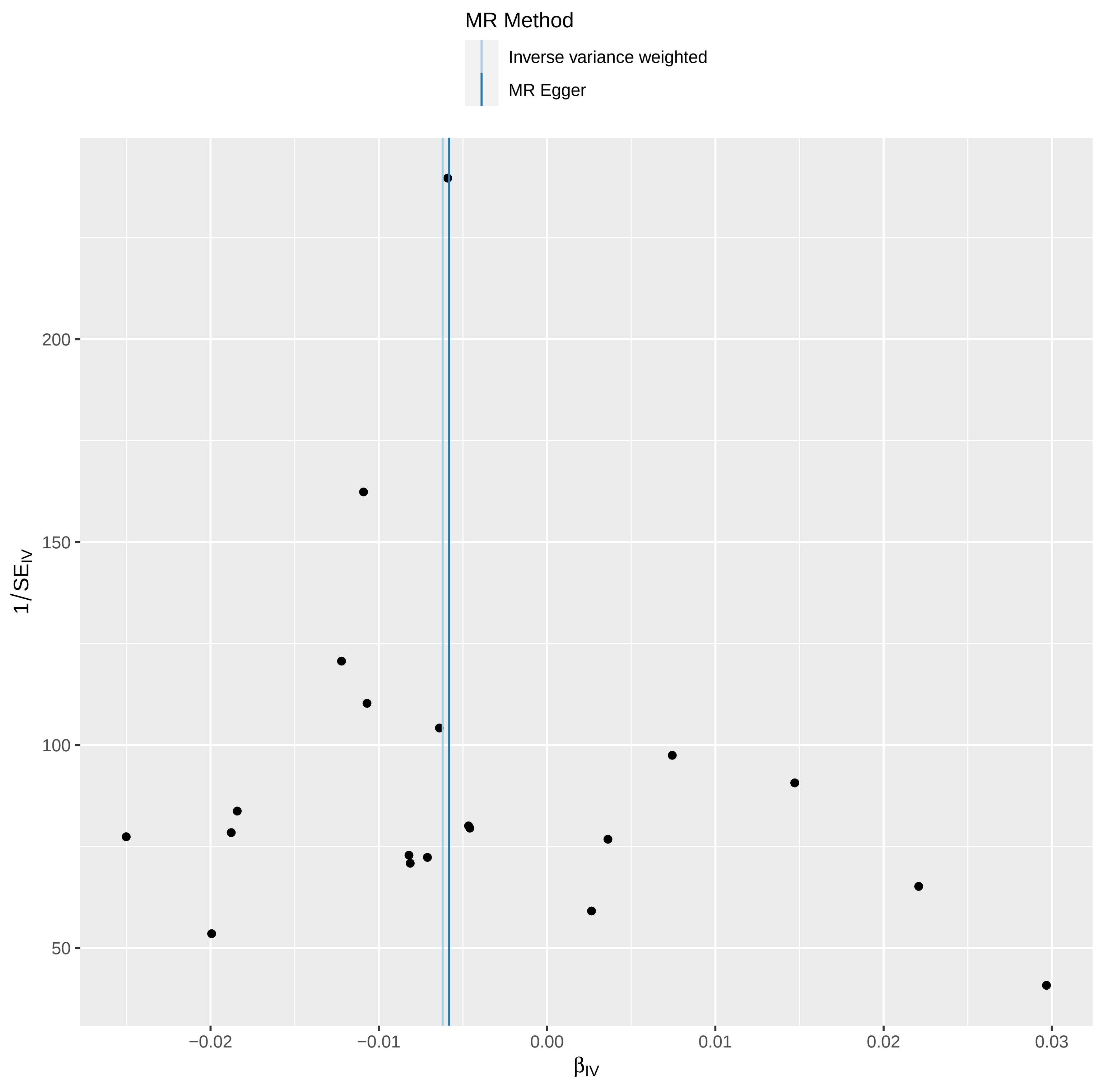

MR Method
Inverse variance weighted
MR Egger
200
150
100
50
1/SE$_{IV}$
−0.02
−0.01
0.00
0.01
0.02
0.03
β$_{IV}$

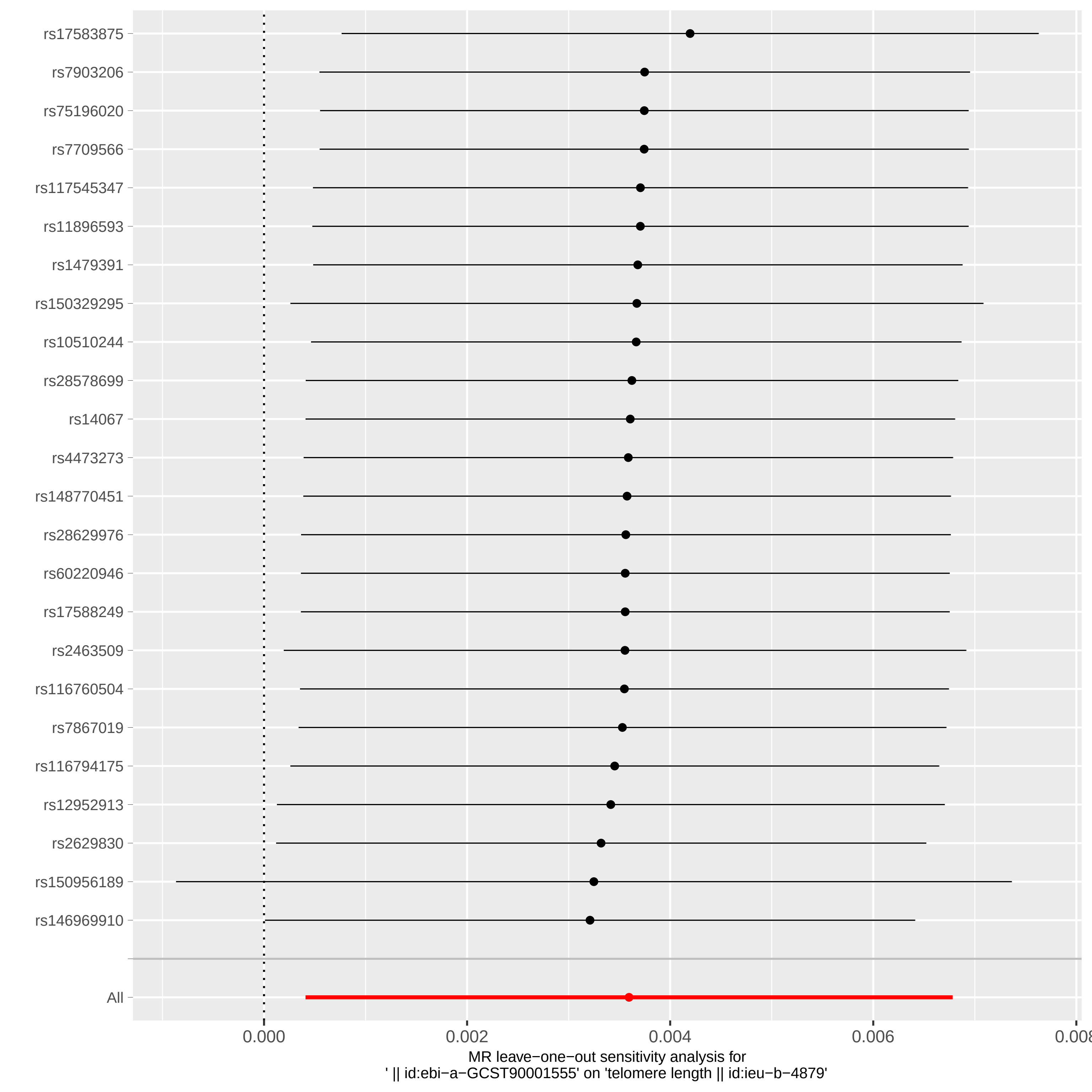
rs17583875
rs7903206
rs75196020
rs7709566
rs117545347
rs11896593
rs1479391
rs150329295
rs10510244
rs28578699
rs14067
rs4473273
rs148770451
rs28629976
rs60220946
rs17588249
rs2463509
rs116760504
rs7867019
rs116794175
rs12952913
rs2629830
rs150956189
rs146969910
All
0.000
0.002
0.004
0.006
0.008
MR leave−one−out sensitivity analysis for
' || id:ebi−a−GCST90001555' on 'telomere length || id:ieu−b−4879'

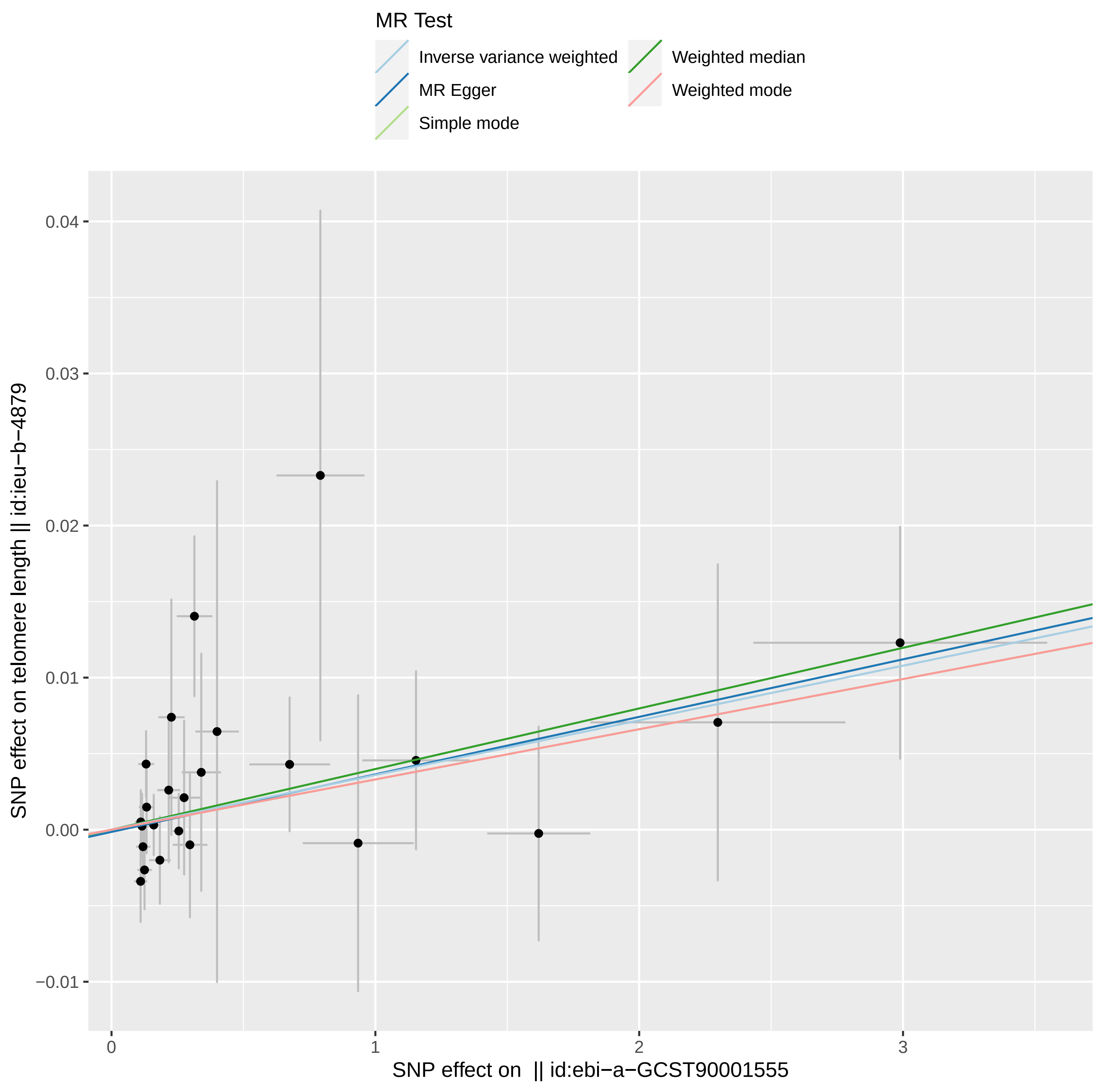
MR Test
Inverse variance weighted
MR Egger
Simple mode
Weighted median
Weighted mode
SNP effect on telomere length || id:ieu−b−4879
0.04
0.03
0.02
0.01
0.00
−0.01
0
1
2
3
SNP effect on || id:ebi−a−GCST90001555

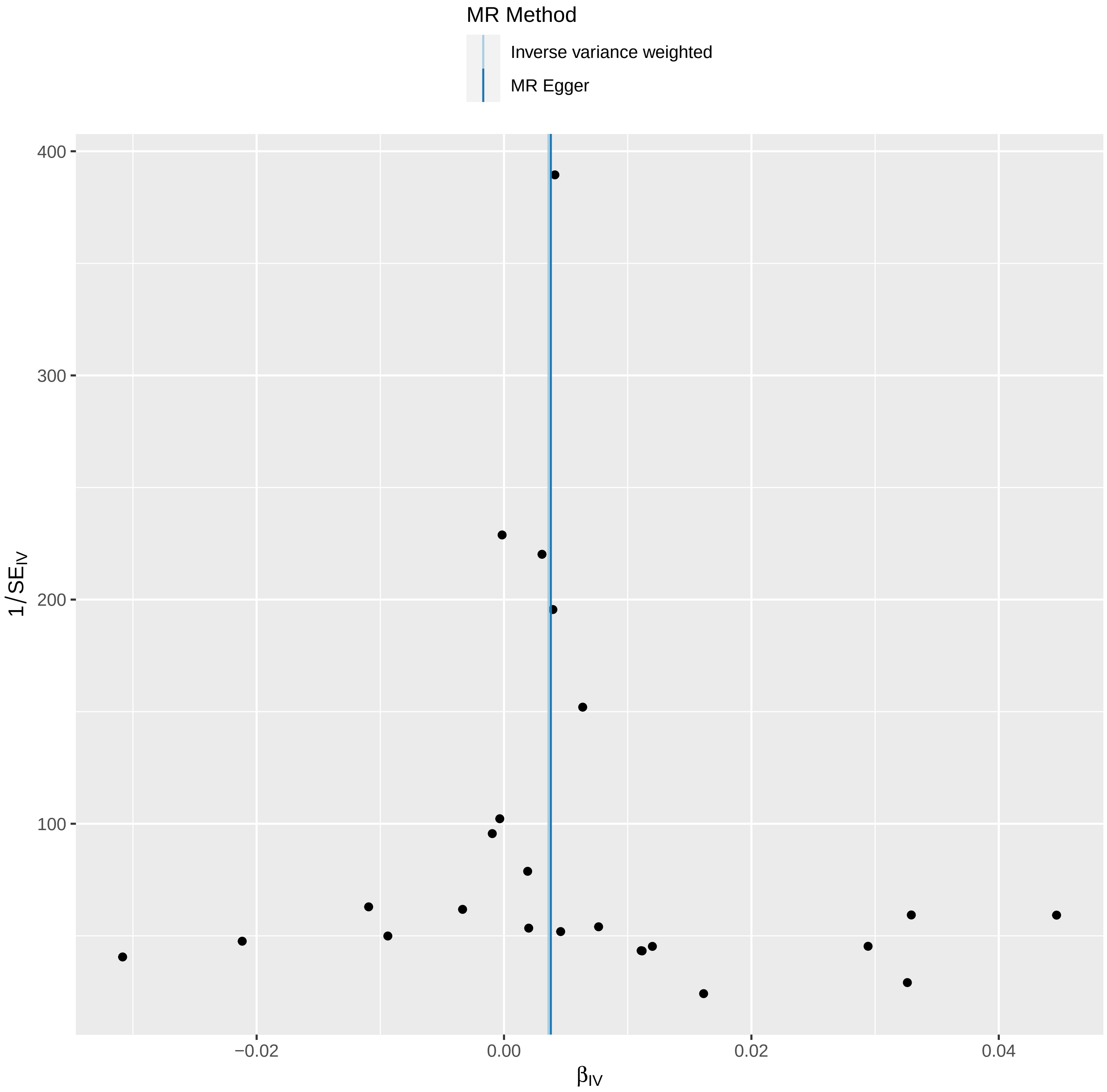

MR Method
Inverse variance weighted
MR Egger
400
300
200
100
1/SE_IV
−0.02
0.00
0.02
0.04
β_IV

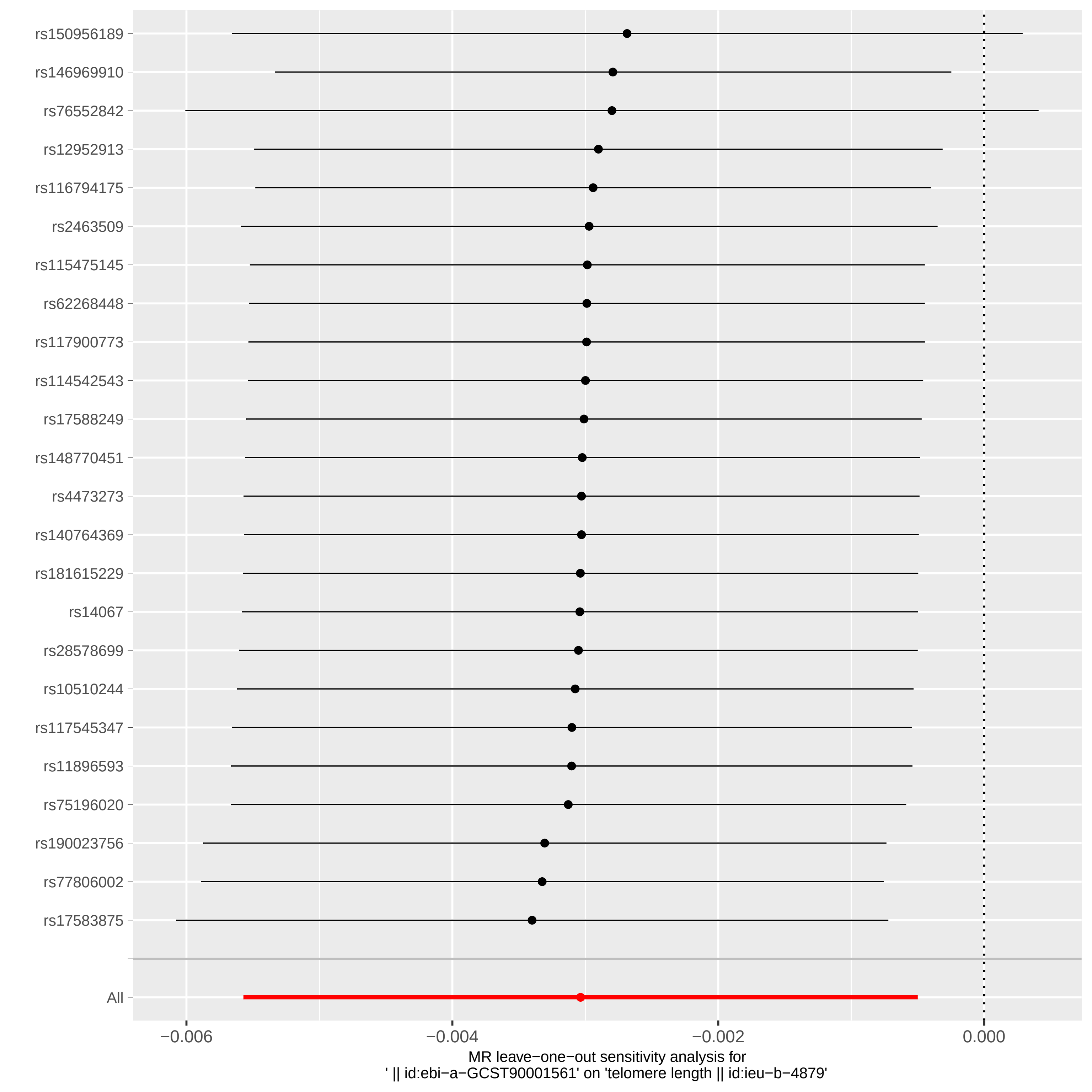
rs150956189
rs146969910
rs76552842
rs12952913
rs116794175
rs2463509
rs115475145
rs62268448
rs117900773
rs114542543
rs17588249
rs148770451
rs4473273
rs140764369
rs181615229
rs14067
rs28578699
rs10510244
rs117545347
rs11896593
rs75196020
rs190023756
rs77806002
rs17583875
All
−0.006
−0.004
−0.002
0.000
MR leave−one−out sensitivity analysis for
' || id:ebi−a−GCST90001561' on 'telomere length || id:ieu−b−4879'

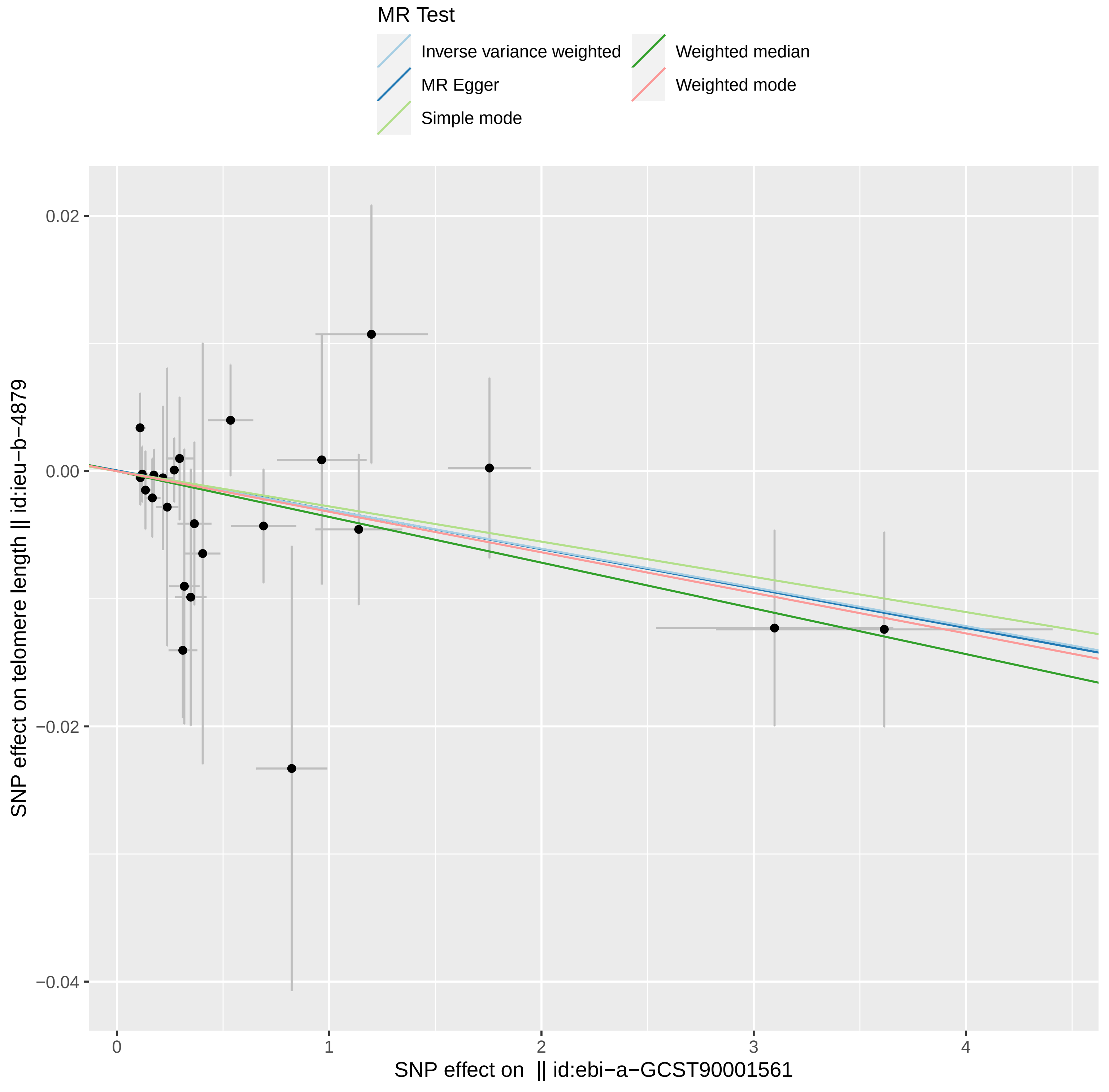
MR Test
Inverse variance weighted
MR Egger
Simple mode
Weighted median
Weighted mode
0.02
0.00
−0.02
−0.04
0
1
2
3
4
SNP effect on telomere length || id:ieu−b−4879
SNP effect on || id:ebi−a−GCST90001561

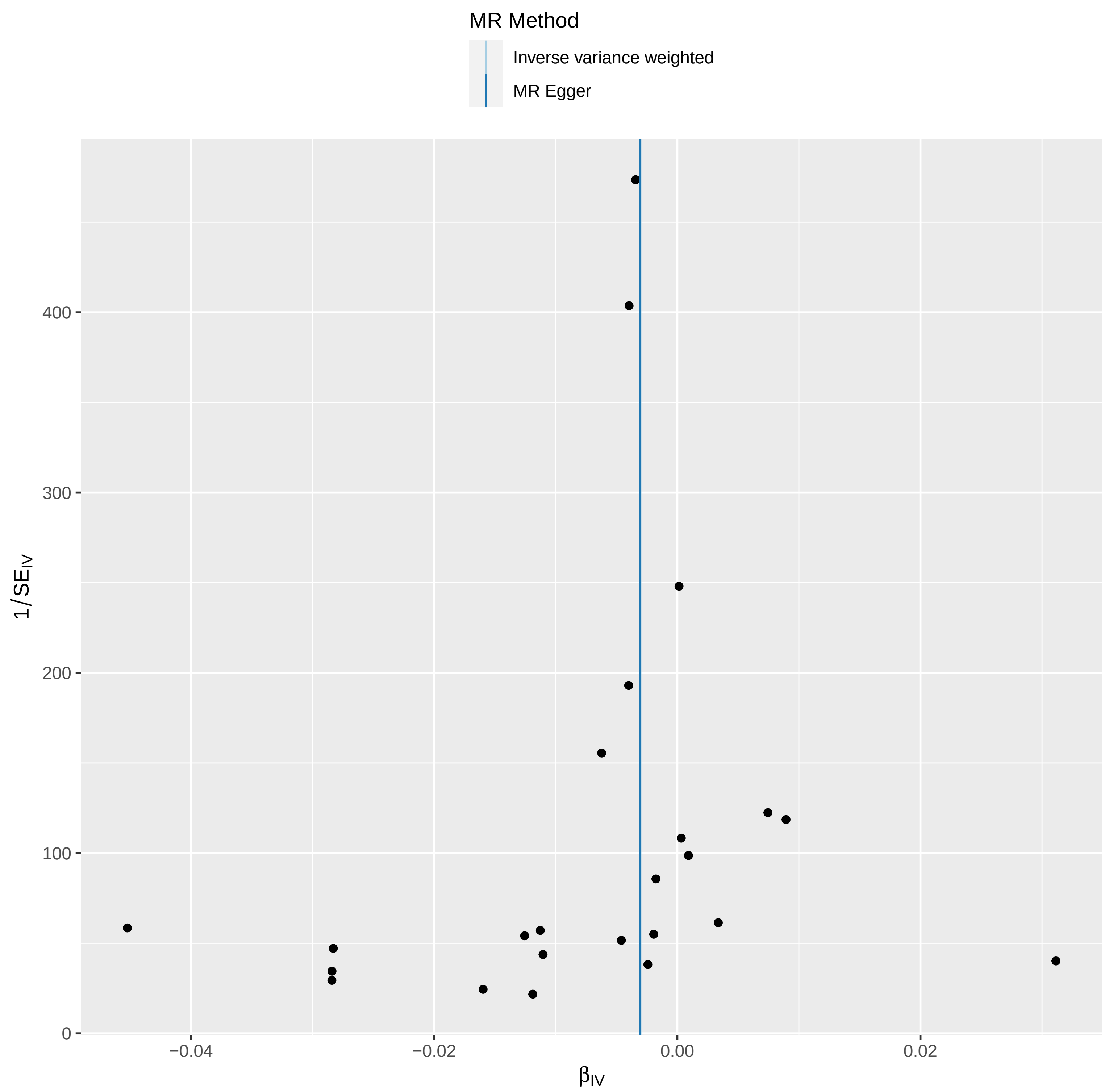

MR Method
Inverse variance weighted
MR Egger
400
300
200
100
0
$1/SE_{IV}$
−0.04
−0.02
0.00
0.02
β_{IV}

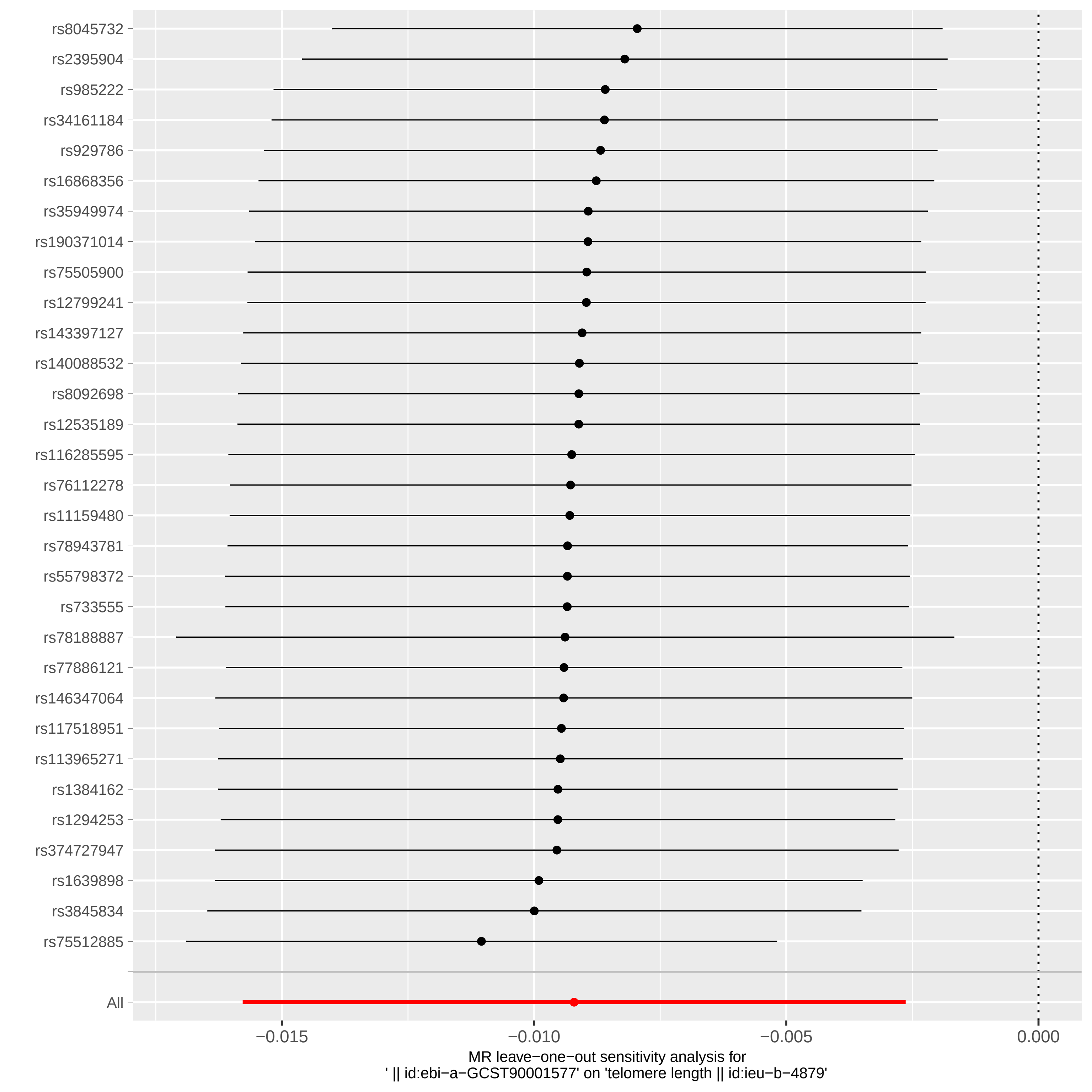
rs8045732
rs2395904
rs985222
rs34161184
rs929786
rs16868356
rs35949974
rs190371014
rs75505900
rs12799241
rs143397127
rs140088532
rs8092698
rs12535189
rs116285595
rs76112278
rs11159480
rs78943781
rs55798372
rs733555
rs78188887
rs77886121
rs146347064
rs117518951
rs113965271
rs1384162
rs1294253
rs374727947
rs1639898
rs3845834
rs75512885
All
−0.015
−0.010
−0.005
0.000
MR leave−one−out sensitivity analysis for
' || id:ebi−a−GCST90001577' on 'telomere length || id:ieu−b−4879'

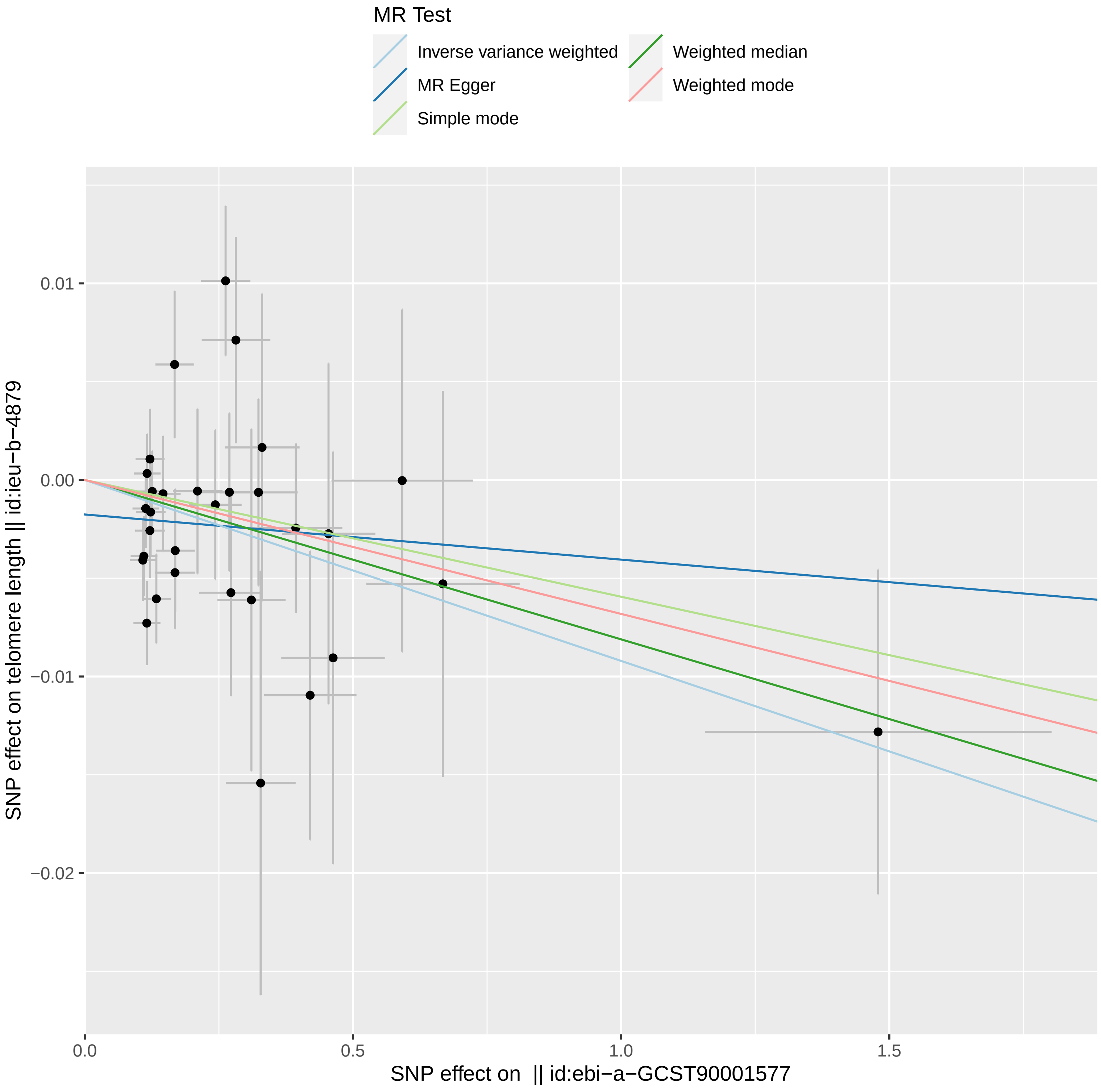
MR Test
Inverse variance weighted
MR Egger
Simple mode
Weighted median
Weighted mode
SNP effect on telomere length || id:ieu−b−4879
0.01
0.00
−0.01
−0.02
0.0
0.5
1.0
1.5
SNP effect on || id:ebi−a−GCST90001577

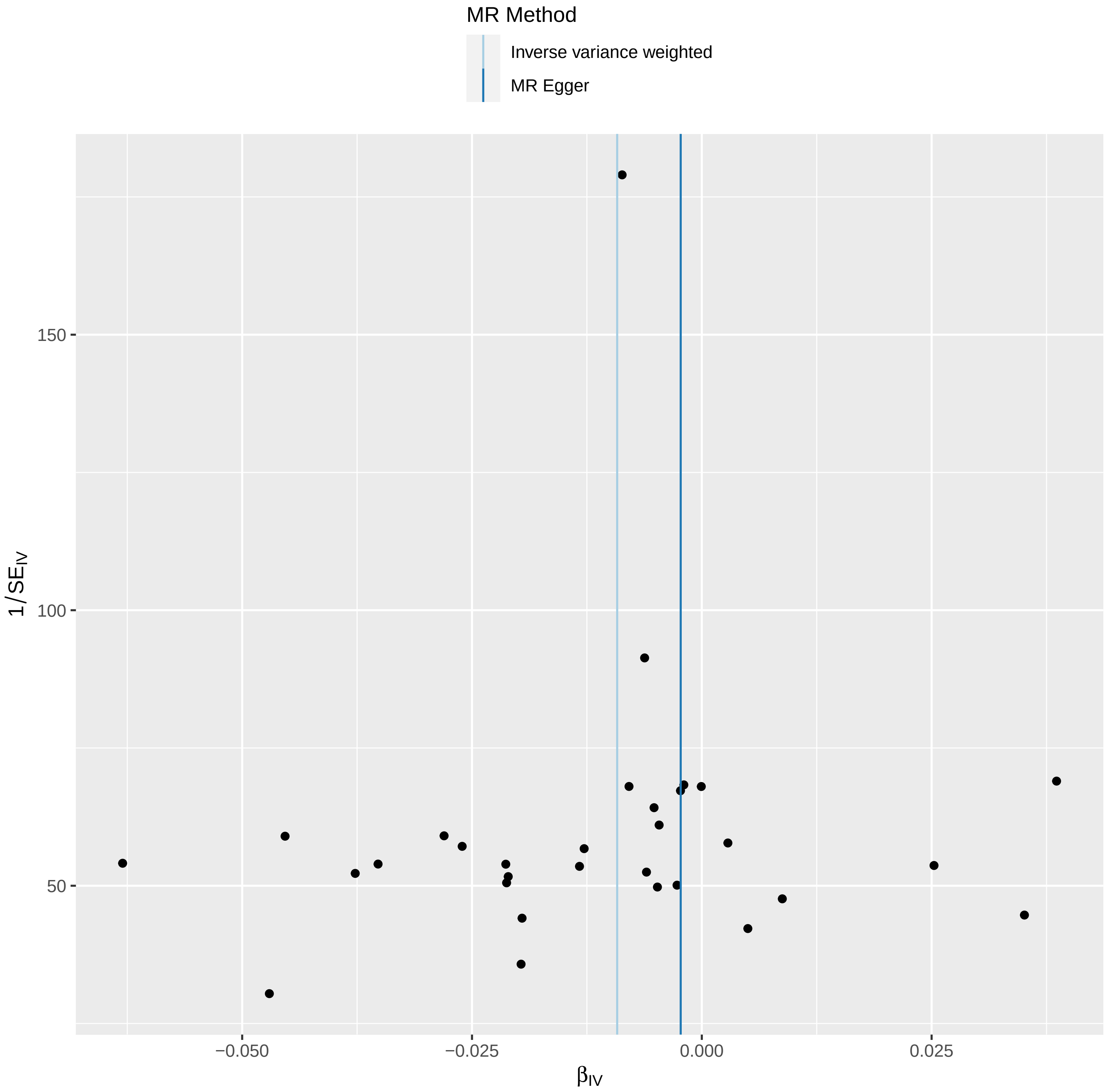
MR Method
Inverse variance weighted
MR Egger
150
100
50
1/SE_IV
−0.050
−0.025
0.000
0.025
β_IV

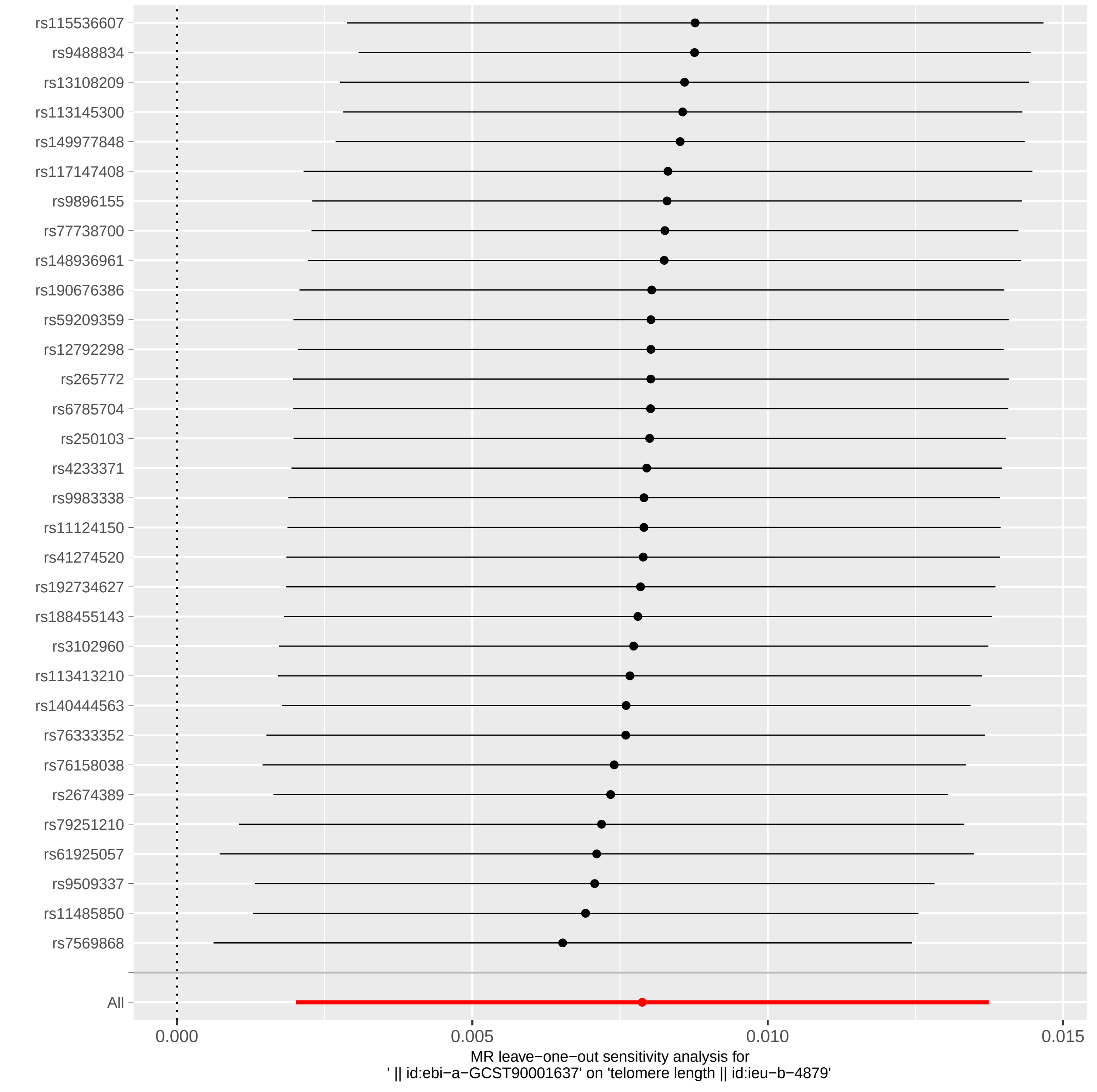
rs115536607
rs9488834
rs13108209
rs113145300
rs149977848
rs117147408
rs9896155
rs77738700
rs148936961
rs190676386
rs59209359
rs12792298
rs265772
rs6785704
rs250103
rs4233371
rs9983338
rs11124150
rs41274520
rs192734627
rs188455143
rs3102960
rs113413210
rs140444563
rs76333352
rs76158038
rs2674389
rs79251210
rs61925057
rs9509337
rs11485850
rs7569868
All
0.000
0.005
0.010
0.015
MR leave−one−out sensitivity analysis for
' || id:ebi−a−GCST90001637' on 'telomere length || id:ieu−b−4879'

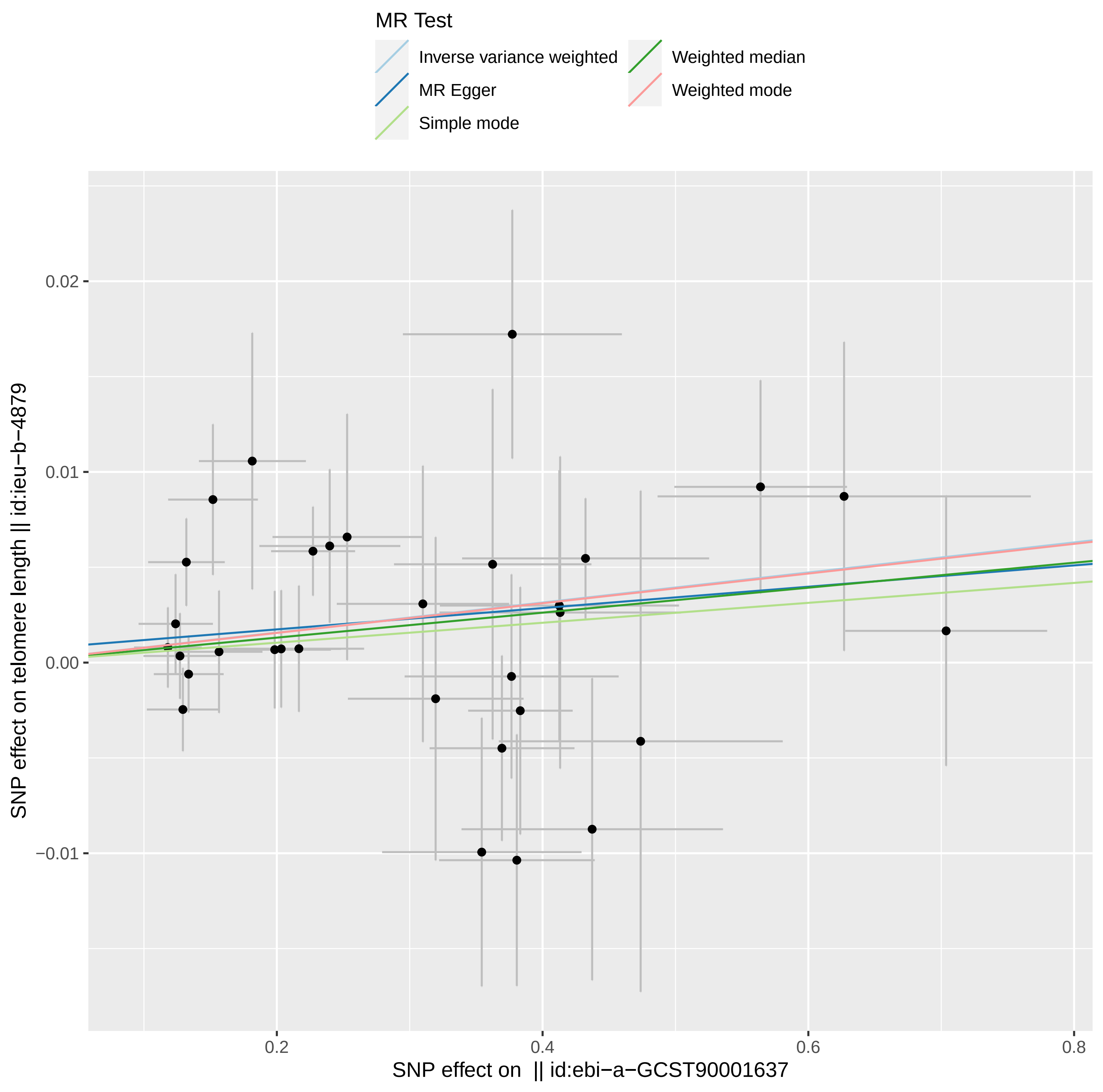
MR Test
Inverse variance weighted
MR Egger
Simple mode
Weighted median
Weighted mode
0.02
0.01
0.00
−0.01
0.2
0.4
0.6
0.8
SNP effect on telomere length || id:ieu−b−4879
SNP effect on || id:ebi−a−GCST90001637

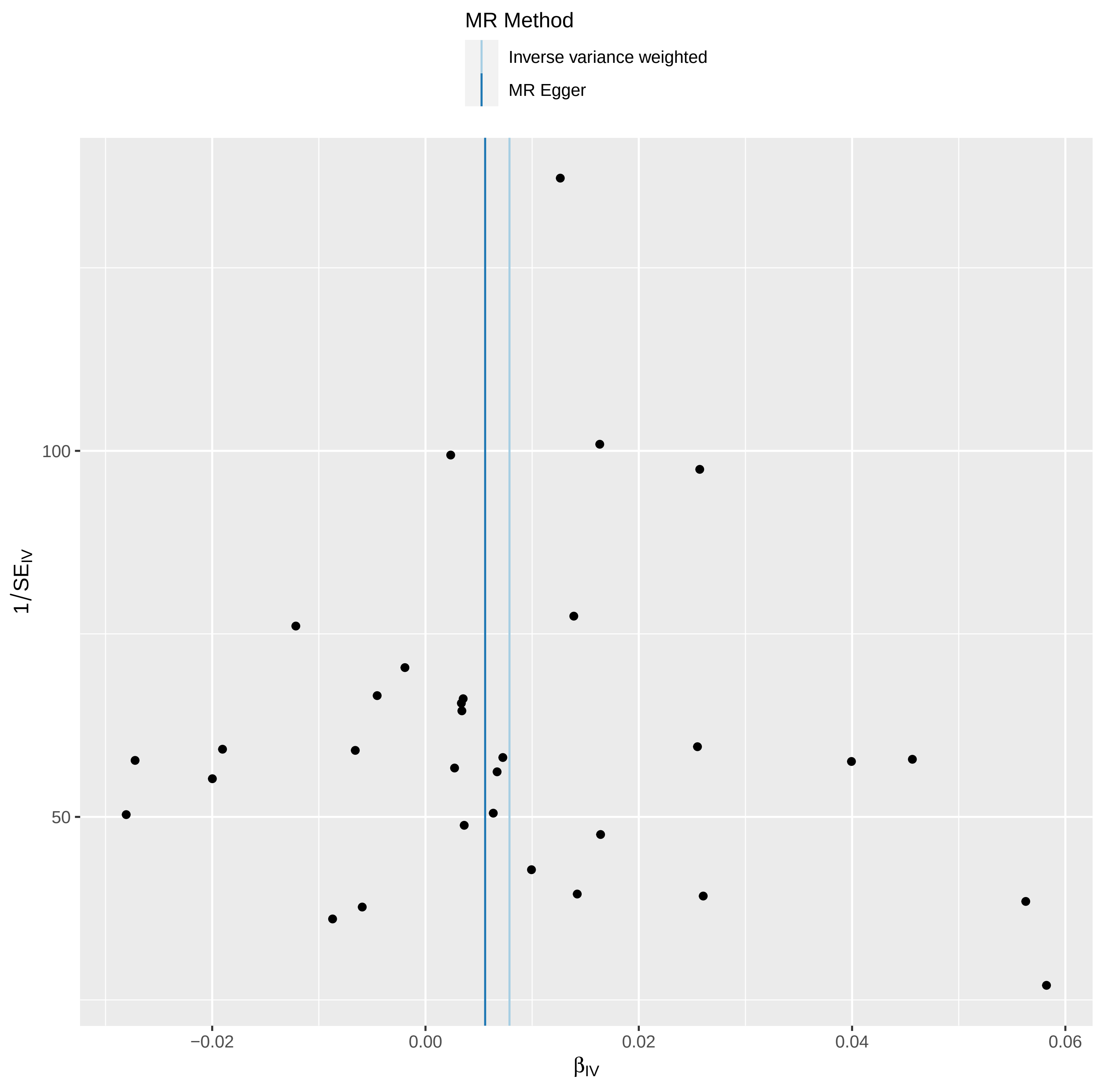
MR Method
Inverse variance weighted
MR Egger
1/SE_IV
100
50
β_IV
−0.02
0.00
0.02
0.04
0.06

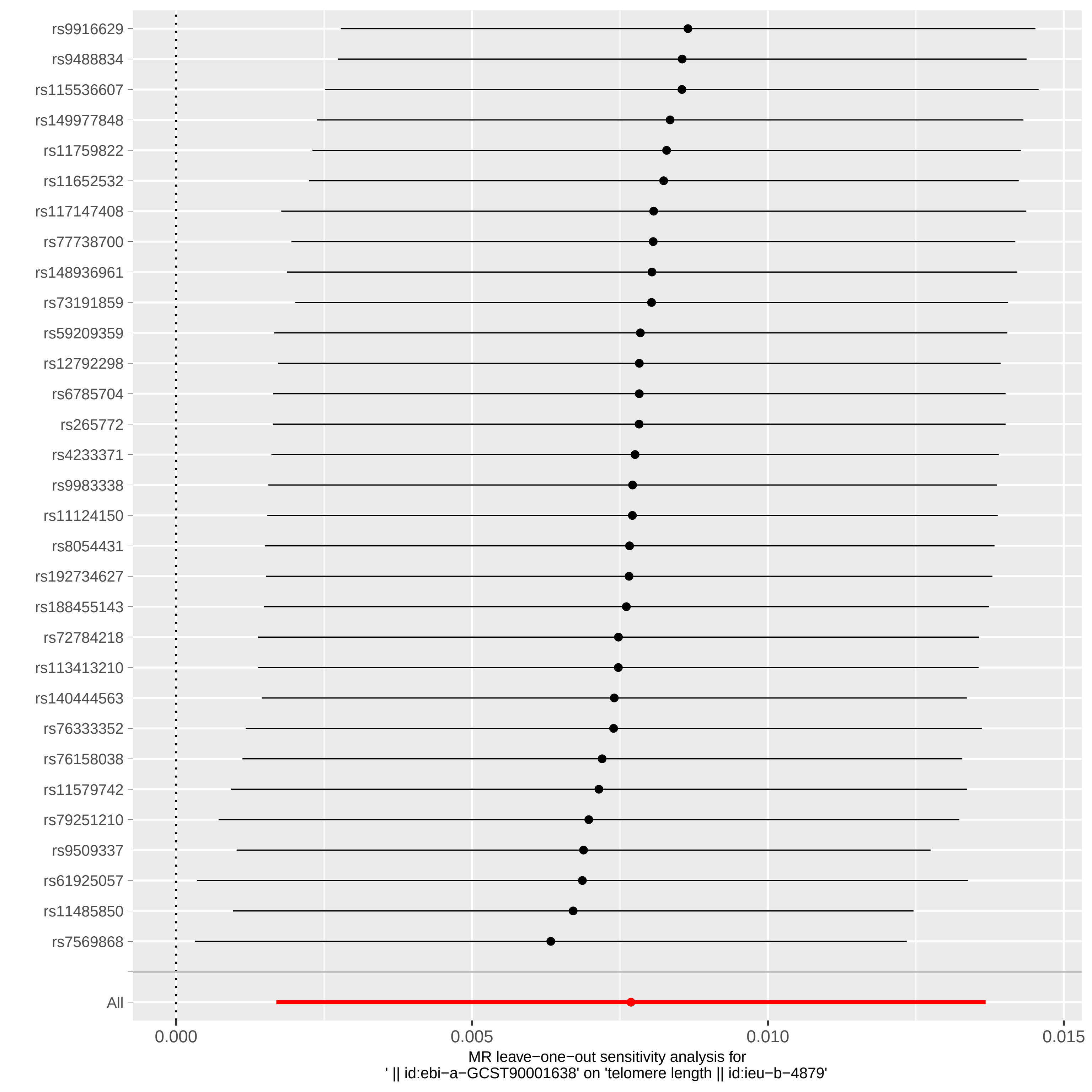
rs9916629
rs9488834
rs115536607
rs149977848
rs11759822
rs11652532
rs117147408
rs77738700
rs148936961
rs73191859
rs59209359
rs12792298
rs6785704
rs265772
rs4233371
rs9983338
rs11124150
rs8054431
rs192734627
rs188455143
rs72784218
rs113413210
rs140444563
rs76333352
rs76158038
rs11579742
rs79251210
rs9509337
rs61925057
rs11485850
rs7569868
All
0.000
0.005
0.010
0.015
MR leave−one−out sensitivity analysis for
' || id:ebi−a−GCST90001638' on 'telomere length || id:ieu−b−4879'

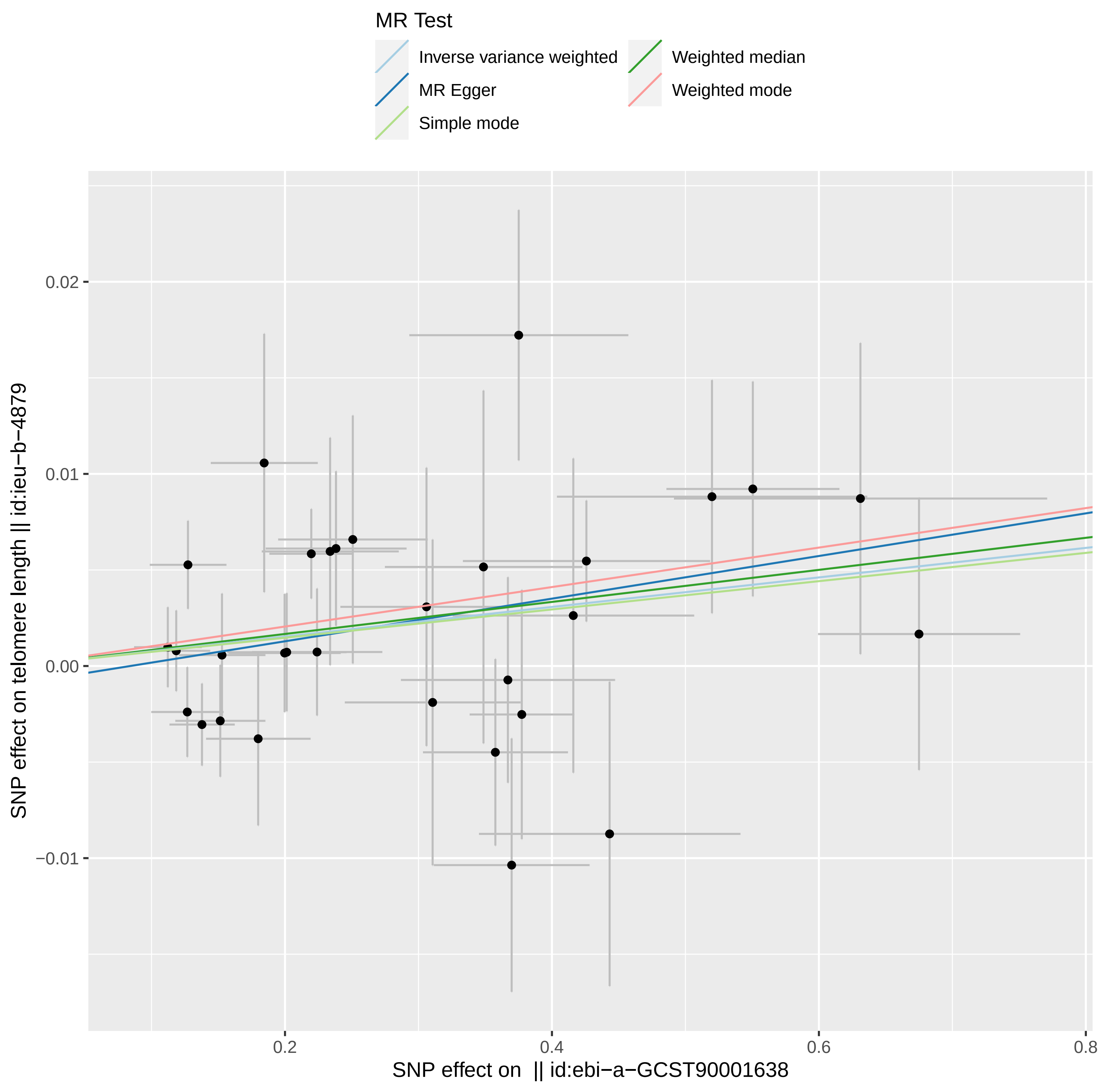

MR Test
Inverse variance weighted
MR Egger
Simple mode
Weighted median
Weighted mode
SNP effect on telomere length || id:ieu−b−4879
0.02
0.01
0.00
−0.01
0.2
0.4
0.6
0.8
SNP effect on || id:ebi−a−GCST90001638

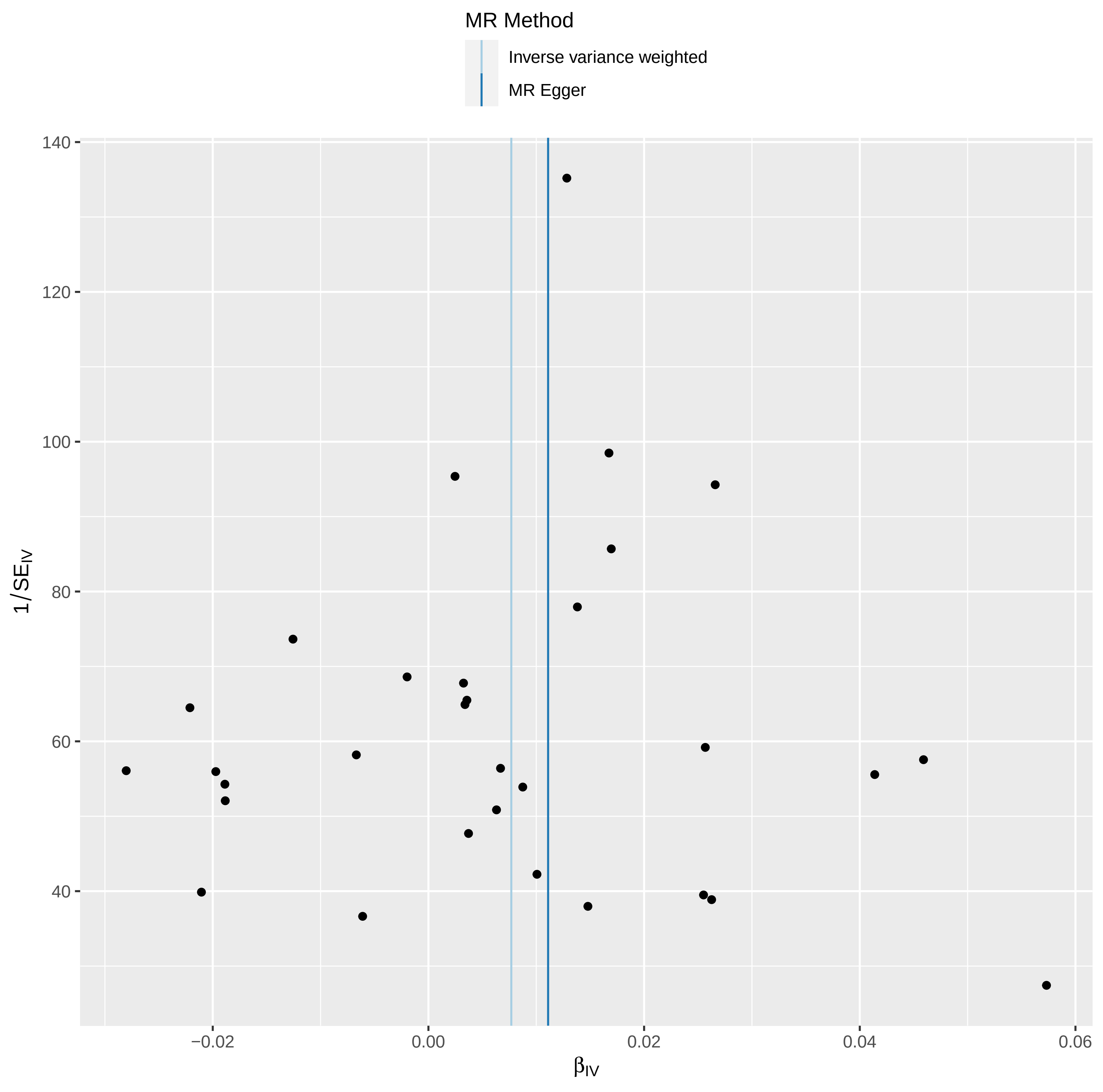
MR Method
Inverse variance weighted
MR Egger
140
120
100
80
60
40
1/SE$_{IV}$
−0.02
0.00
0.02
0.04
0.06
β$_{IV}$

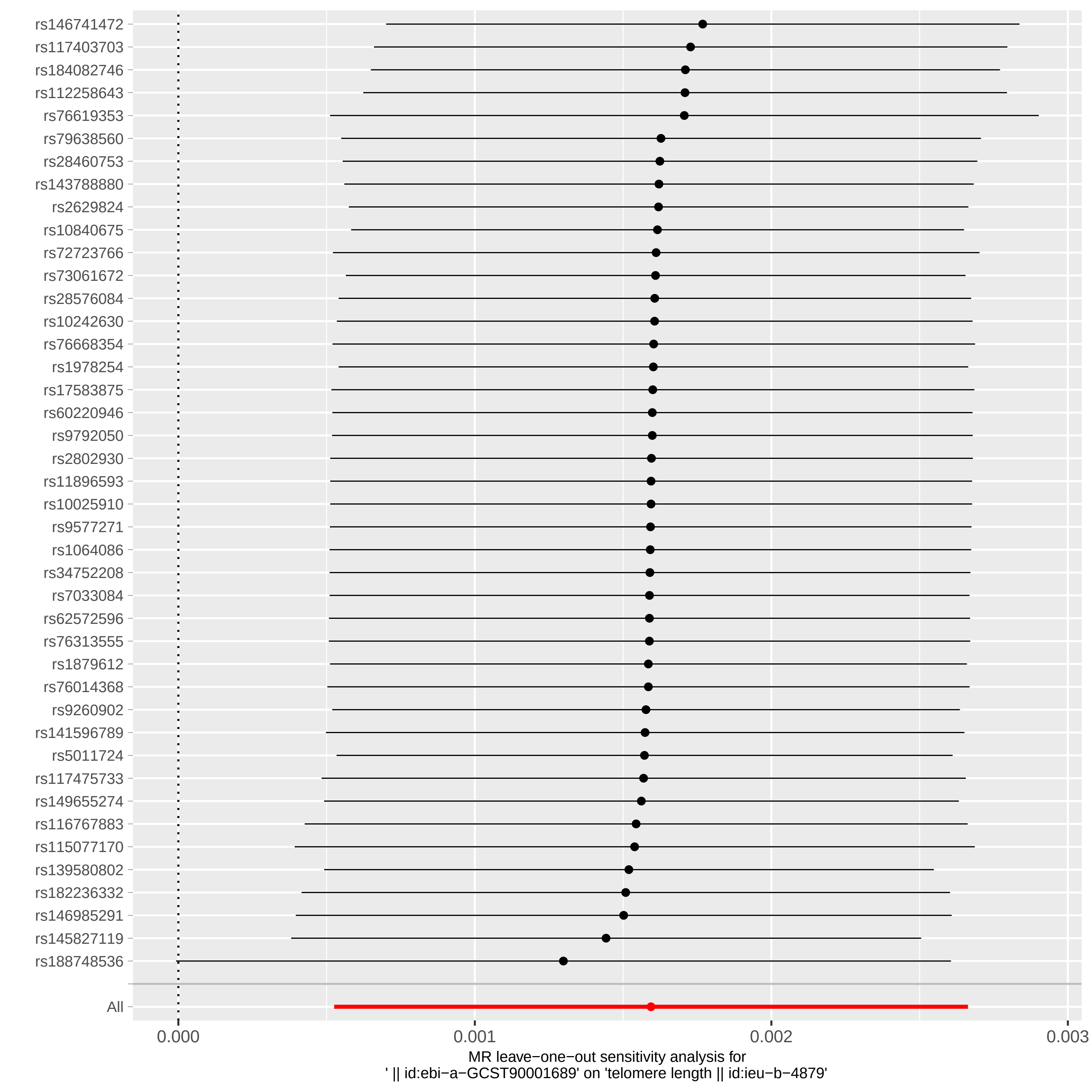
rs146741472
rs117403703
rs184082746
rs112258643
rs76619353
rs79638560
rs28460753
rs143788880
rs2629824
rs10840675
rs72723766
rs73061672
rs28576084
rs10242630
rs76668354
rs1978254
rs17583875
rs60220946
rs9792050
rs2802930
rs11896593
rs10025910
rs9577271
rs1064086
rs34752208
rs7033084
rs62572596
rs76313555
rs1879612
rs76014368
rs9260902
rs141596789
rs5011724
rs117475733
rs149655274
rs116767883
rs115077170
rs139580802
rs182236332
rs146985291
rs145827119
rs188748536
All
0.000
0.001
0.002
0.003
MR leave−one−out sensitivity analysis for
' || id:ebi−a−GCST90001689' on 'telomere length || id:ieu−b−4879'

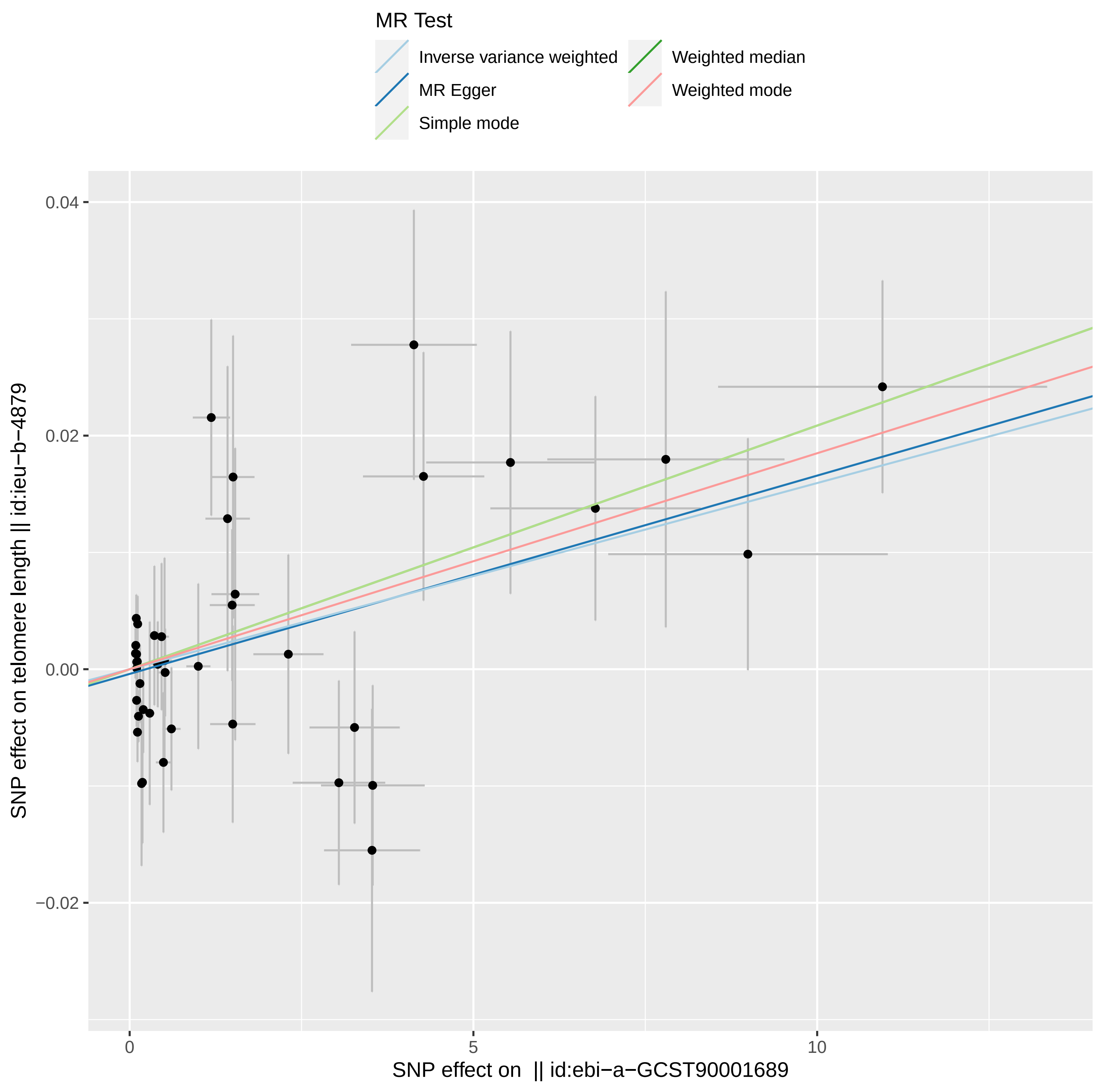

MR Test
Inverse variance weighted
MR Egger
Simple mode
Weighted median
Weighted mode
0.04
0.02
0.00
−0.02
0
5
10
SNP effect on telomere length || id:ieu−b−4879
SNP effect on || id:ebi−a−GCST90001689

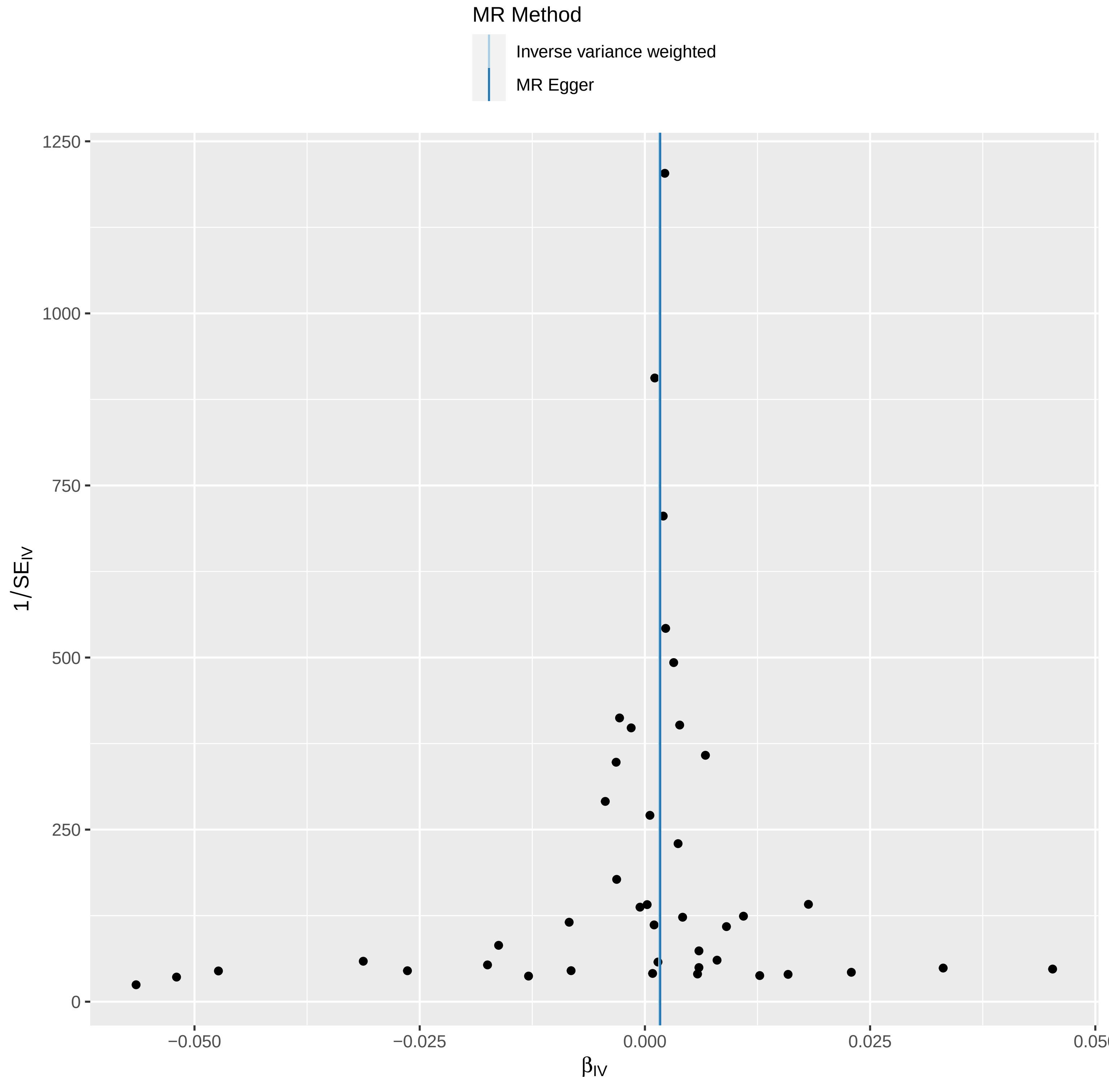

MR Method
Inverse variance weighted
MR Egger
1250
1000
750
500
250
0
1/SE_IV
−0.050
−0.025
0.000
0.025
0.050
β_IV

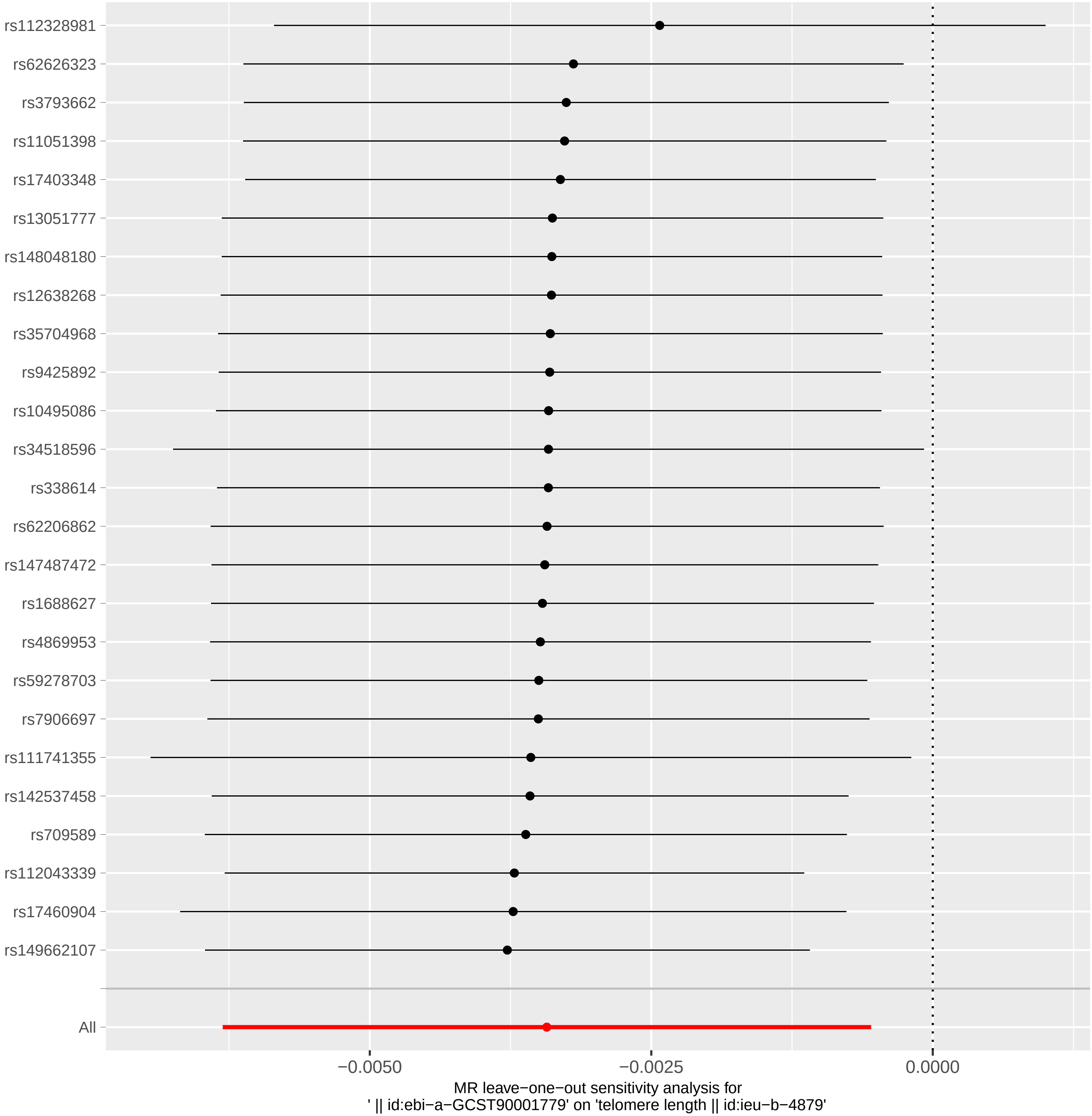
rs112328981
rs62626323
rs3793662
rs11051398
rs17403348
rs13051777
rs148048180
rs12638268
rs35704968
rs9425892
rs10495086
rs34518596
rs338614
rs62206862
rs147487472
rs1688627
rs4869953
rs59278703
rs7906697
rs111741355
rs142537458
rs709589
rs112043339
rs17460904
rs149662107
All
−0.0050
−0.0025
0.0000
MR leave−one−out sensitivity analysis for
' || id:ebi−a−GCST90001779' on 'telomere length || id:ieu−b−4879'

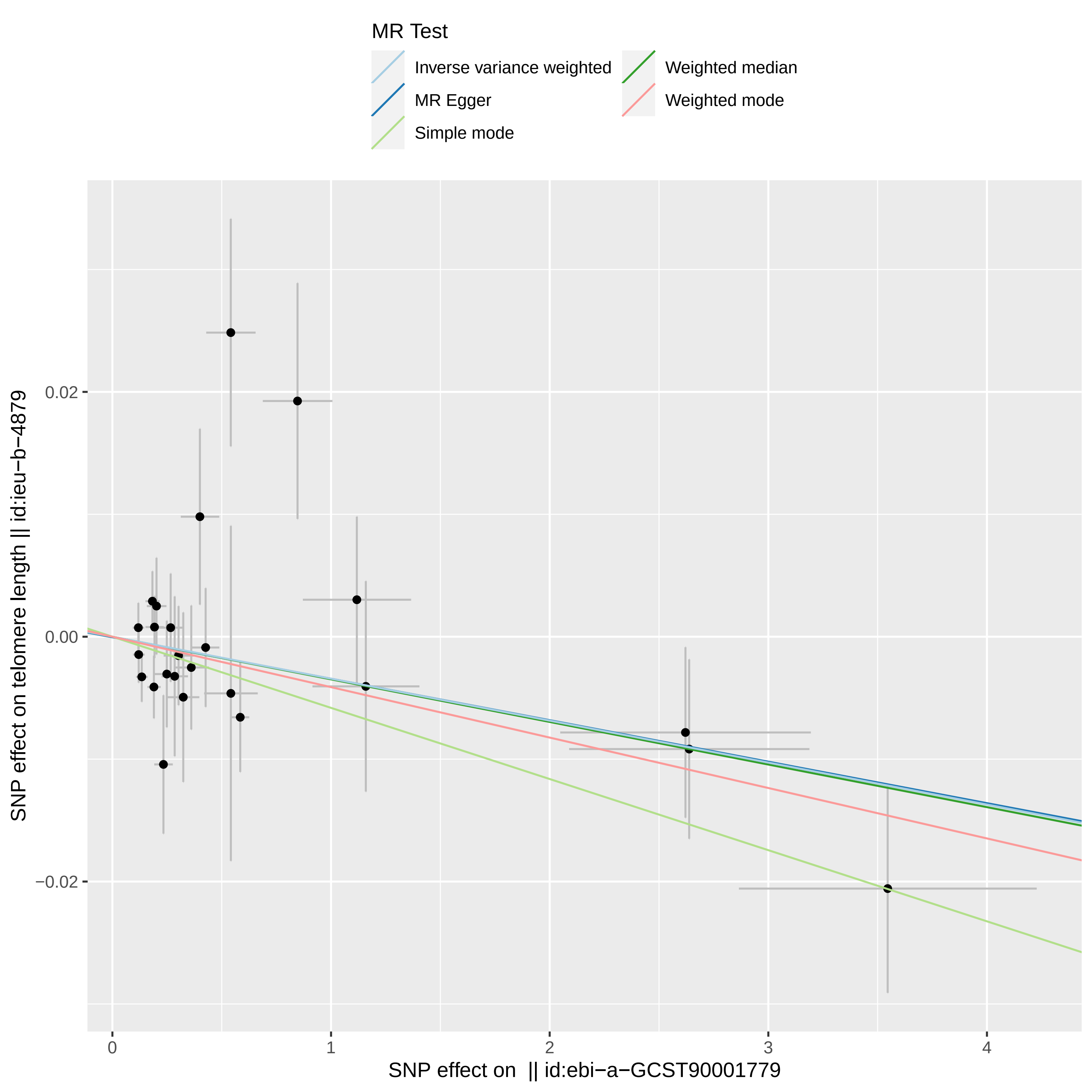
MR Test
Inverse variance weighted
MR Egger
Simple mode
Weighted median
Weighted mode
SNP effect on telomere length || id:ieu−b−4879
0.02
0.00
−0.02
0
1
2
3
4
SNP effect on || id:ebi−a−GCST90001779

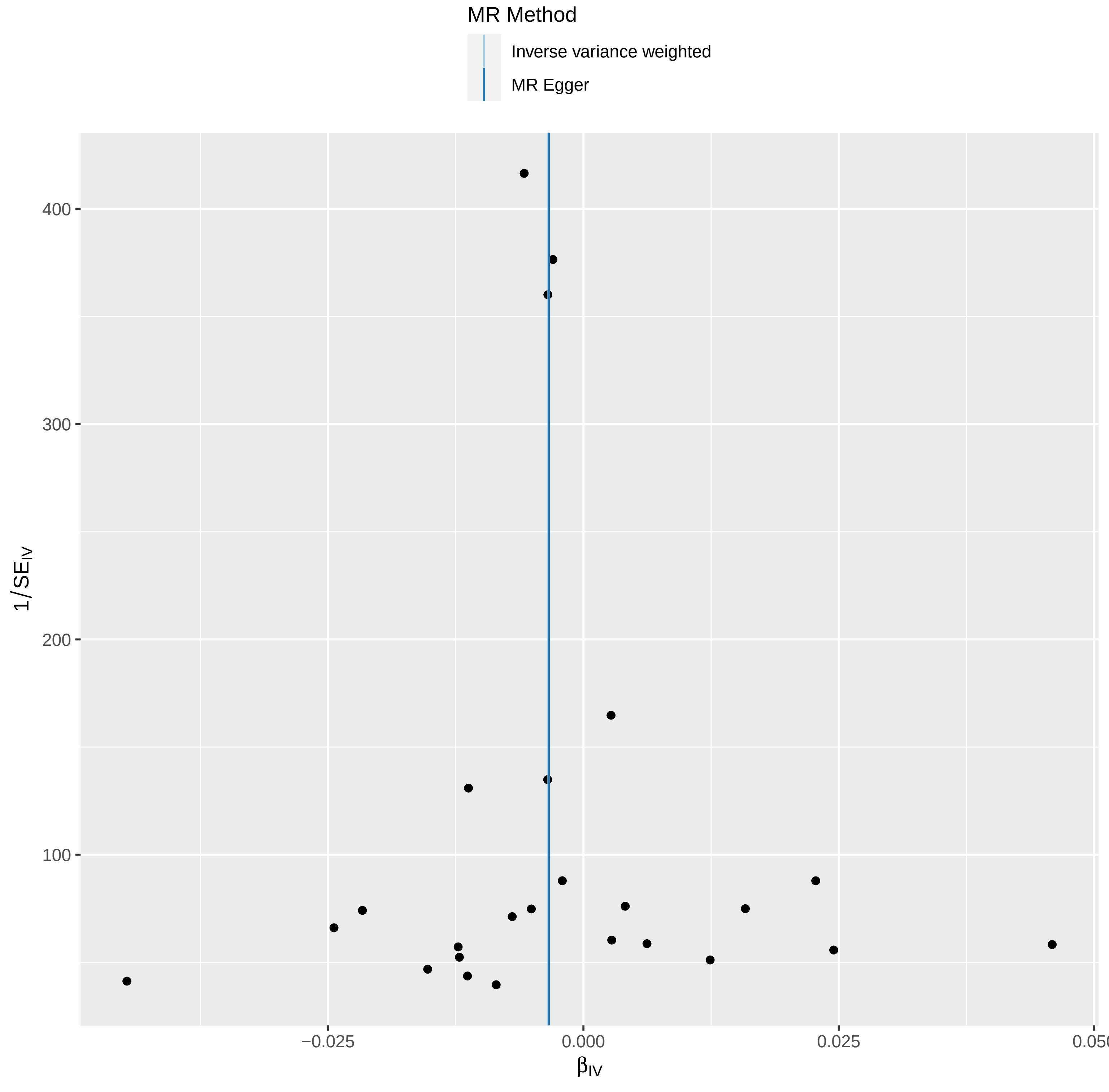
MR Method
Inverse variance weighted
MR Egger
400
300
200
100
$1/SE_{IV}$
−0.025
0.000
0.025
0.050
β_{IV}

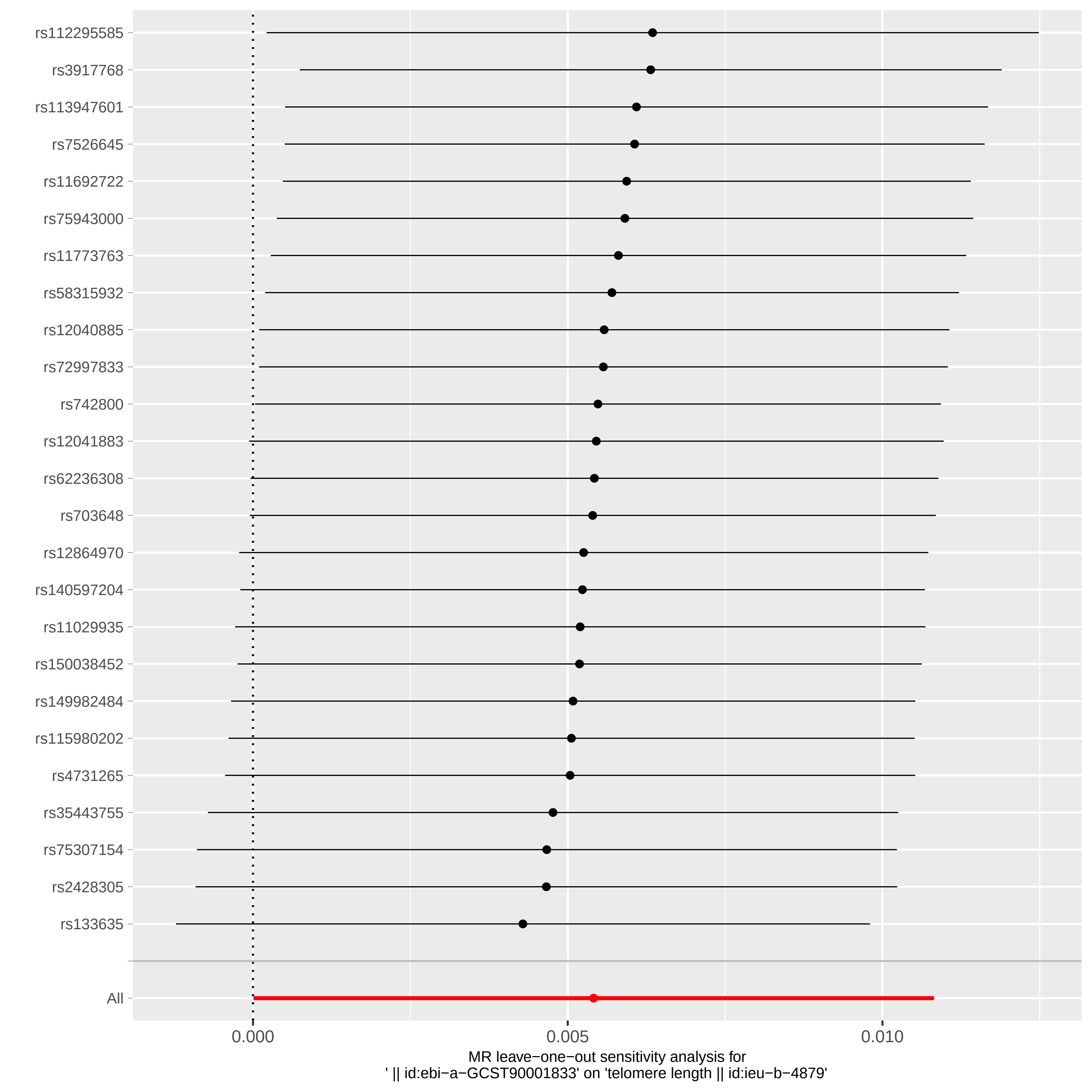
rs112295585
rs3917768
rs113947601
rs7526645
rs11692722
rs75943000
rs11773763
rs58315932
rs12040885
rs72997833
rs742800
rs12041883
rs62236308
rs703648
rs12864970
rs140597204
rs11029935
rs150038452
rs149982484
rs115980202
rs4731265
rs35443755
rs75307154
rs2428305
rs133635
All
0.000
0.005
0.010
MR leave−one−out sensitivity analysis for
' || id:ebi−a−GCST90001833' on 'telomere length || id:ieu−b−4879'

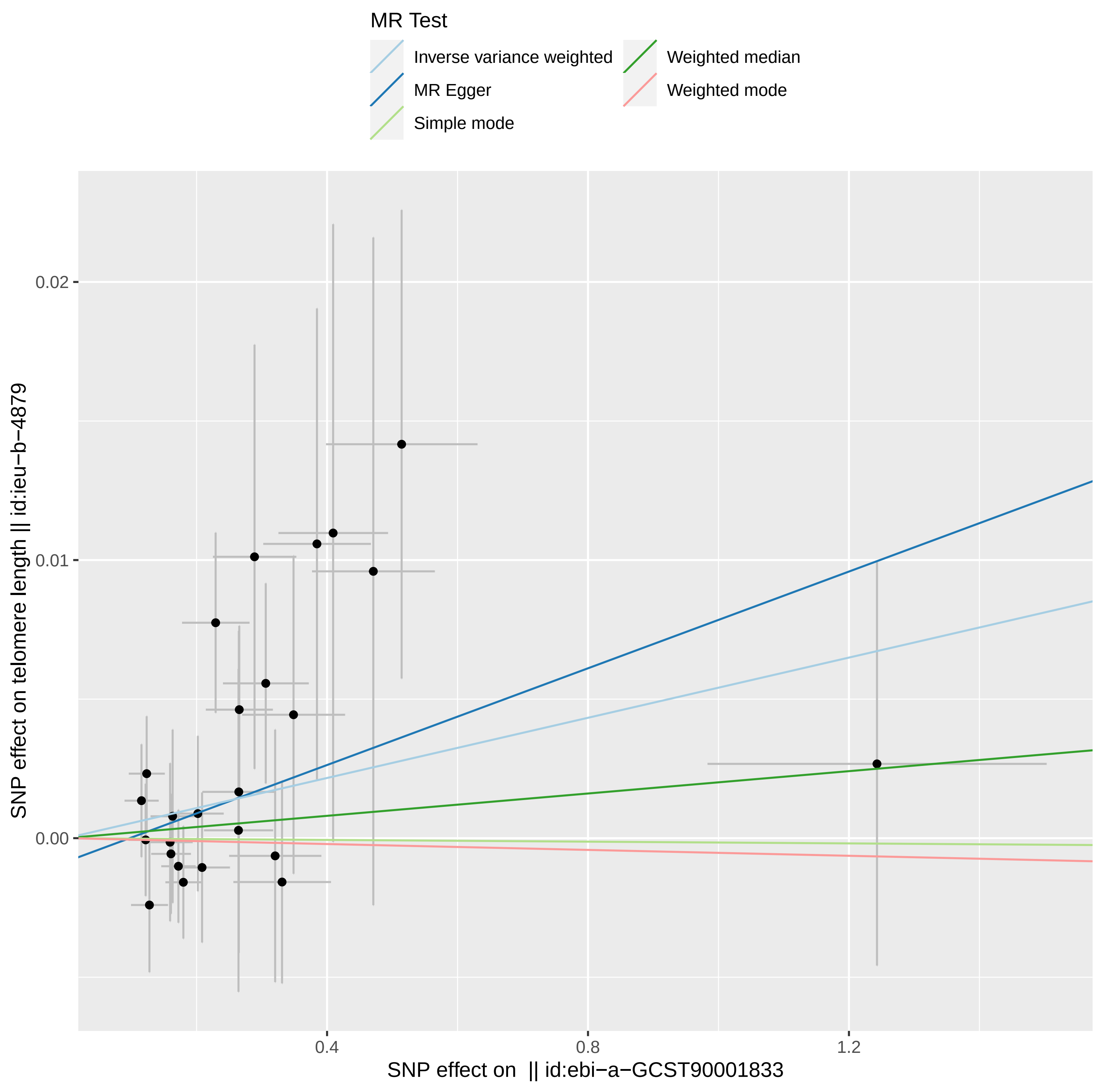
MR Test
Inverse variance weighted
MR Egger
Simple mode
Weighted median
Weighted mode
SNP effect on telomere length || id:ieu−b−4879
0.02
0.01
0.00
0.4
0.8
1.2
SNP effect on || id:ebi−a−GCST90001833

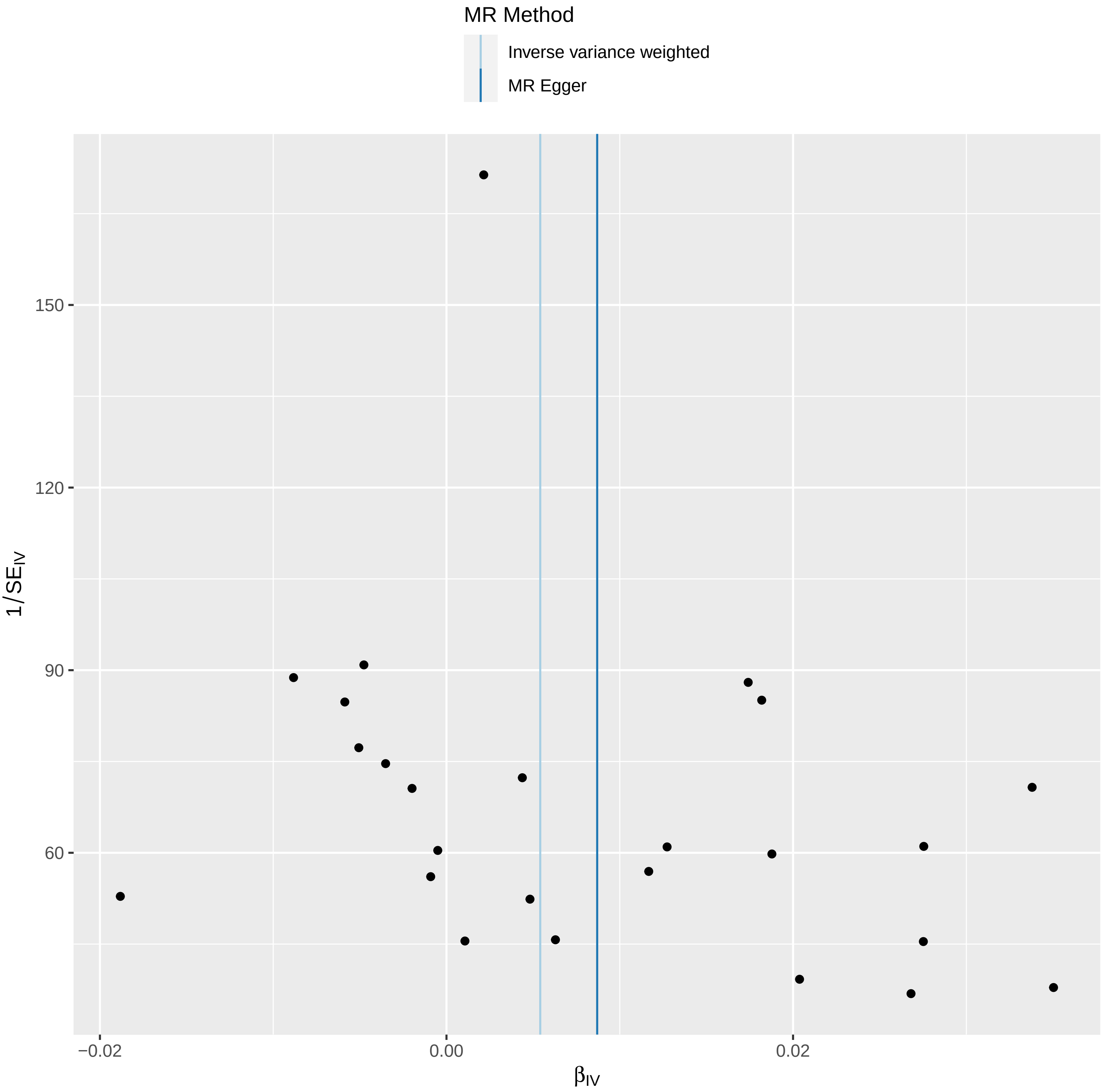

MR Method
Inverse variance weighted
MR Egger
150
120
90
60
1/SE$_{IV}$
−0.02
0.00
0.02
β$_{IV}$

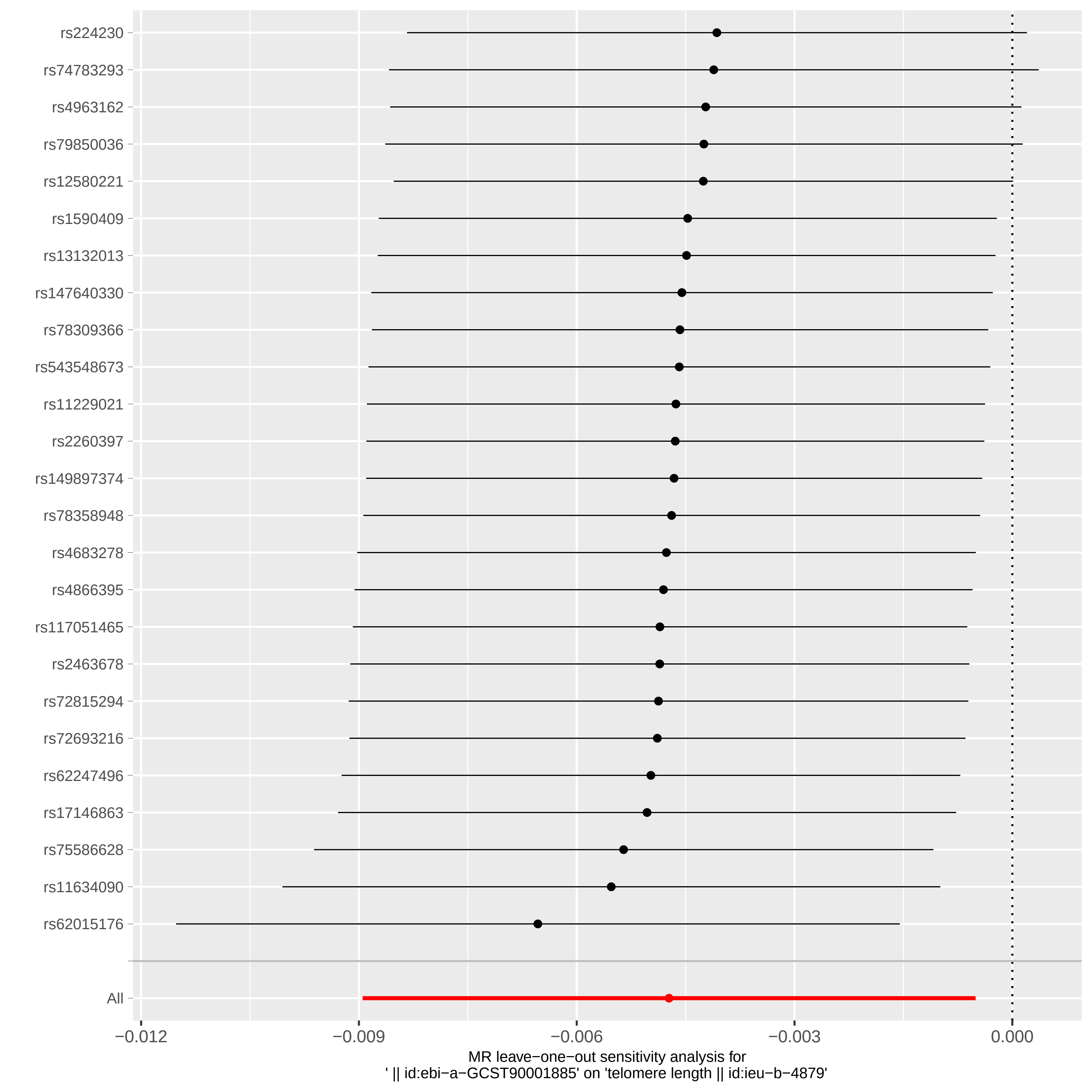
rs224230
rs74783293
rs4963162
rs79850036
rs12580221
rs1590409
rs13132013
rs147640330
rs78309366
rs543548673
rs11229021
rs2260397
rs149897374
rs78358948
rs4683278
rs4866395
rs117051465
rs2463678
rs72815294
rs72693216
rs62247496
rs17146863
rs75586628
rs11634090
rs62015176
All
−0.012
−0.009
−0.006
−0.003
0.000
MR leave−one−out sensitivity analysis for
' || id:ebi−a−GCST90001885' on 'telomere length || id:ieu−b−4879'

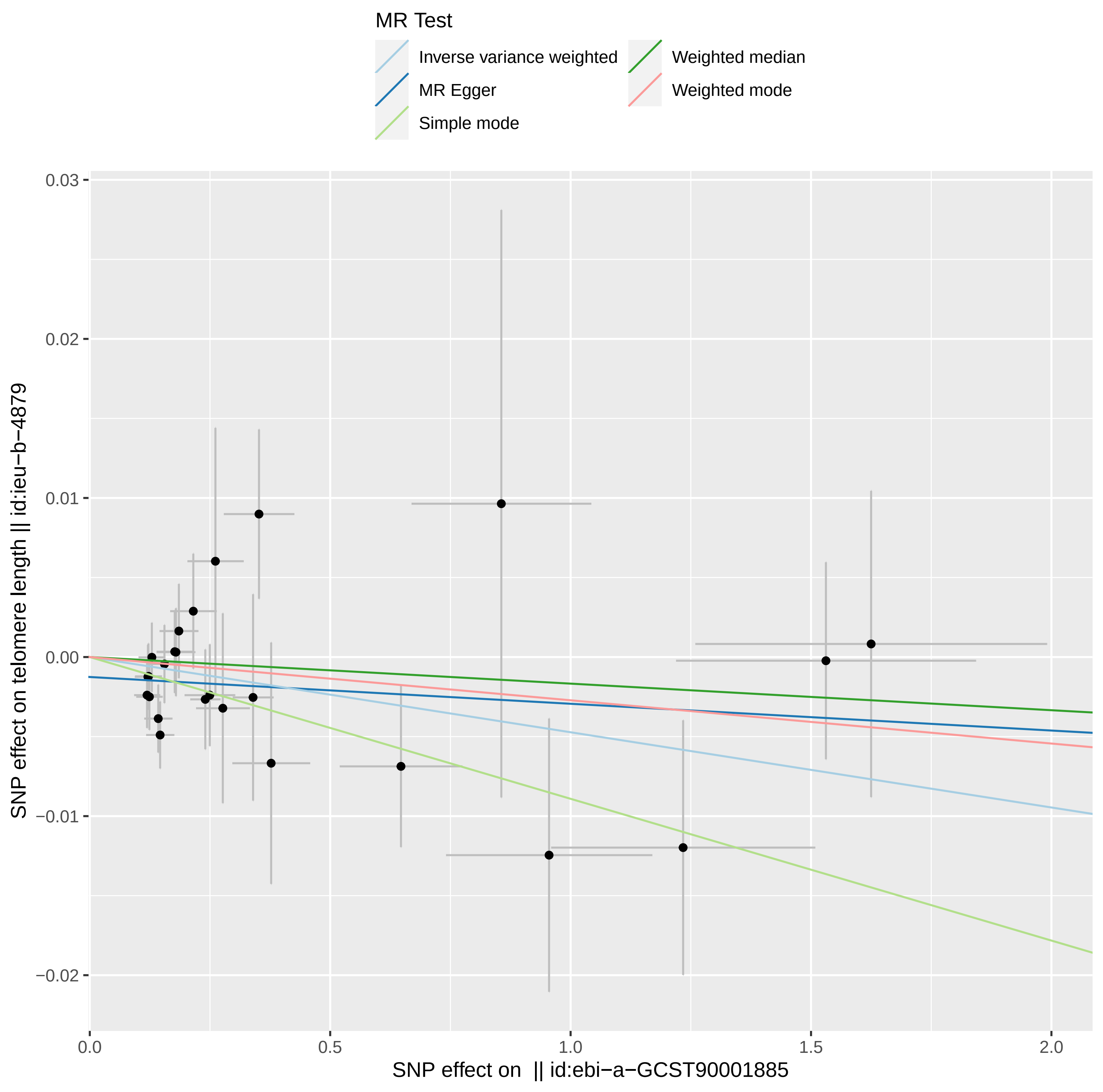
MR Test
Inverse variance weighted
MR Egger
Simple mode
Weighted median
Weighted mode
0.03
0.02
0.01
0.00
−0.01
−0.02
SNP effect on telomere length || id:ieu−b−4879
0.0
0.5
1.0
1.5
2.0
SNP effect on || id:ebi−a−GCST90001885

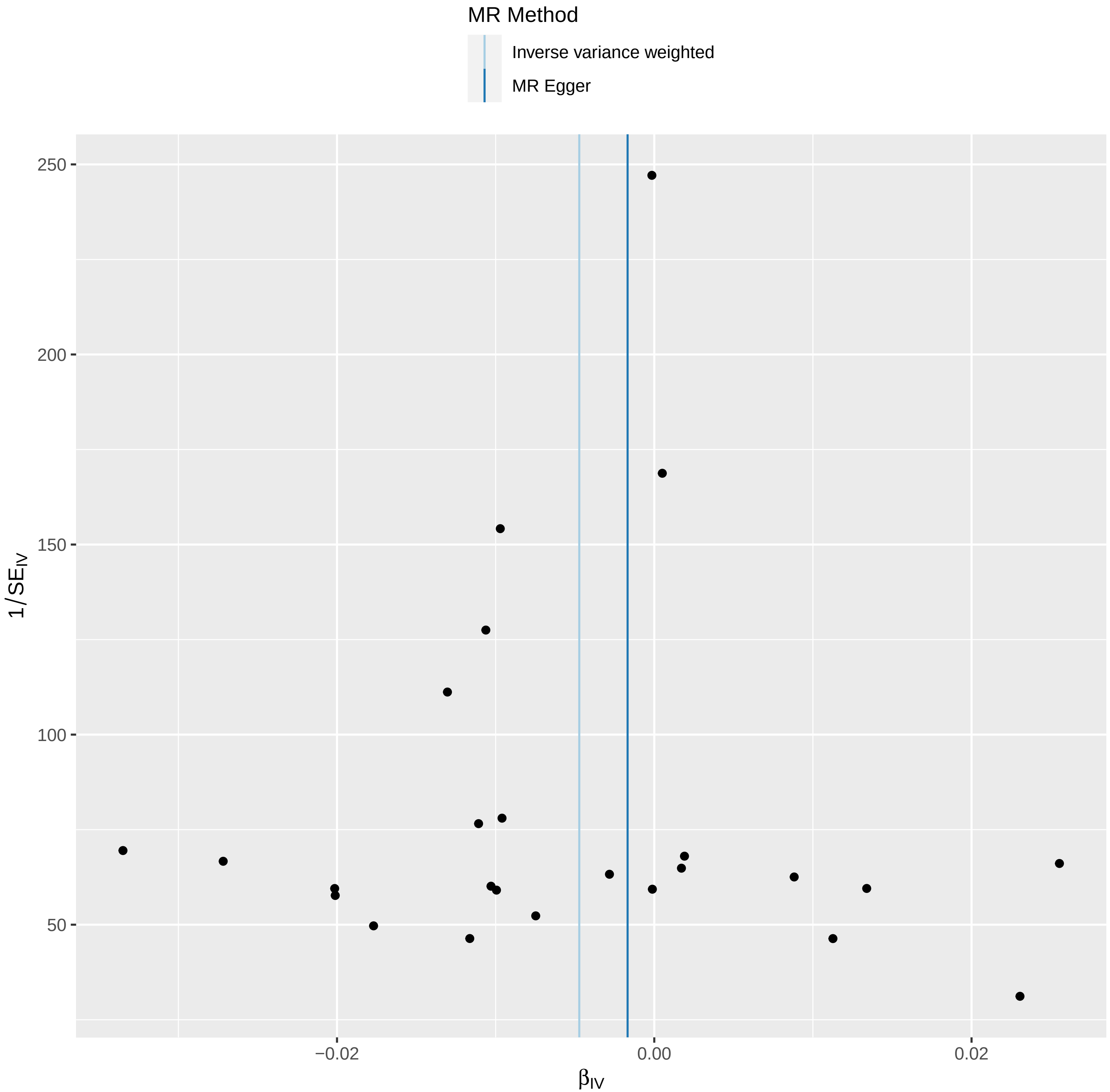
MR Method
Inverse variance weighted
MR Egger
250
200
150
100
50
1/SE$_{IV}$
−0.02
0.00
0.02
β$_{IV}$

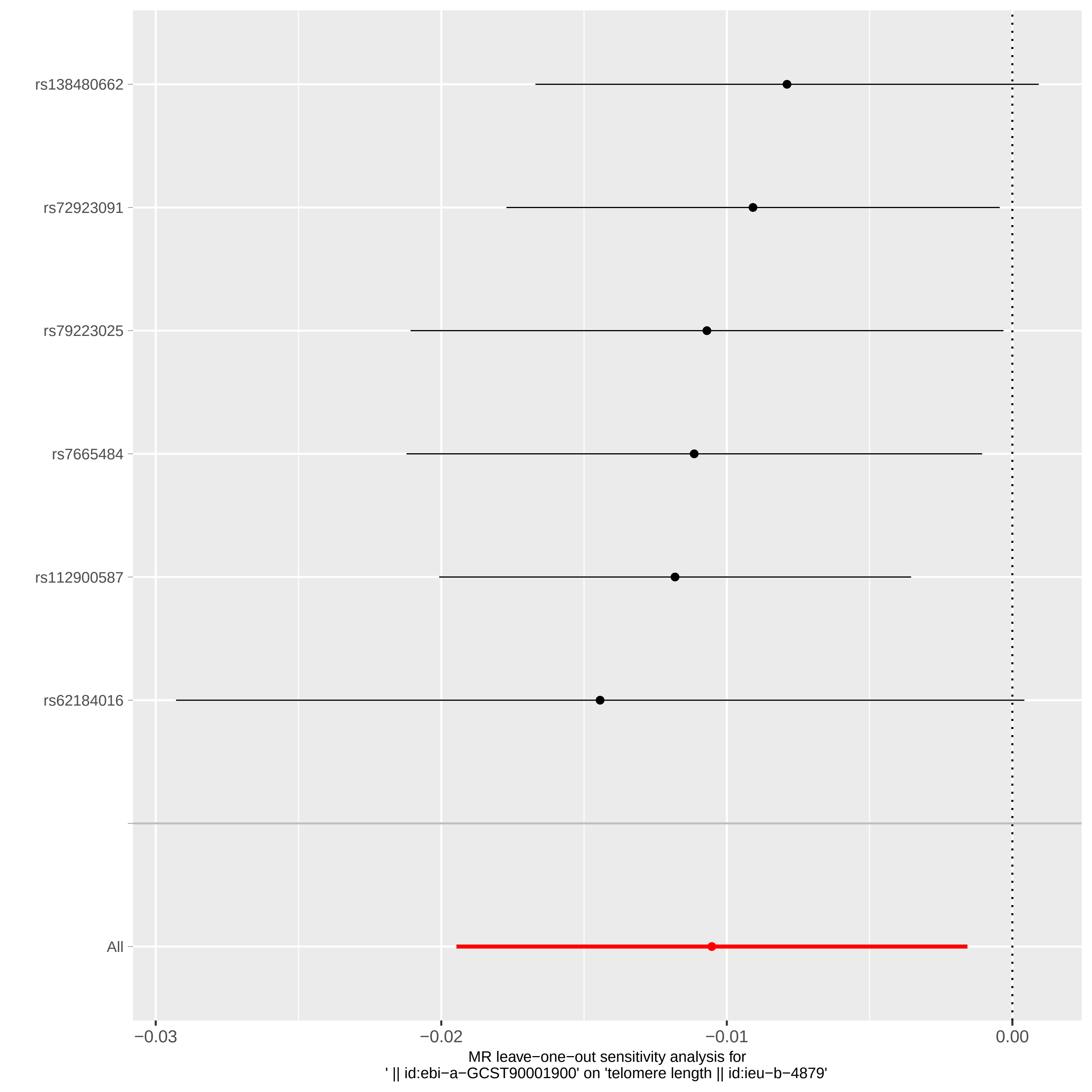

rs138480662
rs72923091
rs79223025
rs7665484
rs112900587
rs62184016
All
−0.03
−0.02
−0.01
0.00
MR leave−one−out sensitivity analysis for
' || id:ebi−a−GCST90001900' on 'telomere length || id:ieu−b−4879'

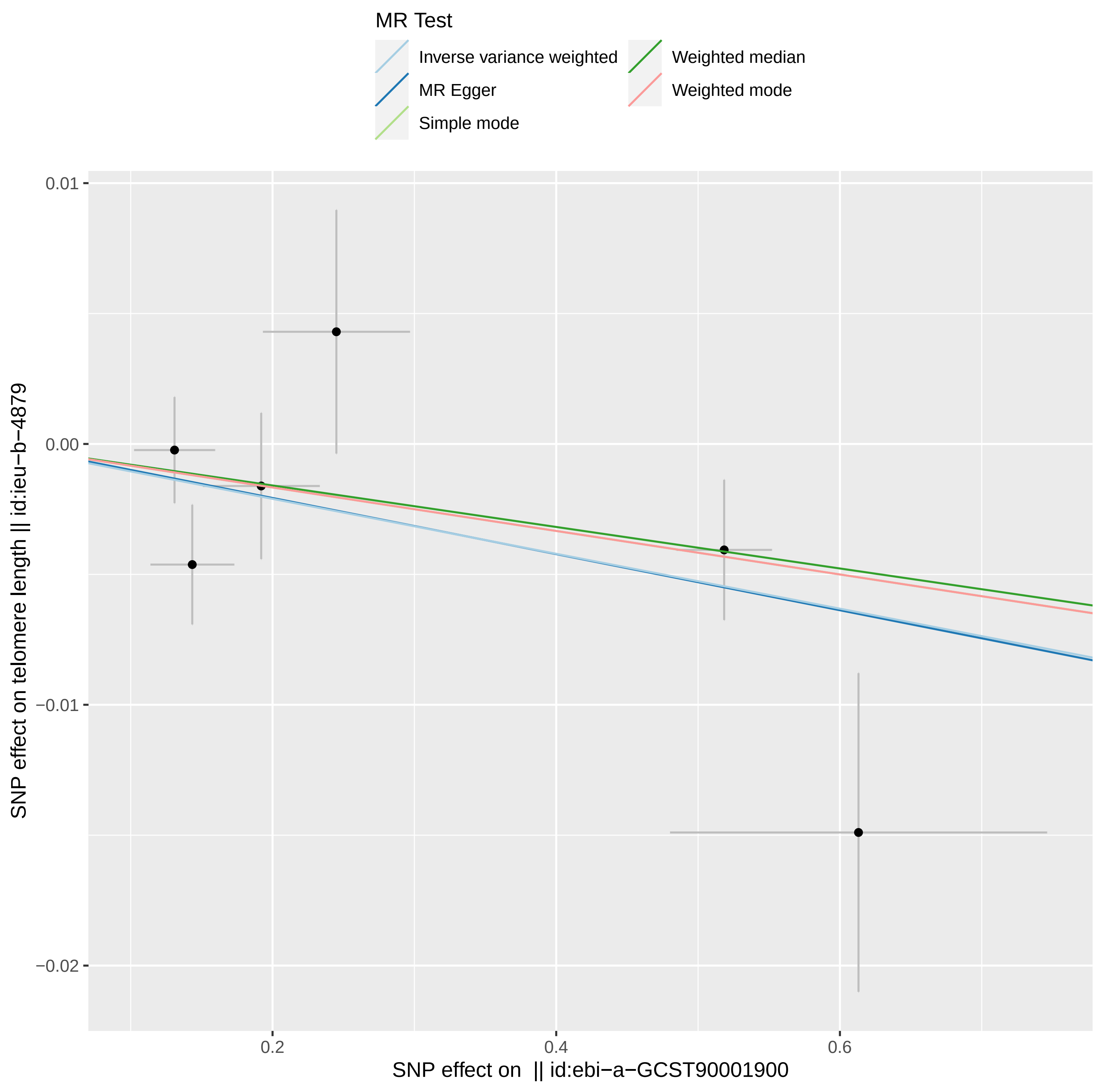
MR Test
Inverse variance weighted
MR Egger
Simple mode
Weighted median
Weighted mode
0.01
0.00
−0.01
−0.02
0.2
0.4
0.6
SNP effect on telomere length || id:ieu−b−4879
SNP effect on || id:ebi−a−GCST90001900

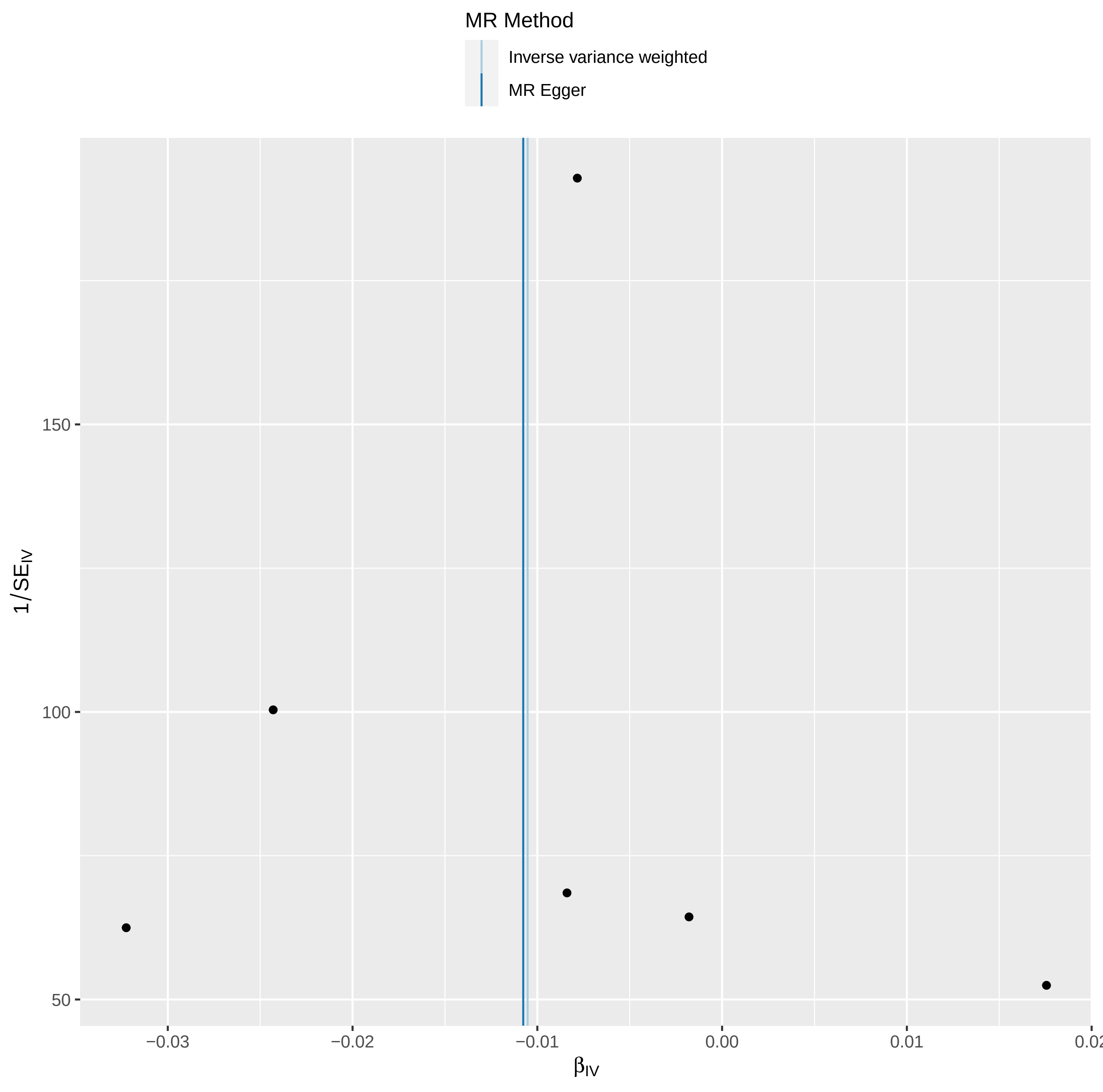
MR Method
Inverse variance weighted
MR Egger
150
100
50
1/SE_IV
−0.03
−0.02
−0.01
0.00
0.01
0.02
β_IV

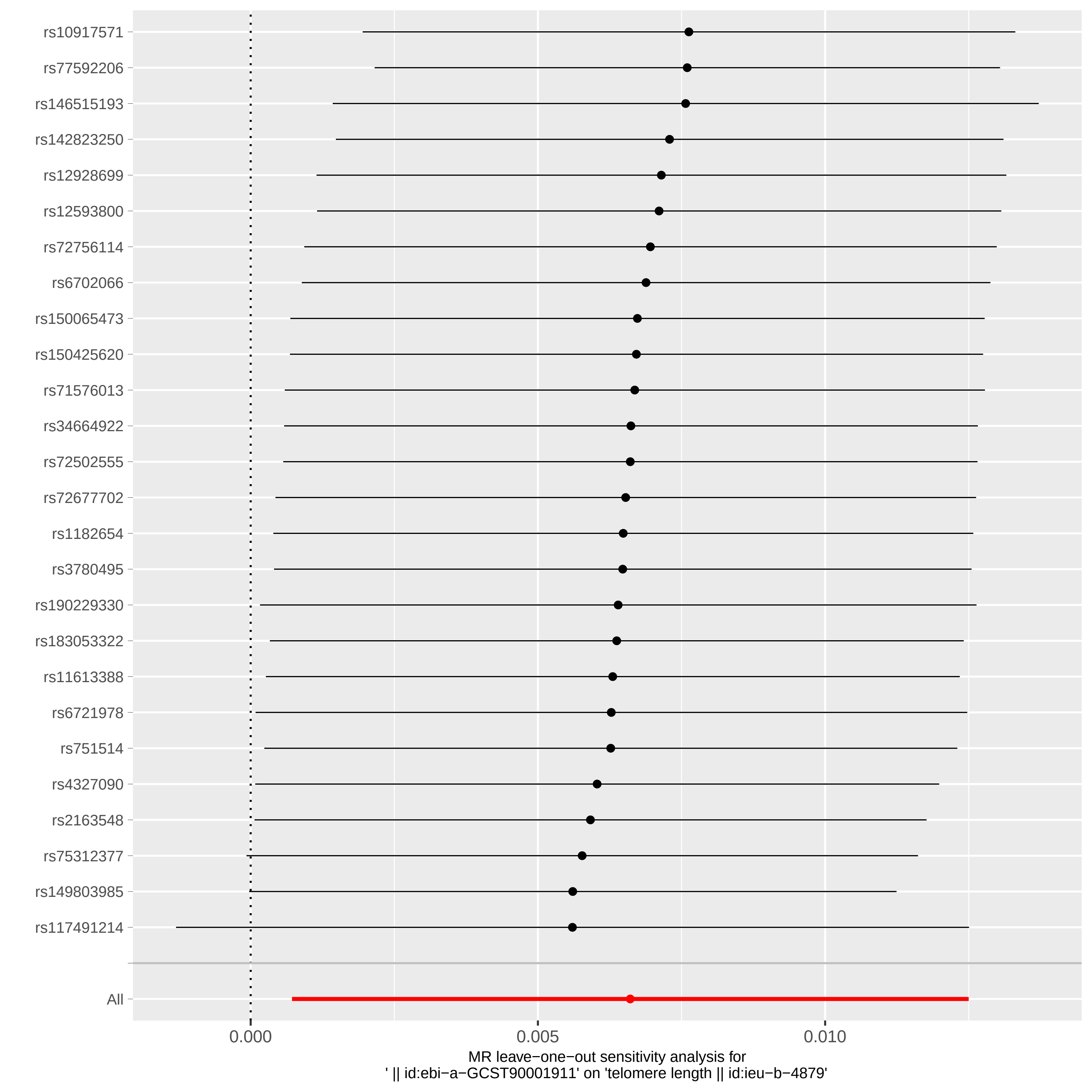
rs10917571
rs77592206
rs146515193
rs142823250
rs12928699
rs12593800
rs72756114
rs6702066
rs150065473
rs150425620
rs71576013
rs34664922
rs72502555
rs72677702
rs1182654
rs3780495
rs190229330
rs183053322
rs11613388
rs6721978
rs751514
rs4327090
rs2163548
rs75312377
rs149803985
rs117491214
All
0.000
0.005
0.010
MR leave−one−out sensitivity analysis for
' || id:ebi−a−GCST90001911' on 'telomere length || id:ieu−b−4879'

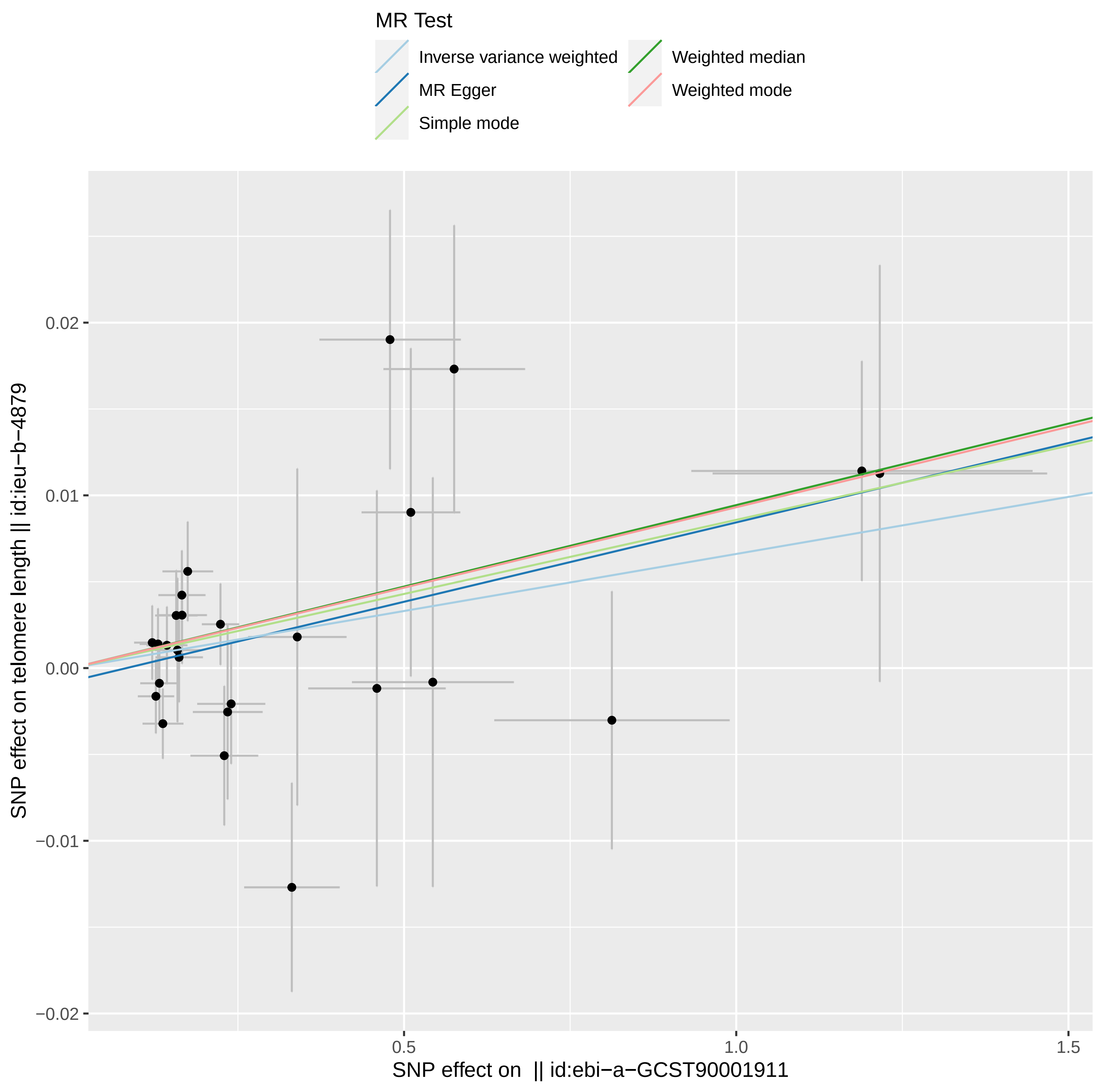
MR Test
Inverse variance weighted
MR Egger
Simple mode
Weighted median
Weighted mode
0.02
0.01
0.00
−0.01
−0.02
SNP effect on telomere length || id:ieu−b−4879
0.5
1.0
1.5
SNP effect on || id:ebi−a−GCST90001911

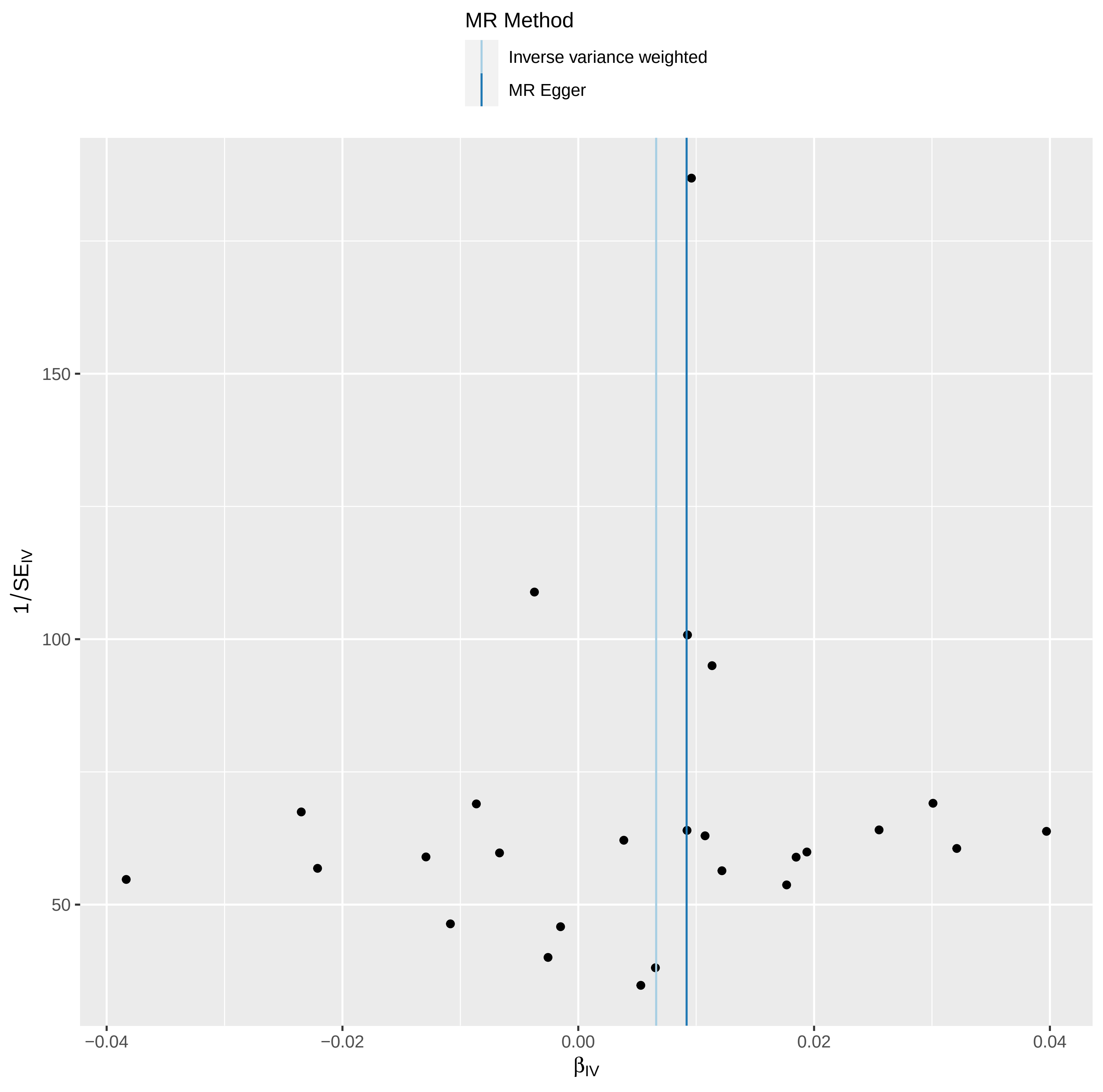
MR Method
Inverse variance weighted
MR Egger
150
100
50
1/SE$_{IV}$
−0.04
−0.02
0.00
0.02
0.04
β$_{IV}$

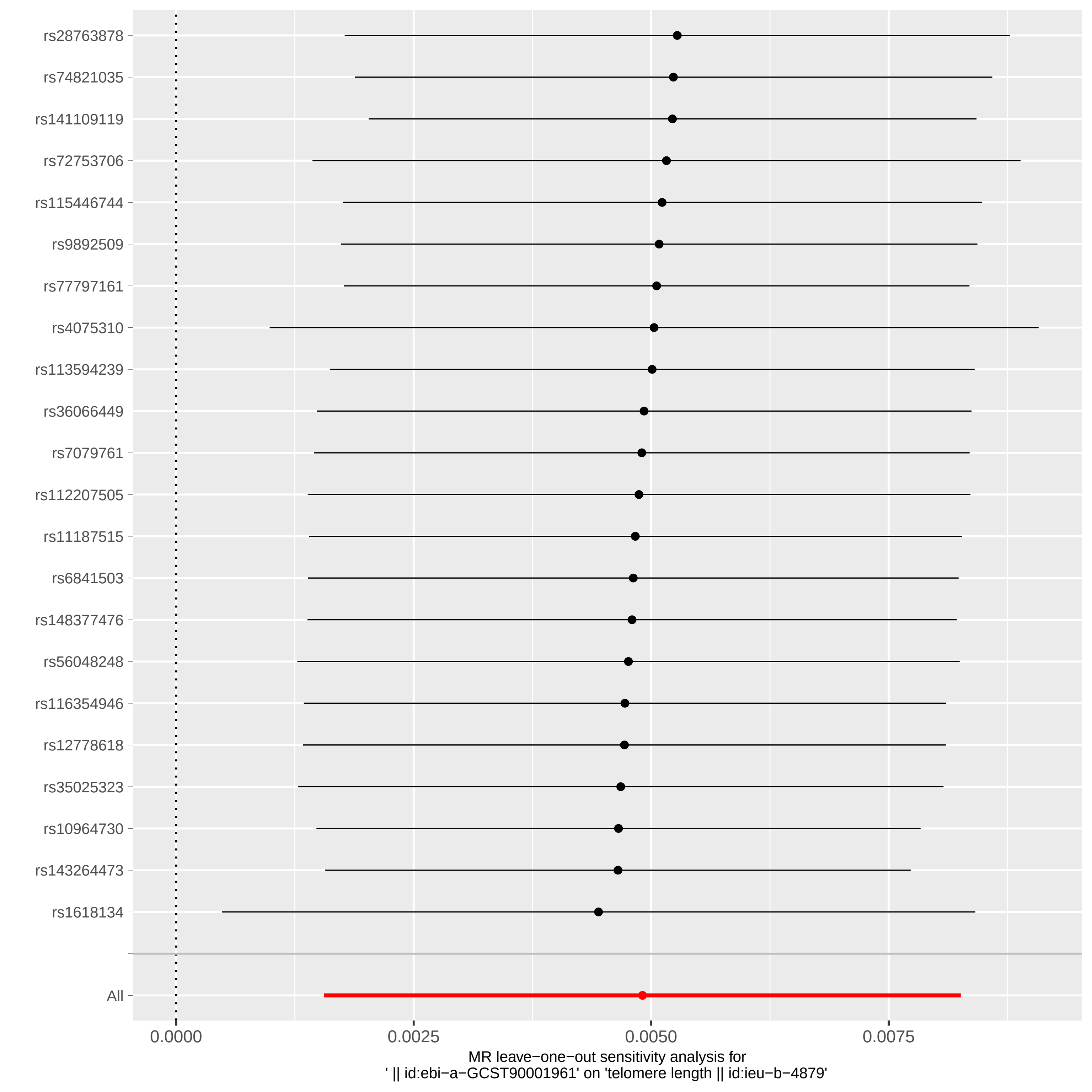

rs28763878
rs74821035
rs141109119
rs72753706
rs115446744
rs9892509
rs77797161
rs4075310
rs113594239
rs36066449
rs7079761
rs112207505
rs11187515
rs6841503
rs148377476
rs56048248
rs116354946
rs12778618
rs35025323
rs10964730
rs143264473
rs1618134
All
0.0000
0.0025
0.0050
0.0075
MR leave−one−out sensitivity analysis for
' || id:ebi−a−GCST90001961' on 'telomere length || id:ieu−b−4879'

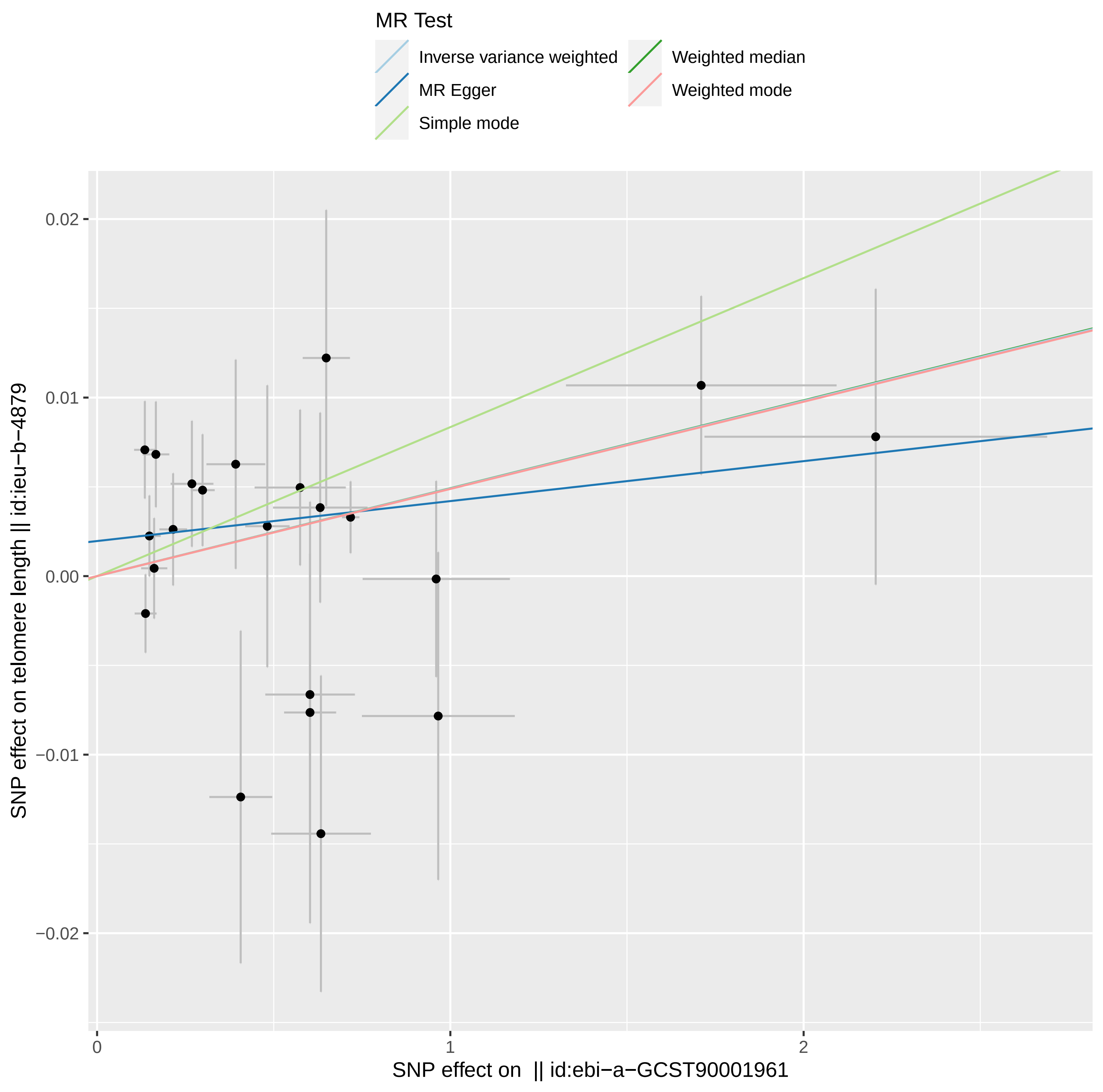
MR Test
Inverse variance weighted
MR Egger
Simple mode
Weighted median
Weighted mode
0.02
0.01
0.00
−0.01
−0.02
0
1
2
SNP effect on telomere length || id:ieu−b−4879
SNP effect on || id:ebi−a−GCST90001961

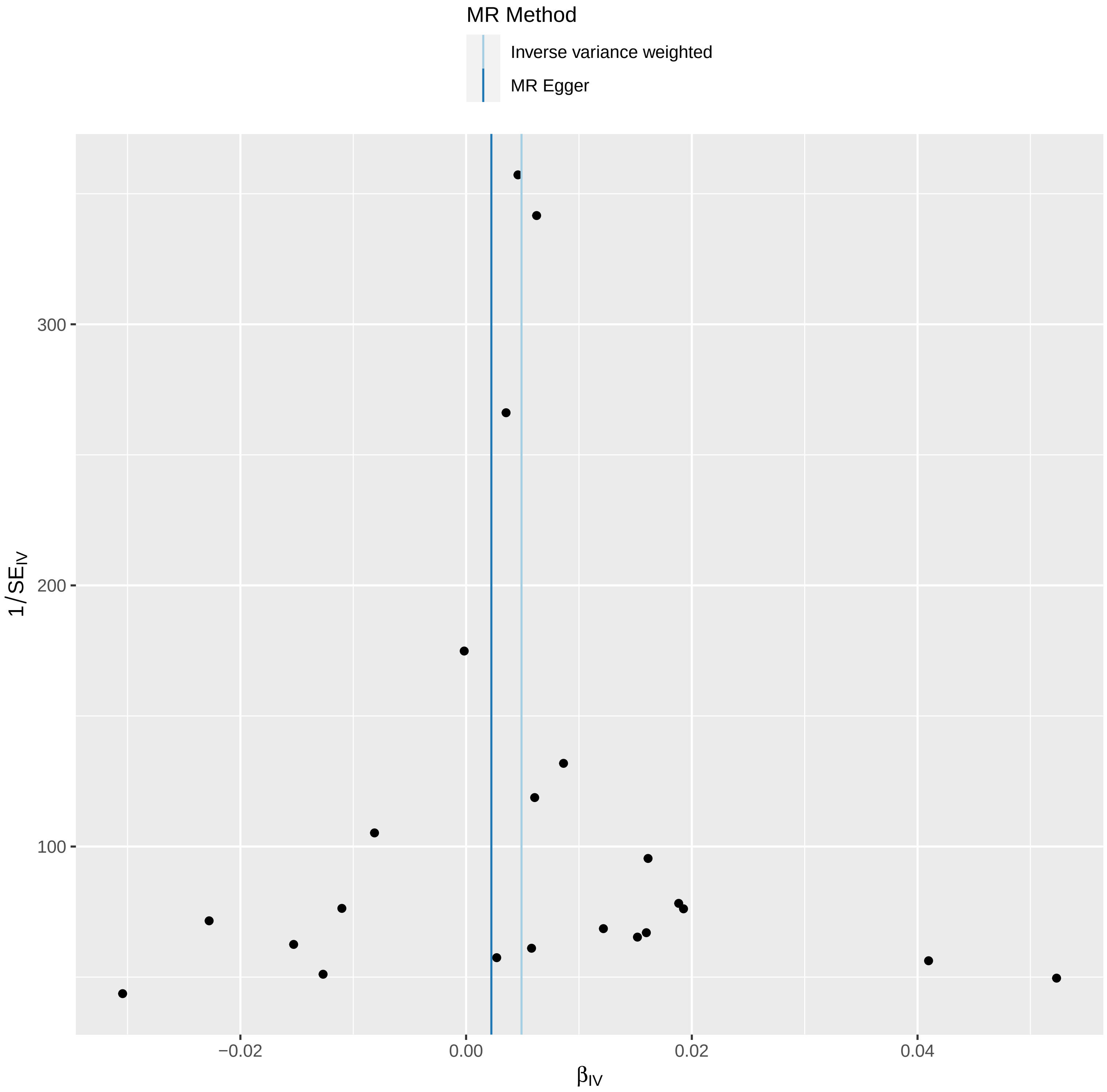
MR Method
Inverse variance weighted
MR Egger
1/SE_IV
300
200
100
−0.02
0.00
0.02
0.04
β_IV

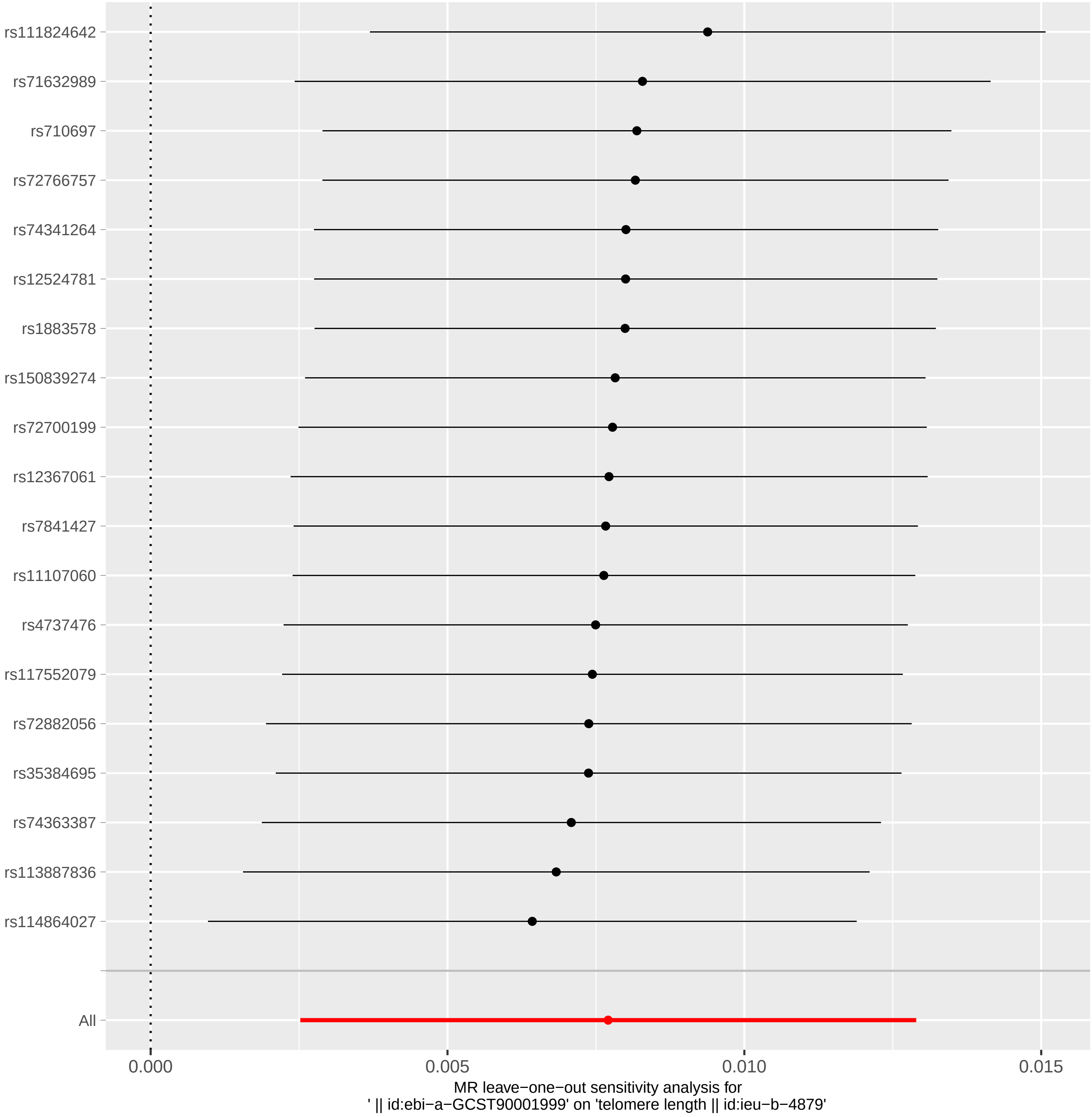
rs111824642
rs71632989
rs710697
rs72766757
rs74341264
rs12524781
rs1883578
rs150839274
rs72700199
rs12367061
rs7841427
rs11107060
rs4737476
rs117552079
rs72882056
rs35384695
rs74363387
rs113887836
rs114864027
All
0.000
0.005
0.010
0.015
MR leave−one−out sensitivity analysis for
' || id:ebi−a−GCST90001999' on 'telomere length || id:ieu−b−4879'

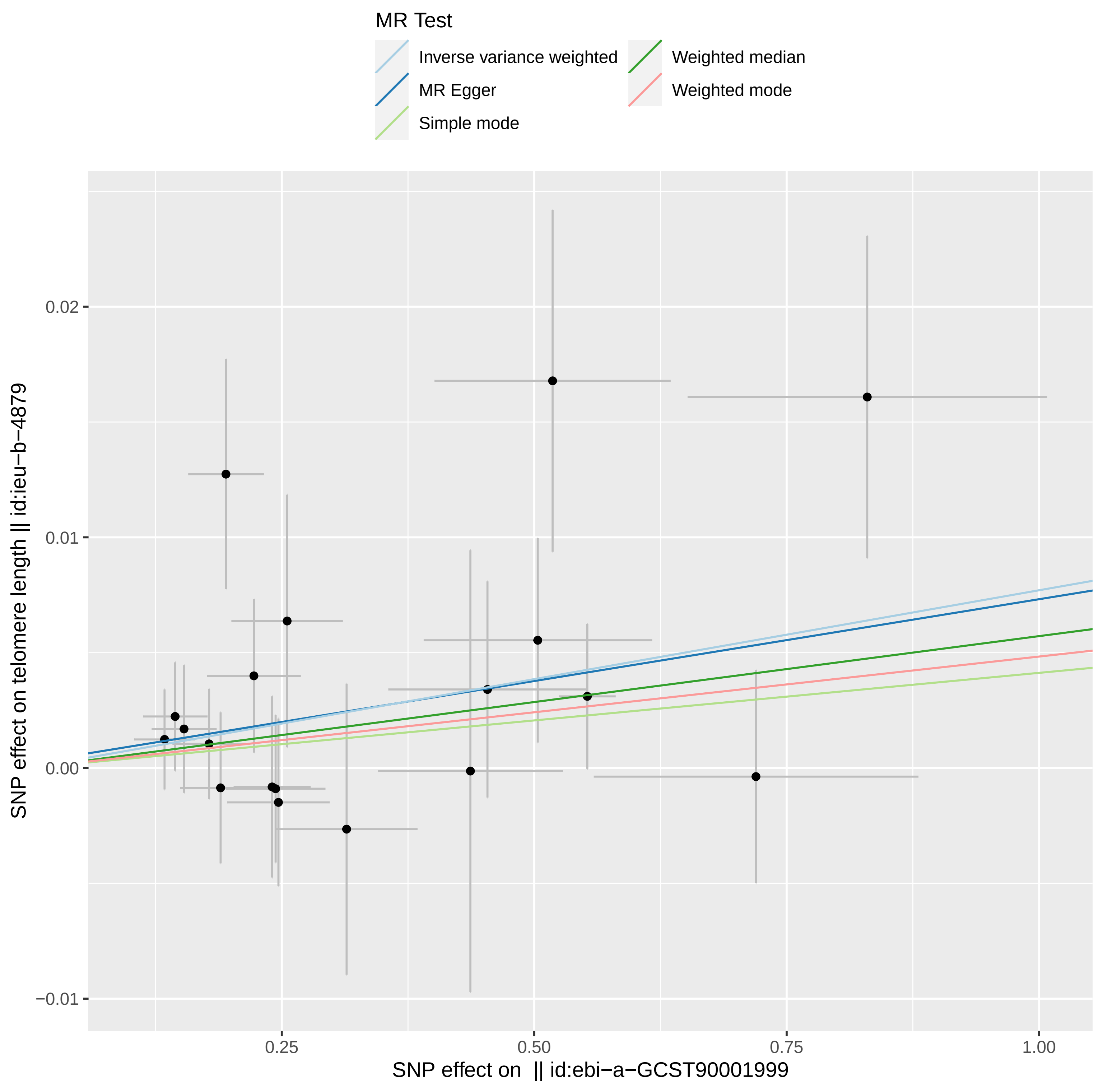
MR Test
Inverse variance weighted
MR Egger
Simple mode
Weighted median
Weighted mode
0.02
0.01
0.00
−0.01
0.25
0.50
0.75
1.00
SNP effect on telomere length || id:ieu−b−4879
SNP effect on || id:ebi−a−GCST90001999

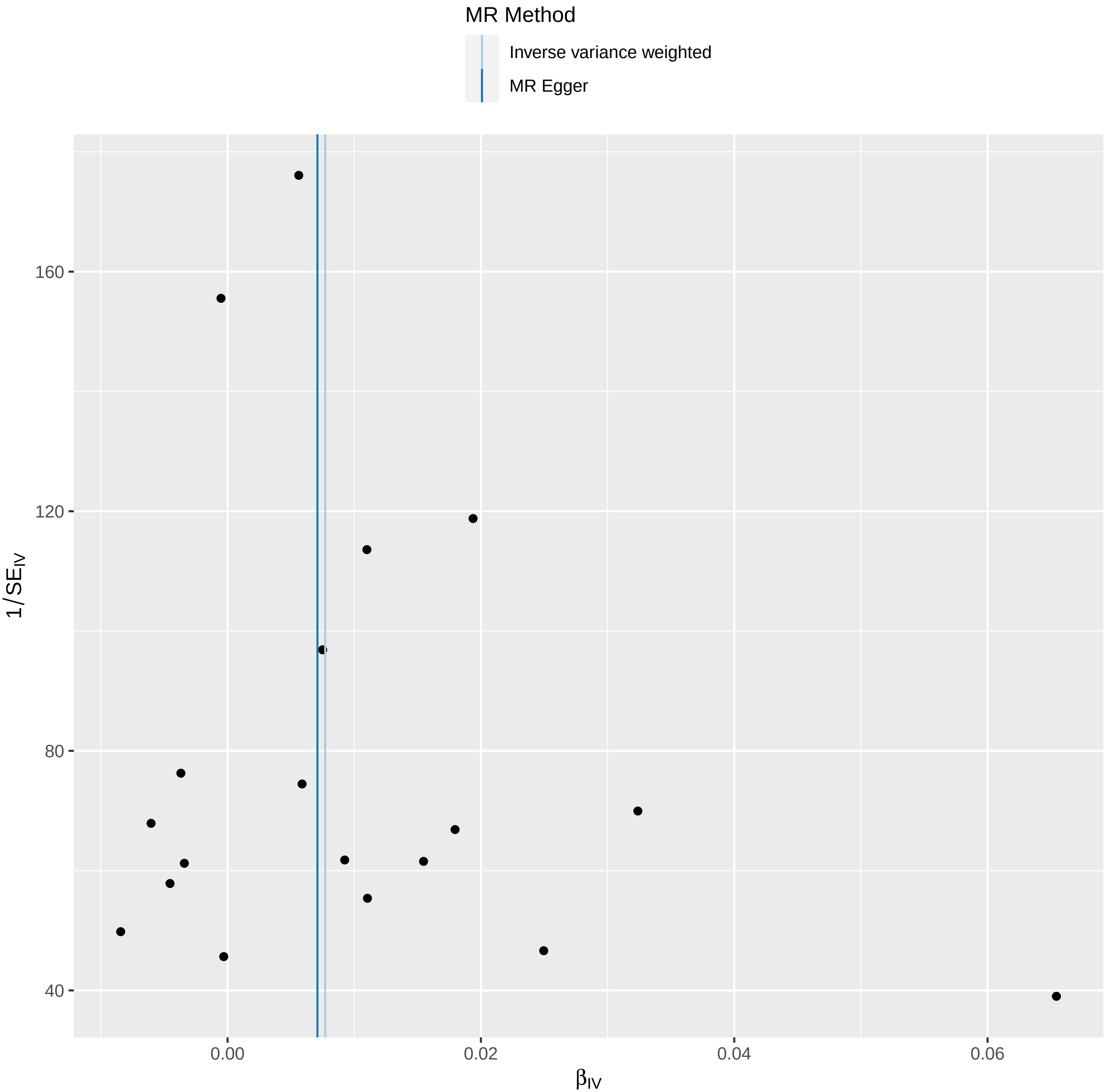

MR Method
Inverse variance weighted
MR Egger
160
120
80
40
1/SE_IV
0.00
0.02
0.04
0.06
β_IV
