# Supplementary material for: Causal relationship between immune cells and telomere length: mendelian randomization analysis
Source: BMC Immunol. 2024 Mar 8;25:19. doi: 10.1186/s12865-024-00610-6 (PMC10924351; doi:10.1186/s12865-024-00610-6)
Supplement: Supplementary file 4 — Supplementary Material 4 [file 12865_2024_610_MOESM4_ESM.pdf]

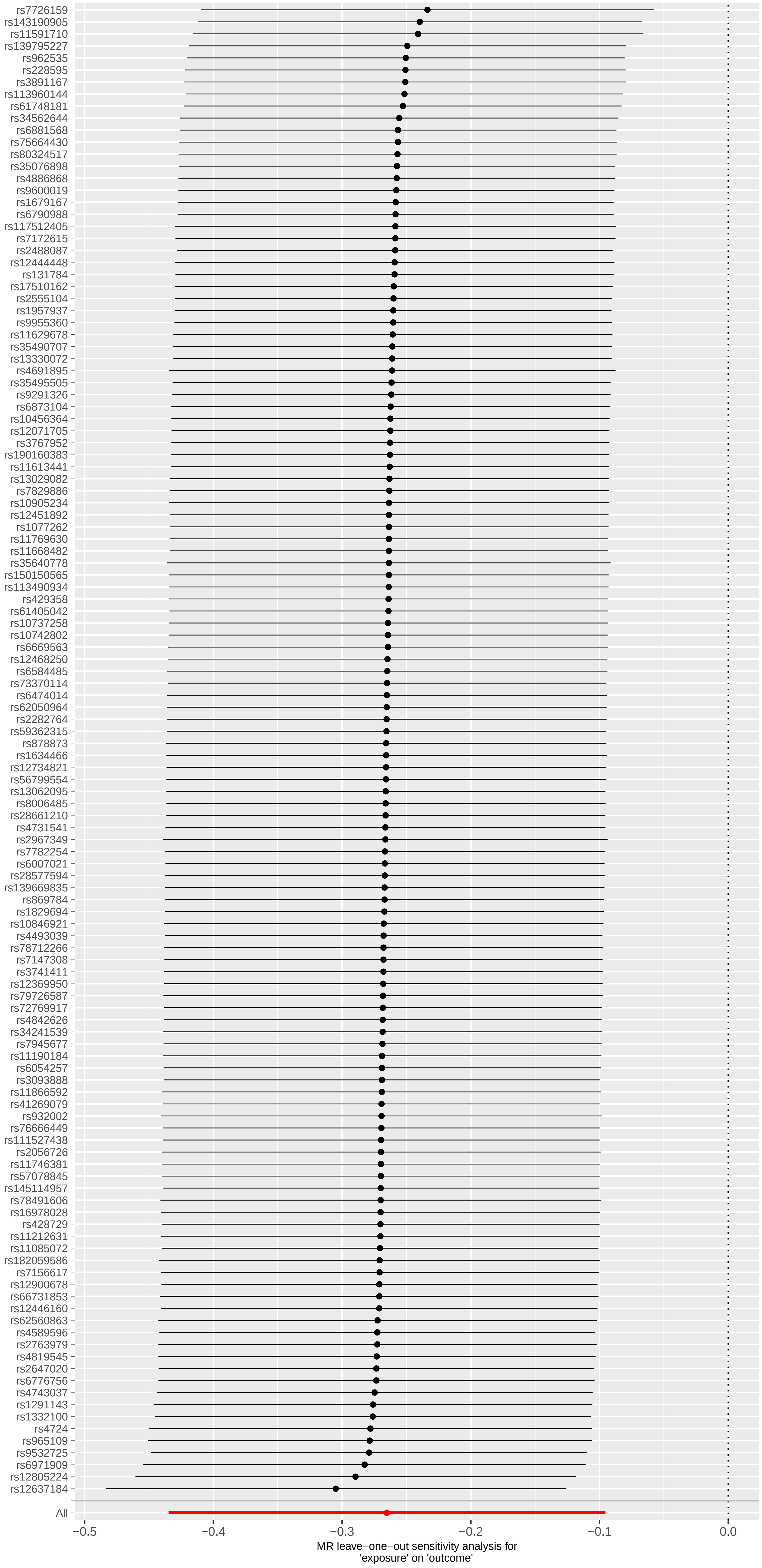
rs7726159
rs143190905
rs11591710
rs139795227
rs962535
rs228595
rs3891167
rs113960144
rs61748181
rs34562644
rs6881568
rs75664430
rs80324517
rs35076898
rs4886868
rs9600019
rs1679167
rs6790988
rs117512405
rs7172615
rs2488087
rs12444448
rs131784
rs17510162
rs2555104
rs1957937
rs9955360
rs11629678
rs35490707
rs13330072
rs4691895
rs35495505
rs9291326
rs6873104
rs10456364
rs12071705
rs3767952
rs190160383
rs11613441
rs13029082
rs7829886
rs10905234
rs12451892
rs1077262
rs11769630
rs11668482
rs35640778
rs150150565
rs113490934
rs429358
rs61405042
rs10737258
rs10742802
rs6669563
rs12468250
rs6584485
rs73370114
rs6474014
rs62050964
rs2282764
rs59362315
rs878873
rs1634466
rs12734821
rs56799554
rs13062095
rs8006485
rs28661210
rs4731541
rs2967349
rs7782254
rs6007021
rs28577594
rs139669835
rs869784
rs1829694
rs10846921
rs4493039
rs78712266
rs7147308
rs3741411
rs12369950
rs79726587
rs72769917
rs4842626
rs34241539
rs7945677
rs11190184
rs6054257
rs3093888
rs11866592
rs41269079
rs932002
rs76666449
rs111527438
rs2056726
rs11746381
rs57078845
rs145114957
rs78491606
rs16978028
rs428729
rs11212631
rs11085072
rs182059586
rs7156617
rs12900678
rs66731853
rs12446160
rs62560863
rs4589596
rs2763979
rs4819545
rs2647020
rs6776756
rs4743037
rs1291143
rs1332100
rs4724
rs965109
rs9532725
rs6971909
rs12805224
rs12637184
All
−0.5
−0.4
−0.3
−0.2
−0.1
0.0
MR leave−one−out sensitivity analysis for
'exposure' on 'outcome'

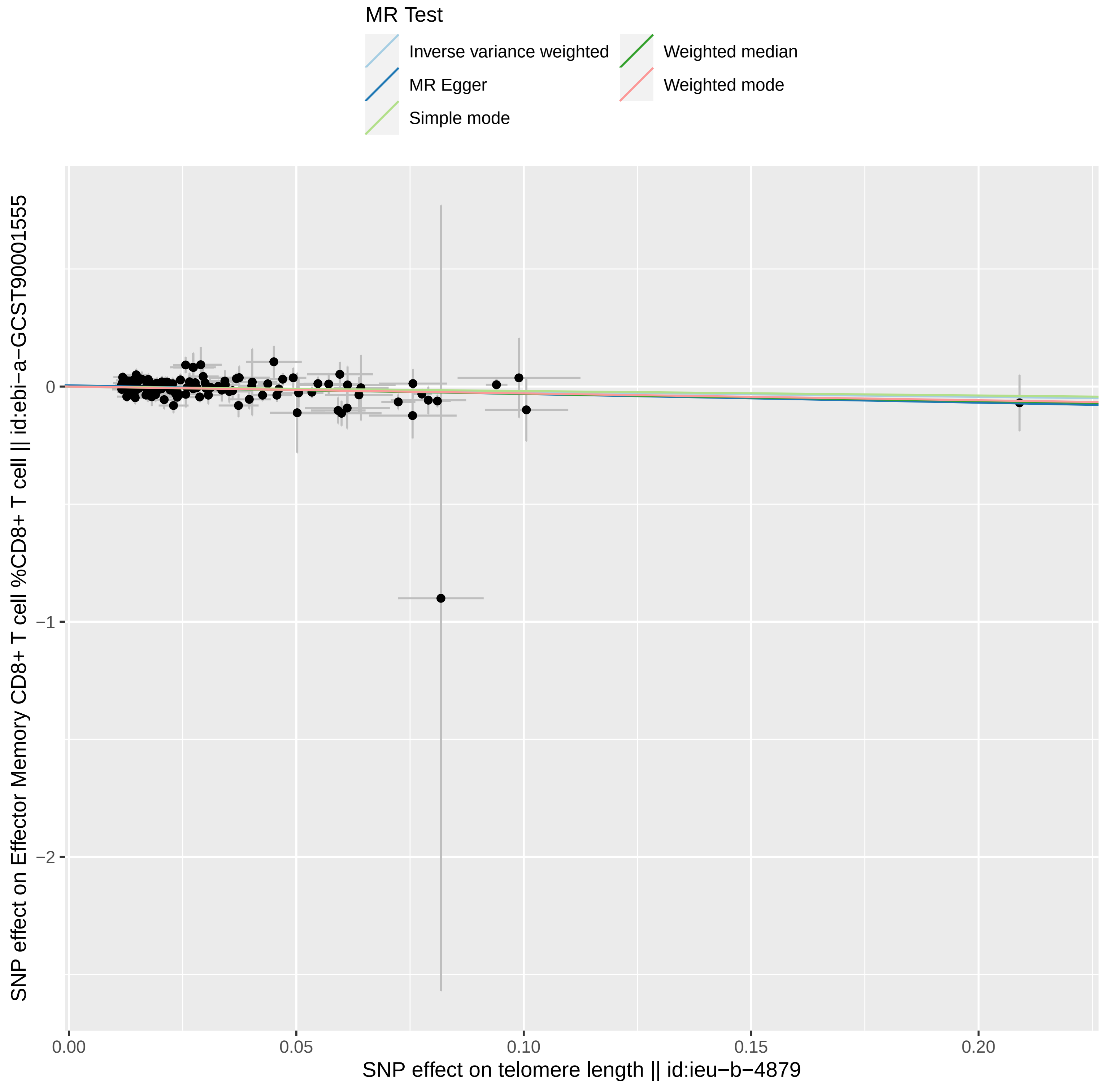
MR Test
Inverse variance weighted
MR Egger
Simple mode
Weighted median
Weighted mode
0
−1
−2
0.00
0.05
0.10
0.15
0.20
SNP effect on telomere length || id:ieu−b−4879
SNP effect on Effector Memory CD8+ T cell %CD8+ T cell || id:ebi−a−GCST90001555

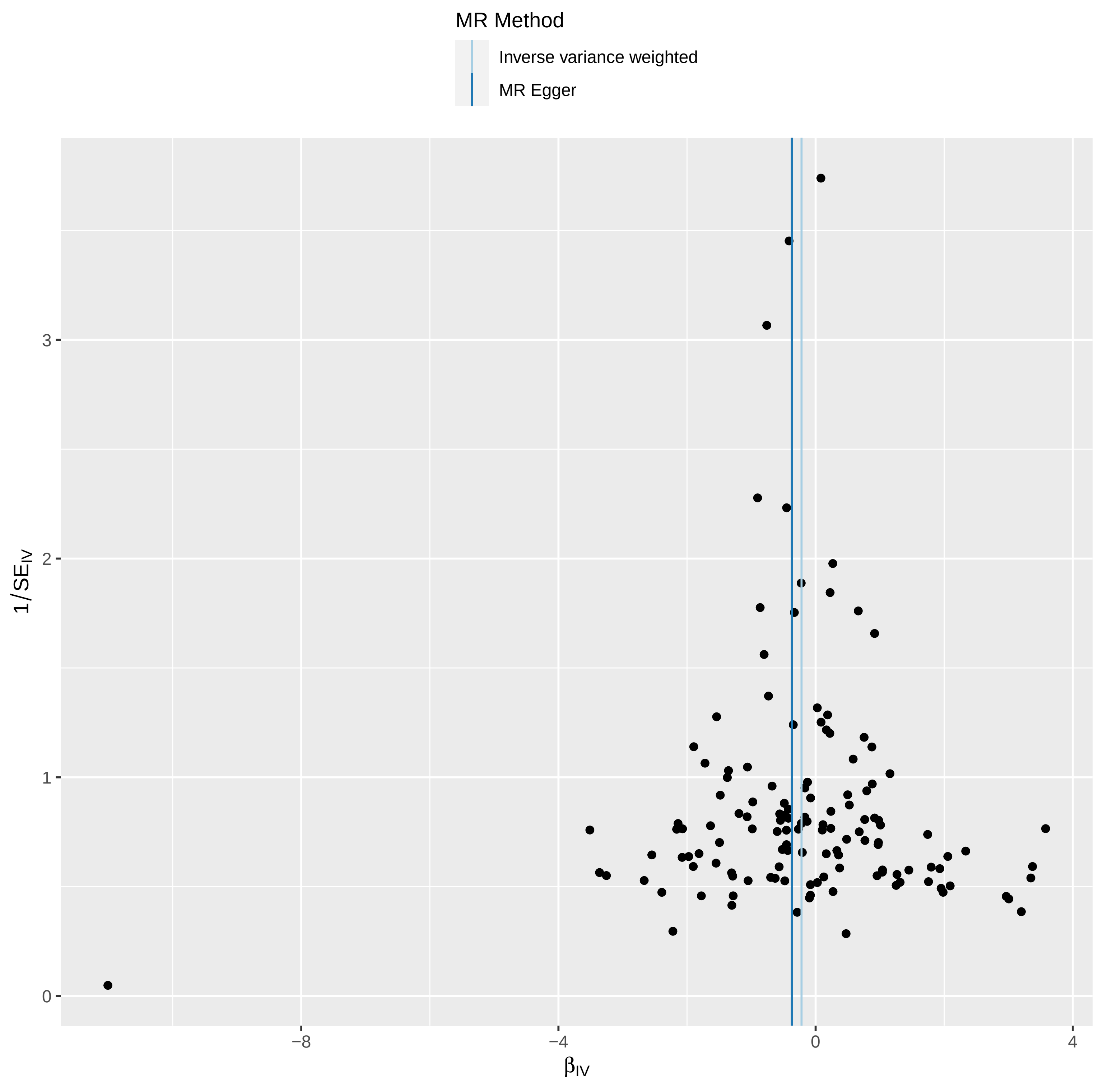
MR Method
Inverse variance weighted
MR Egger
1/SE_IV
3
2
1
0
β_IV
−8
−4
0
4

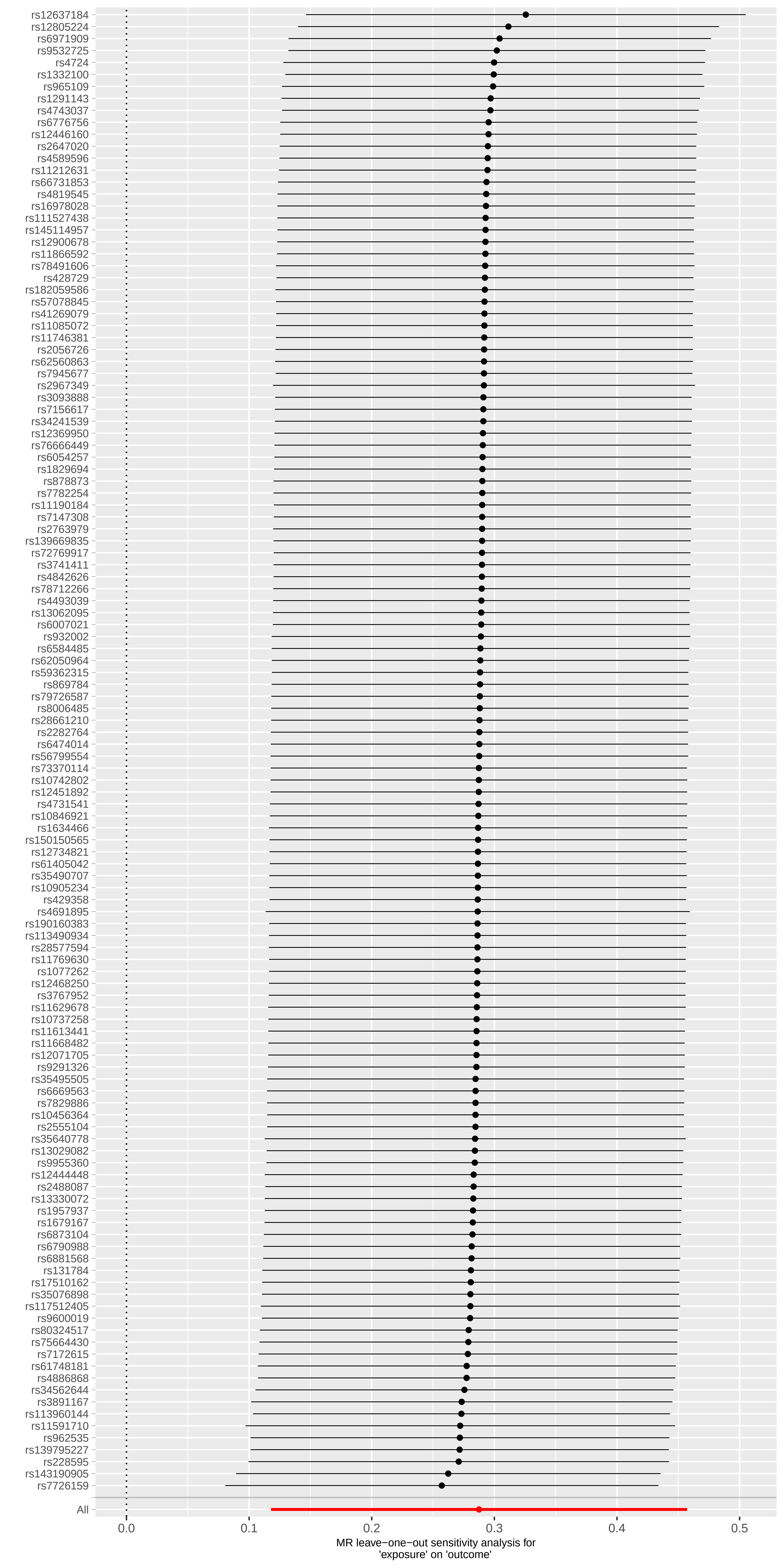

rs12637184
rs12805224
rs6971909
rs9532725
rs4724
rs1332100
rs965109
rs1291143
rs4743037
rs6776756
rs12446160
rs2647020
rs4589596
rs11212631
rs66731853
rs4819545
rs16978028
rs111527438
rs145114957
rs12900678
rs11866592
rs78491606
rs428729
rs182059586
rs57078845
rs41269079
rs11085072
rs11746381
rs2056726
rs62560863
rs7945677
rs2967349
rs3093888
rs7156617
rs34241539
rs12369950
rs76666449
rs6054257
rs1829694
rs878873
rs7782254
rs11190184
rs7147308
rs2763979
rs139669835
rs72769917
rs3741411
rs4842626
rs78712266
rs4493039
rs13062095
rs6007021
rs932002
rs6584485
rs62050964
rs59362315
rs869784
rs79726587
rs8006485
rs28661210
rs2282764
rs6474014
rs56799554
rs73370114
rs10742802
rs12451892
rs4731541
rs10846921
rs1634466
rs150150565
rs12734821
rs61405042
rs35490707
rs10905234
rs429358
rs4691895
rs190160383
rs113490934
rs28577594
rs11769630
rs1077262
rs12468250
rs3767952
rs11629678
rs10737258
rs11613441
rs11668482
rs12071705
rs9291326
rs35495505
rs6669563
rs7829886
rs10456364
rs2555104
rs35640778
rs13029082
rs9955360
rs12444448
rs2488087
rs13330072
rs1957937
rs1679167
rs6873104
rs6790988
rs6881568
rs131784
rs17510162
rs35076898
rs117512405
rs9600019
rs80324517
rs75664430
rs7172615
rs61748181
rs4886868
rs34562644
rs3891167
rs113960144
rs11591710
rs962535
rs139795227
rs228595
rs143190905
rs7726159
All
0.0
0.1
0.2
0.3
0.4
0.5
MR leave−one−out sensitivity analysis for
'exposure' on 'outcome'

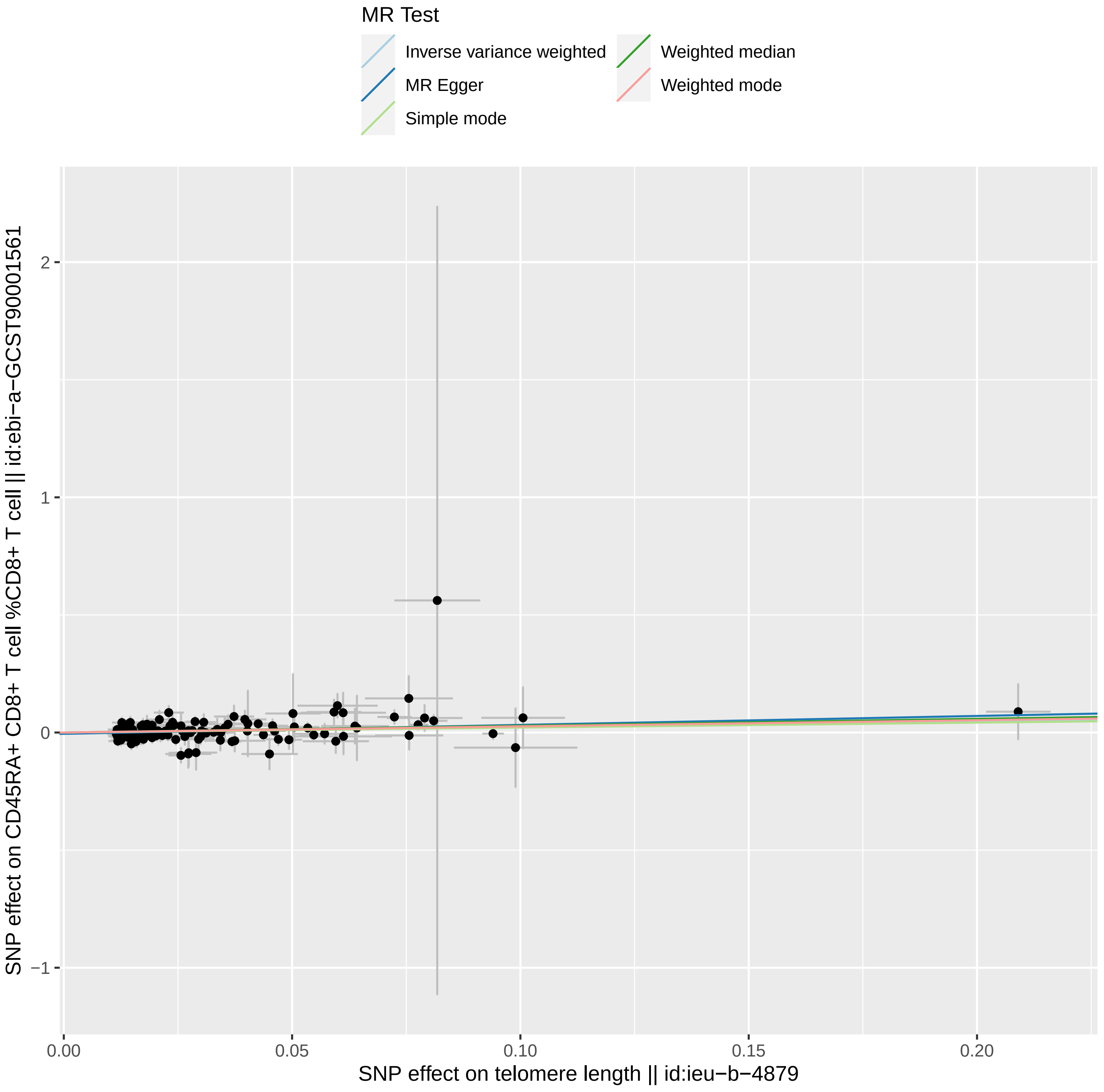
MR Test
Inverse variance weighted
MR Egger
Simple mode
Weighted median
Weighted mode
2
1
0
−1
0.00
0.05
0.10
0.15
0.20
SNP effect on CD45RA+ CD8+ T cell %CD8+ T cell || id:ebi−a−GCST90001561
SNP effect on telomere length || id:ieu−b−4879

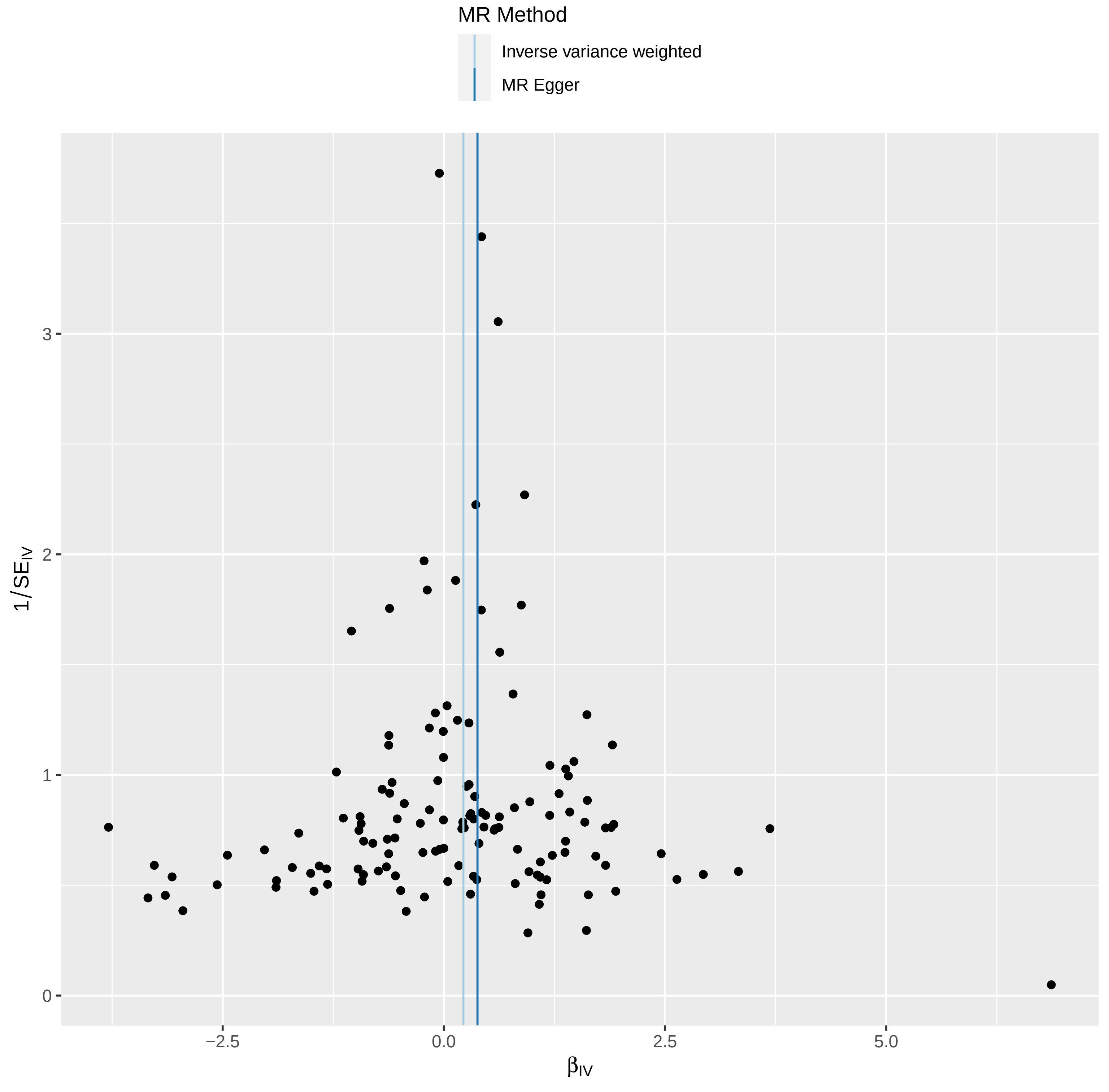

MR Method
Inverse variance weighted
MR Egger
3
2
1
0
1/SE_IV
−2.5
0.0
2.5
5.0
β_IV

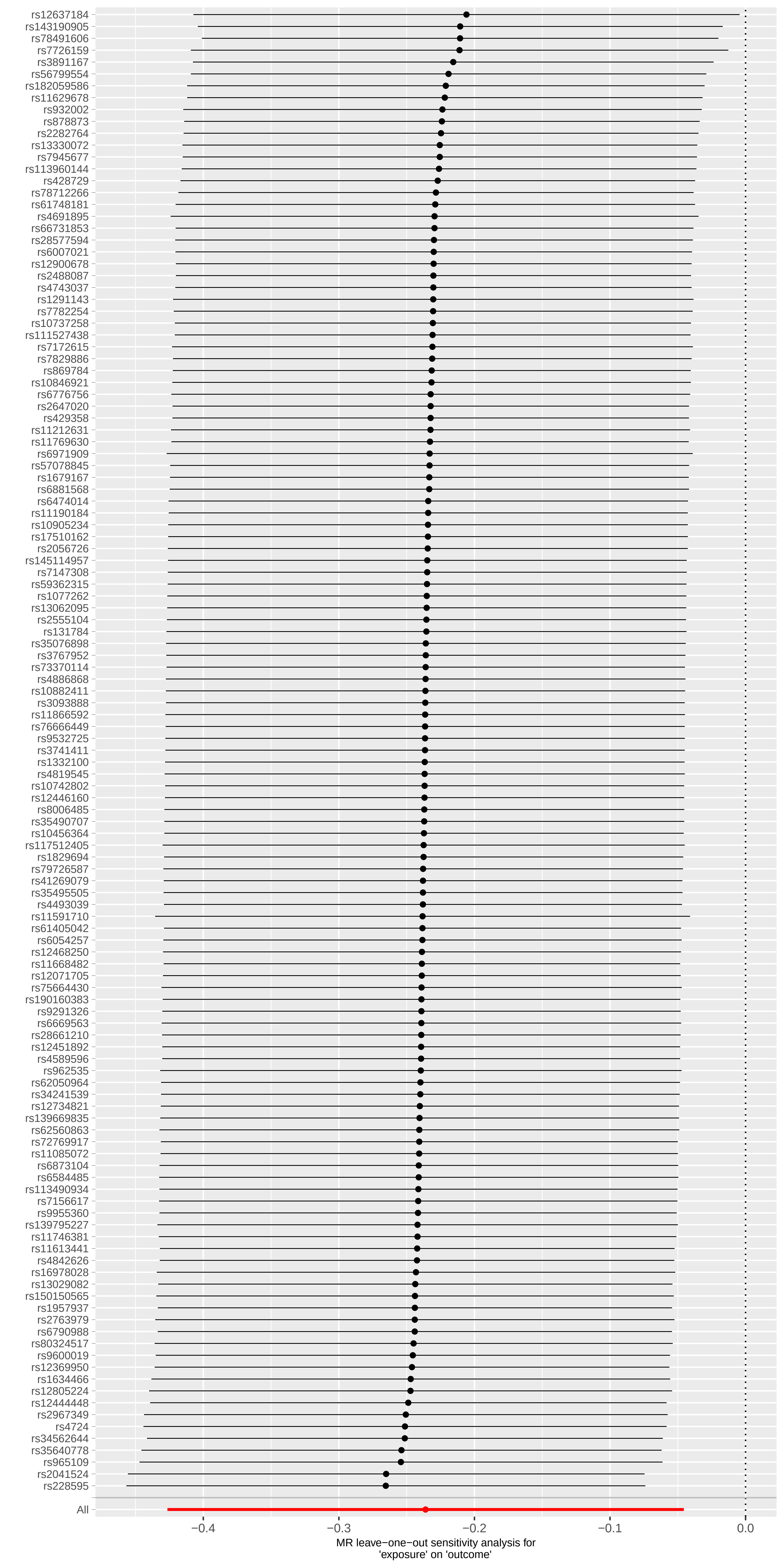
rs12637184
rs143190905
rs78491606
rs7726159
rs3891167
rs56799554
rs182059586
rs11629678
rs932002
rs878873
rs2282764
rs13330072
rs7945677
rs113960144
rs428729
rs78712266
rs61748181
rs4691895
rs66731853
rs28577594
rs6007021
rs12900678
rs2488087
rs4743037
rs1291143
rs7782254
rs10737258
rs111527438
rs7172615
rs7829886
rs869784
rs10846921
rs6776756
rs2647020
rs429358
rs11212631
rs11769630
rs6971909
rs57078845
rs1679167
rs6881568
rs6474014
rs11190184
rs10905234
rs17510162
rs2056726
rs145114957
rs7147308
rs59362315
rs1077262
rs13062095
rs2555104
rs131784
rs35076898
rs3767952
rs73370114
rs4886868
rs10882411
rs3093888
rs11866592
rs76666449
rs9532725
rs3741411
rs1332100
rs4819545
rs10742802
rs12446160
rs8006485
rs35490707
rs10456364
rs117512405
rs1829694
rs79726587
rs41269079
rs35495505
rs4493039
rs11591710
rs61405042
rs6054257
rs12468250
rs11668482
rs12071705
rs75664430
rs190160383
rs9291326
rs6669563
rs28661210
rs12451892
rs4589596
rs962535
rs62050964
rs34241539
rs12734821
rs139669835
rs62560863
rs72769917
rs11085072
rs6873104
rs6584485
rs113490934
rs7156617
rs9955360
rs139795227
rs11746381
rs11613441
rs4842626
rs16978028
rs13029082
rs150150565
rs1957937
rs2763979
rs6790988
rs80324517
rs9600019
rs12369950
rs1634466
rs12805224
rs12444448
rs2967349
rs4724
rs34562644
rs35640778
rs965109
rs2041524
rs228595
All
−0.4
−0.3
−0.2
−0.1
0.0
MR leave−one−out sensitivity analysis for
'exposure' on 'outcome'

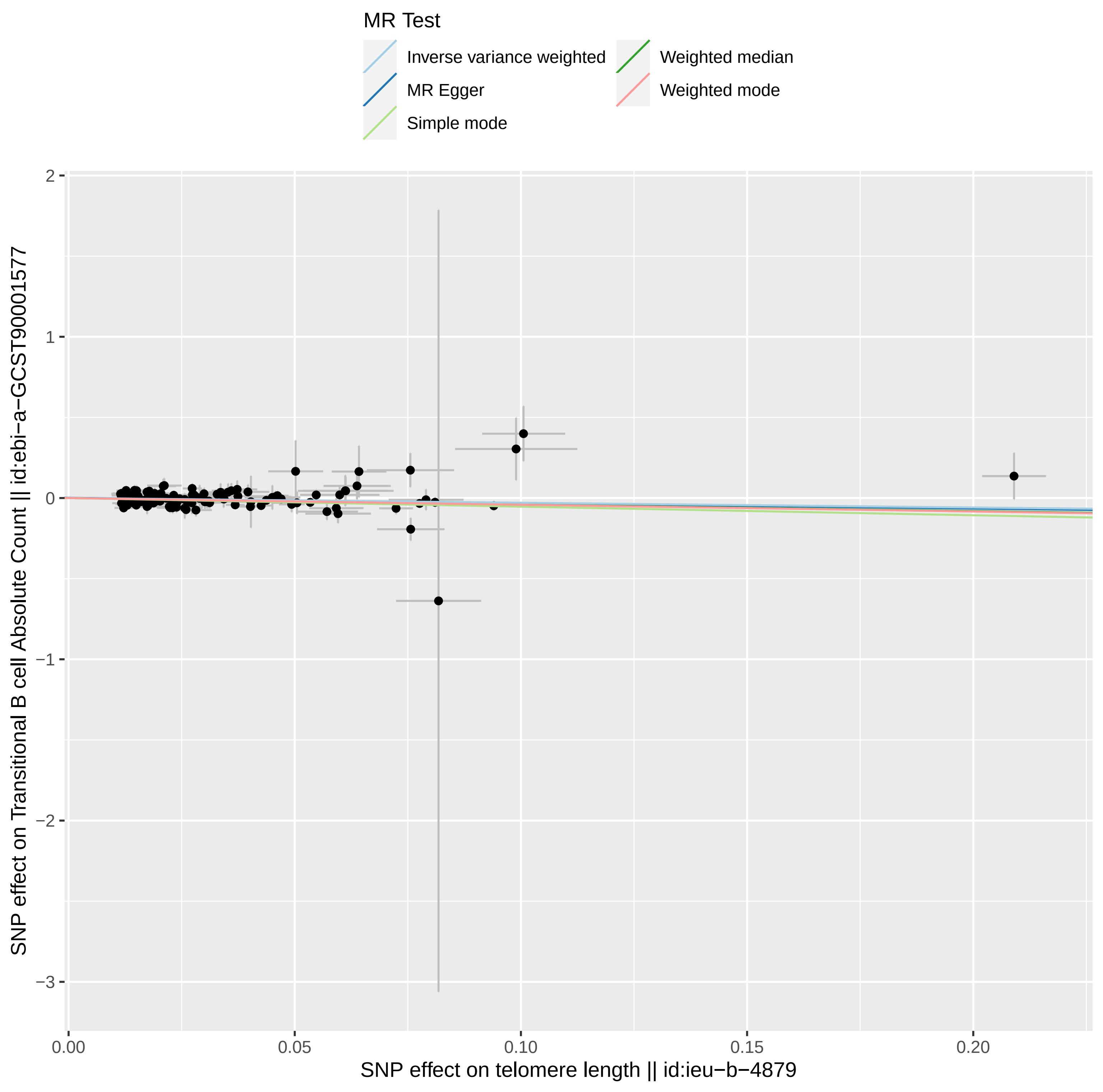
MR Test
Inverse variance weighted
MR Egger
Simple mode
Weighted median
Weighted mode
SNP effect on Transitional B cell Absolute Count || id:ebi-a-GCST90001577
SNP effect on telomere length || id:ieu-b-4879
2
1
0
−1
−2
−3
0.00
0.05
0.10
0.15
0.20

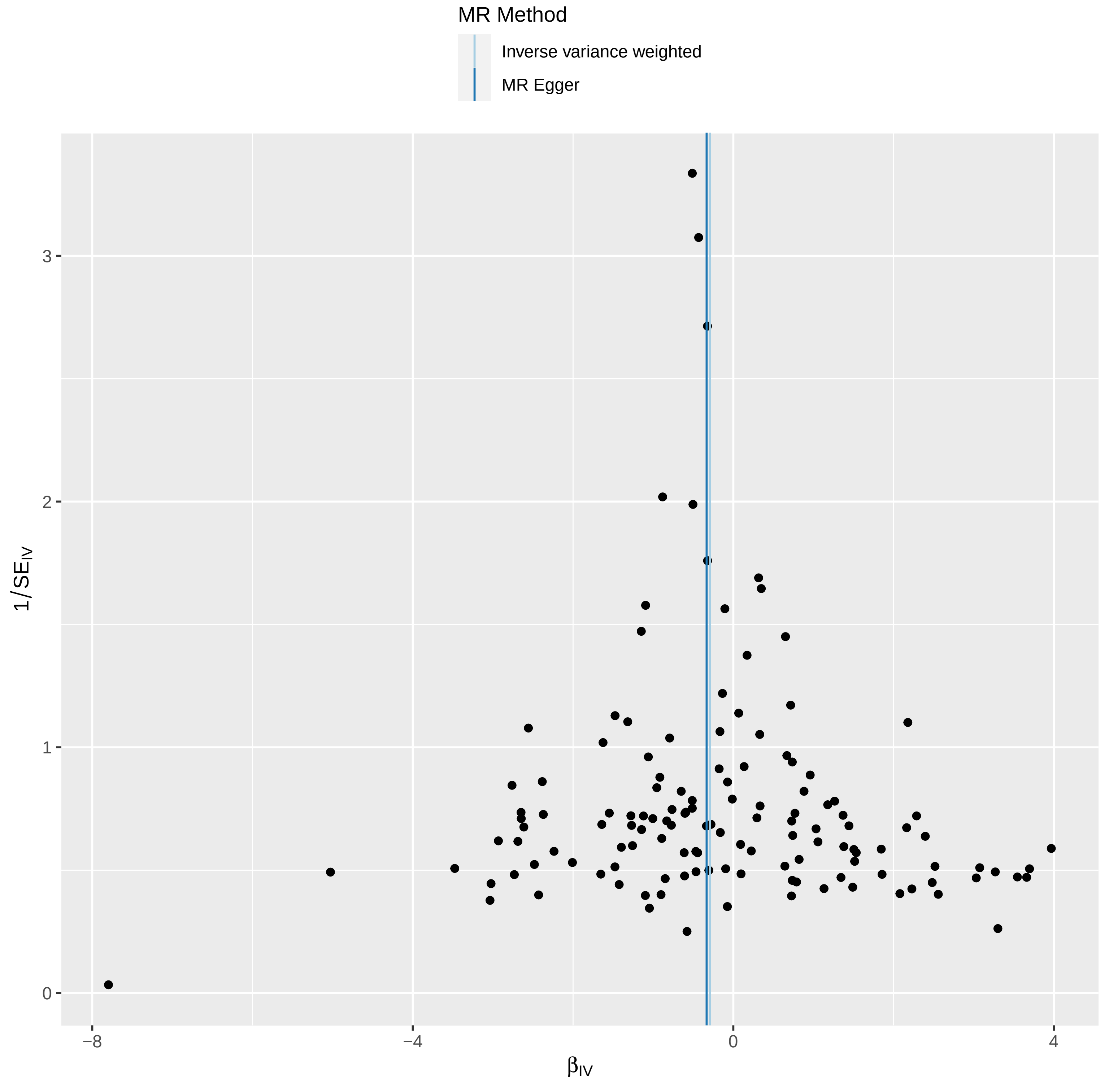

MR Method
Inverse variance weighted
MR Egger
1/SE$_{IV}$
3
2
1
0
−8
−4
0
4
β$_{IV}$

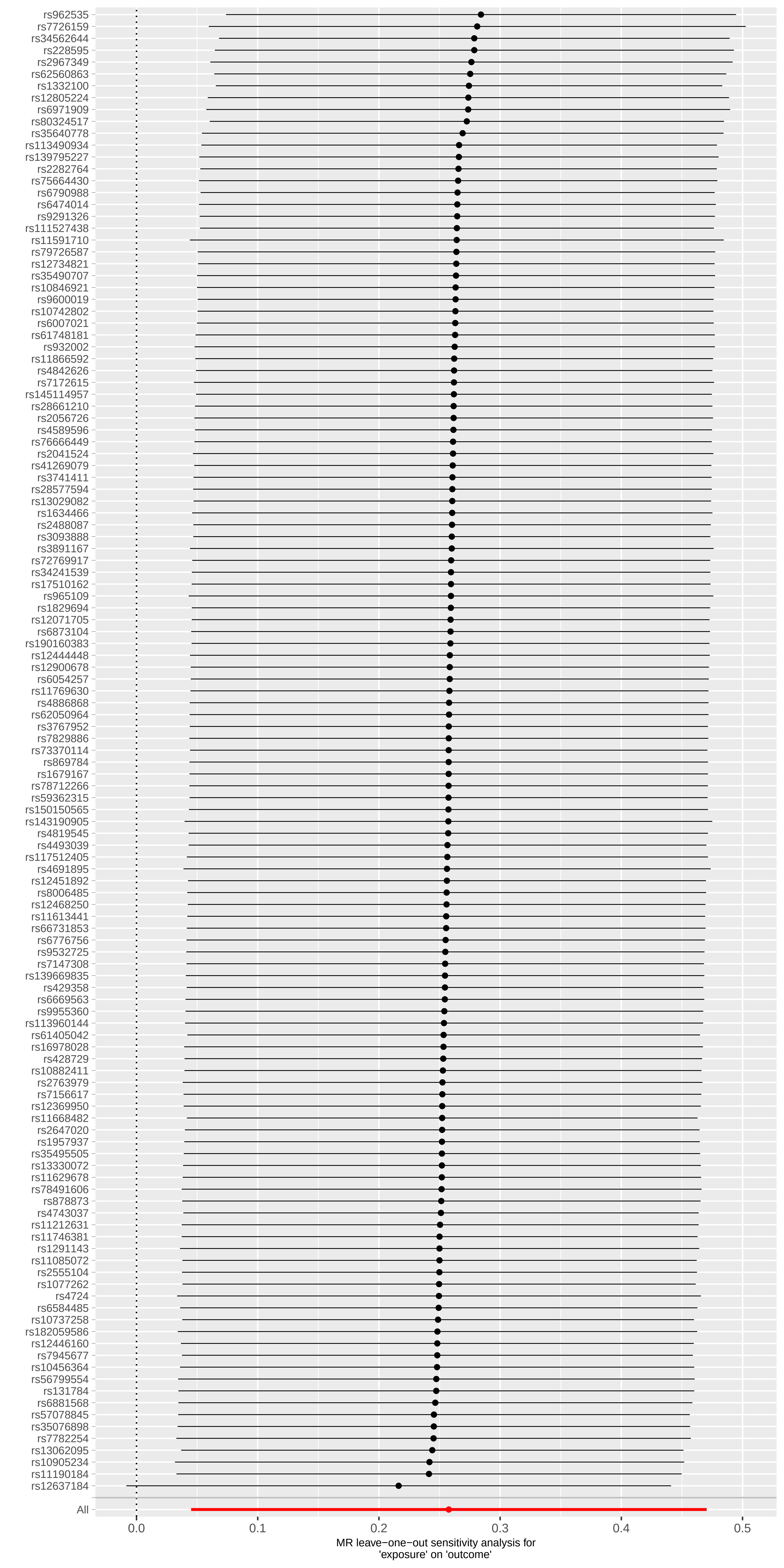
rs962535
rs7726159
rs34562644
rs228595
rs2967349
rs62560863
rs1332100
rs12805224
rs6971909
rs80324517
rs35640778
rs113490934
rs139795227
rs2282764
rs75664430
rs6790988
rs6474014
rs9291326
rs111527438
rs11591710
rs79726587
rs12734821
rs35490707
rs10846921
rs9600019
rs10742802
rs6007021
rs61748181
rs932002
rs11866592
rs4842626
rs7172615
rs145114957
rs28661210
rs2056726
rs4589596
rs76666449
rs2041524
rs41269079
rs3741411
rs28577594
rs13029082
rs1634466
rs2488087
rs3093888
rs3891167
rs72769917
rs34241539
rs17510162
rs965109
rs1829694
rs12071705
rs6873104
rs190160383
rs12444448
rs12900678
rs6054257
rs11769630
rs4886868
rs62050964
rs3767952
rs7829886
rs73370114
rs869784
rs1679167
rs78712266
rs59362315
rs150150565
rs143190905
rs4819545
rs4493039
rs117512405
rs4691895
rs12451892
rs8006485
rs12468250
rs11613441
rs66731853
rs6776756
rs9532725
rs7147308
rs139669835
rs429358
rs6669563
rs9955360
rs113960144
rs61405042
rs16978028
rs428729
rs10882411
rs2763979
rs7156617
rs12369950
rs11668482
rs2647020
rs1957937
rs35495505
rs13330072
rs11629678
rs78491606
rs878873
rs4743037
rs11212631
rs11746381
rs1291143
rs11085072
rs2555104
rs1077262
rs4724
rs6584485
rs10737258
rs182059586
rs12446160
rs7945677
rs10456364
rs56799554
rs131784
rs6881568
rs57078845
rs35076898
rs7782254
rs13062095
rs10905234
rs11190184
rs12637184
All
0.0
0.1
0.2
0.3
0.4
0.5
MR leave−one−out sensitivity analysis for
'exposure' on 'outcome'

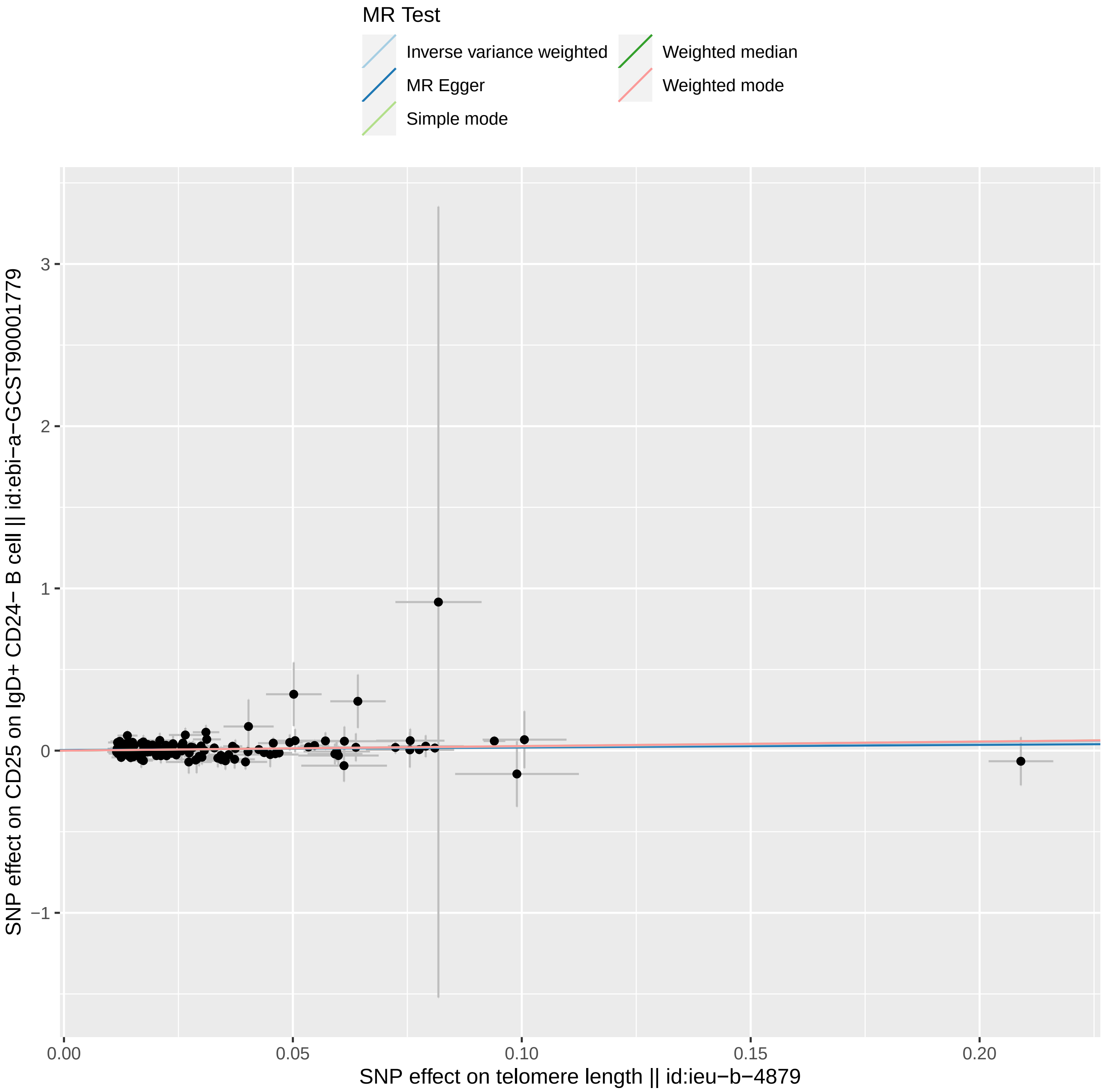

MR Test
Inverse variance weighted
MR Egger
Simple mode
Weighted median
Weighted mode
3
2
1
0
−1
0.00
0.05
0.10
0.15
0.20
SNP effect on CD25 on IgD+ CD24− B cell || id:ebi−a−GCST90001779
SNP effect on telomere length || id:ieu−b−4879

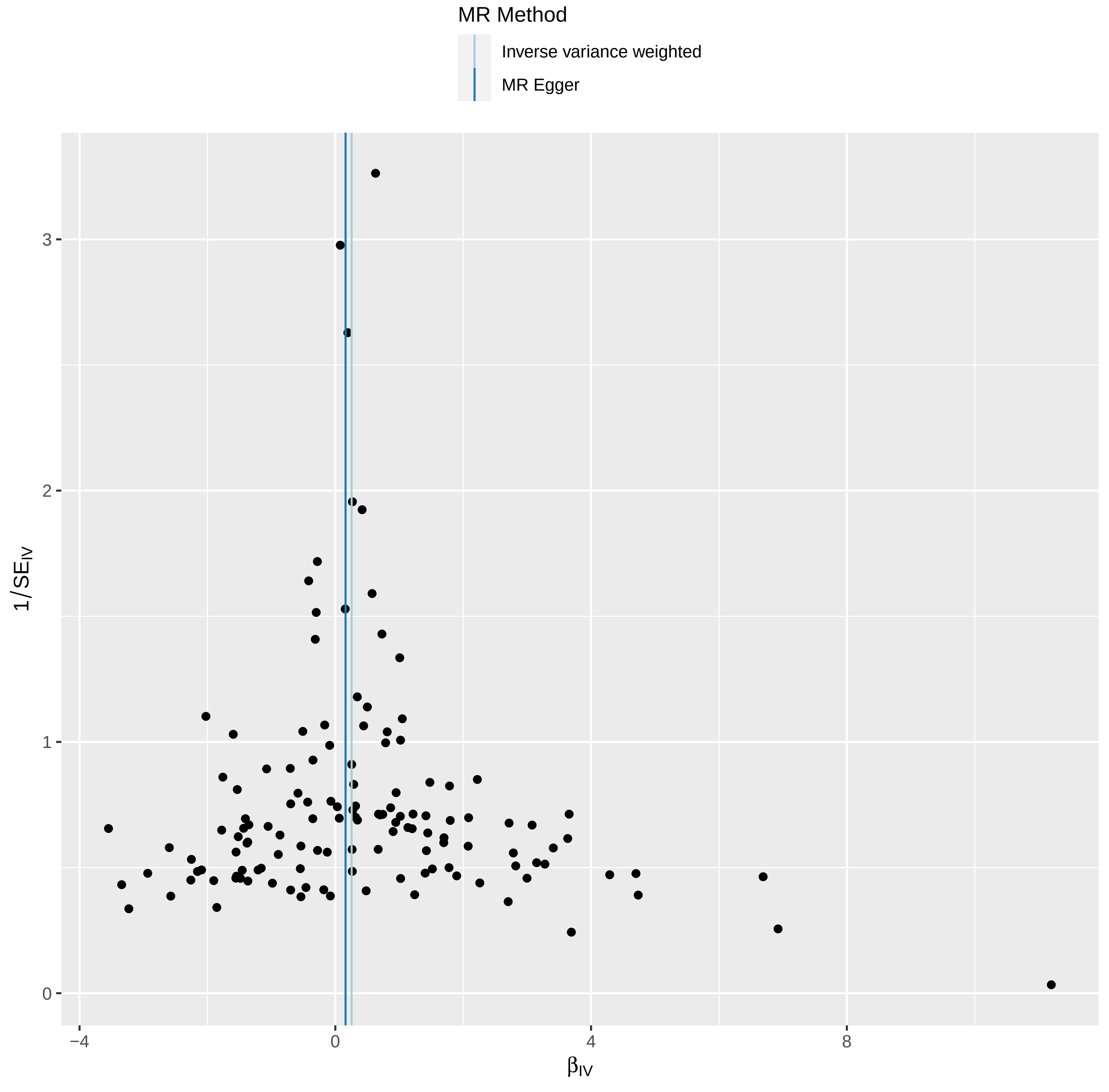
MR Method
Inverse variance weighted
MR Egger
3
2
1
0
1/SE_IV
−4
0
4
8
β_IV
