# Supplementary material for: Causal relationship between immune cells and telomere length: mendelian randomization analysis
Source: BMC Immunol. 2024 Mar 8;25:19. doi: 10.1186/s12865-024-00610-6 (PMC10924351; doi:10.1186/s12865-024-00610-6)
Supplement: Supplementary file 6 — Supplementary Material 6 [file 12865_2024_610_MOESM6_ESM.docx]

**legends:**

**Figure S1. Results of MR analysis of genetic causality between immune cells and telomere length.** MR: mendelian randomization.

**Figure S2. Sensitivity test for MR analysis of immune cells and telomere length.** Referring to the order of the immune cells in Table 2, the corresponding leave-one-out analysis plots, scatter plots, and funnel plots were plotted sequentially. Sensitivity analysis showed no horizontal pleiotropy or heterogeneity. MR: mendelian randomization.

**Figure S3. Results of MR analysis of genetic causality between telomere length and immune cells.** MR: mendelian randomization.

**Figure S4. Sensitivity test for MR analysis of telomere length and immune cells.** Referring to the order of the immune cells in Table 2, the corresponding leave-one-out analysis plots, scatter plots, and funnel plots were plotted sequentially. Sensitivity analysis showed no horizontal pleiotropy or heterogeneity. MR: mendelian randomization.

**Table S1.** Screening results of single nucleotide polymorphism.
